# Elucidating the Role and Mechanism of Alpha‐Enolase in Senescent Amelioration via Metabolic Reprogramming

**DOI:** 10.1111/cpr.70049

**Published:** 2025-04-27

**Authors:** Yun Haeng Lee, Hyunwoong Lim, Gyungmin Kim, Geonhee Jang, Myeong Uk Kuk, Ji Ho Park, Jee hee Yoon, Yoo Jin Lee, Duyeol Kim, Byeonghyeon So, Minseon Kim, Hyung Wook Kwon, Youngjoo Byun, Joon Tae Park

**Affiliations:** ^1^ Division of Life Sciences Incheon National University Incheon Republic of Korea; ^2^ College of Pharmacy Korea University Sejong Republic of Korea; ^3^ Interdisciplinary Major Program in Innovative Pharmaceutical Sciences Korea University Sejong Republic of Korea; ^4^ Convergence Research Center for Insect Vectors Incheon National University Incheon Republic of Korea

**Keywords:** alpha‐enolase, *Caenorhabditis elegans*, metabolic reprogramming, senescence amelioration

## Abstract

Senescent cells are characterised by increased glycolysis dependence. Normalisation of glycolysis metabolism is essential for senescence amelioration. However, the mechanism of proteins involved in cellular glycolysis metabolism has not been fully elucidated. Here, we identified a candidate compound, an oxazole analogue (KB2764), that can improve senescence. To elucidate the mechanism of the KB2764, we investigated the interacting proteins. KB2764 interacted with alpha‐enolase (ENO1) and pyruvate kinase M (PKM), ultimately allowing PKM to phosphorylate ENO1. KB2764 consequently increased mitochondrial ATP production and reduced reliance on glycolysis. Knockdown of the ENO1 experiment in senescent cells demonstrates that regulation of ENO1 activity is a prerequisite for recovery of mitochondrial function. Furthermore, the action of KB2764 extends its application to extend the lifespan of *Caenorhabditis elegans* . Taken together, our findings reveal a novel mechanism by which senescence is ameliorated through metabolic reprogramming and mitochondrial functional recovery via KB2764‐mediated regulation of ENO1 protein activity.

## Graphical abstract

KB2764 interacted with alpha‐enolase (ENO1) and pyruvate kinase M (PKM), ultimately leading to PKM phosphorylation of ENO1. KB2764 consequently increased mitochondrial ATP production and decreased glycolysis dependence. Furthermore, the action of KB2764 extends its application to extend the lifespan of *Caenorhabditis elegans* . Taken together, our findings reveal a novel mechanism by which senescence is ameliorated through metabolic reprogramming and mitochondrial functional recovery via KB2764‐mediated regulation of ENO1 protein activity.

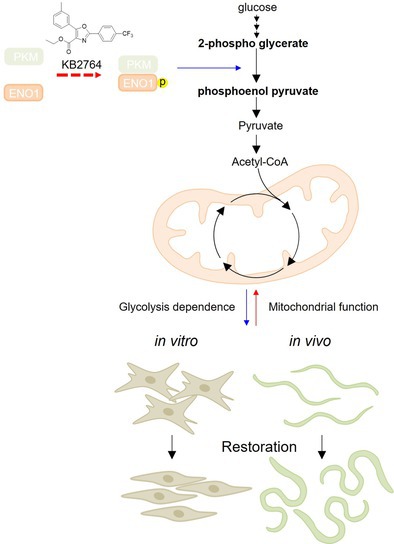

## 1 Introduction

Senescence refers to the loss of replicative ability that occurs when normal somatic cells are cultured for long periods of time [1]. Moreover, senescence is characterised by altered structure and function of cellular organelles, with mitochondria showing the most prominent changes [2]. Notable changes in mitochondria include an increase in volume and size. This increase is a feedback response that occurs because inefficient electron transport in the electron transport complex (ETC) generates reactive oxygen species (ROS), damaging mitochondrial structure and function [3]. Dysfunctional mitochondria are not only a major source of excessive ROS, but are also a major target of ROS, causing ETC damage that reduces the efficiency of oxidative phosphorylation (OXPHOS) [3]. Therefore, senescent cells rely more on glycolysis than OXPHOS as an intracellular energy source [3]. These findings are supported by evidence from investigations of energy metabolism in senescent cells showing increased lactate formation and glucose consumption, suggesting active glycolysis [4]. The close link between senescence and metabolic changes has been supported by the fact that metabolic changes cause premature deterioration of tissue and organ function [5]. However, the underlying mechanisms that can reverse metabolic changes in senescent cells are still unknown. Therefore, a fundamental understanding of metabolic regulation in senescent cells is necessary.

Oxazoles and pyrazoles are heterocyclic aromatic organic motifs that are widely introduced in pharmacologically active compounds [6]. Oxazole and pyrazole analogues have been found to be useful in reducing senescence [7]. For example, an oxazoloquinoline derivative (KB1541) bound to the 14‐3‐3ζ protein and then phosphorylated the protein at serine 58 residue [7]. This phosphorylation enhanced the ATP synthase 5 alpha/beta dimer, increasing oxidative phosphorylation (OXPHOS) efficiency and stimulating ATP production [7]. Then, improved OXPHOS efficiency resulted in the restoration of mitochondrial function and reduction of senescence [7]. Another oxazole‐based HUP‐55 affected autophagy, ROS generation and α‐synuclein dimerisation [8]. In mice with age‐elated Parkinson's disease, HUP‐55 crossed the blood–brain barrier and alleviated Parkinson's disease by inhibiting α‐synuclein dimerisation [8]. Recently, zoledronic acid, a pyrazole analogue, was found to have a senolytic effect that preferentially eliminates senescent cells and leaves young cells intact [9]. The senolytic effect of zoledronic acid was further confirmed by showing that zoledronic acid extended lifespan in progeria mice [9].

In this study, we aimed to identify compounds that can reverse ageing using a library containing oxazole and pyrazole analogues. We identified one candidate that enables cellular metabolic reprogramming through phosphorylation of alpha‐enolase (ENO1). Here, we propose a novel ageing reversal mechanism by this drug candidate.

## 2 Materials and Methods

Detailed materials and methods are described in Supporting Information S1.

## 3 Results

### 3.1 Screening of the Compound KB2764 That Ameliorates the Senescence Phenotype

**FIGURE 1:**
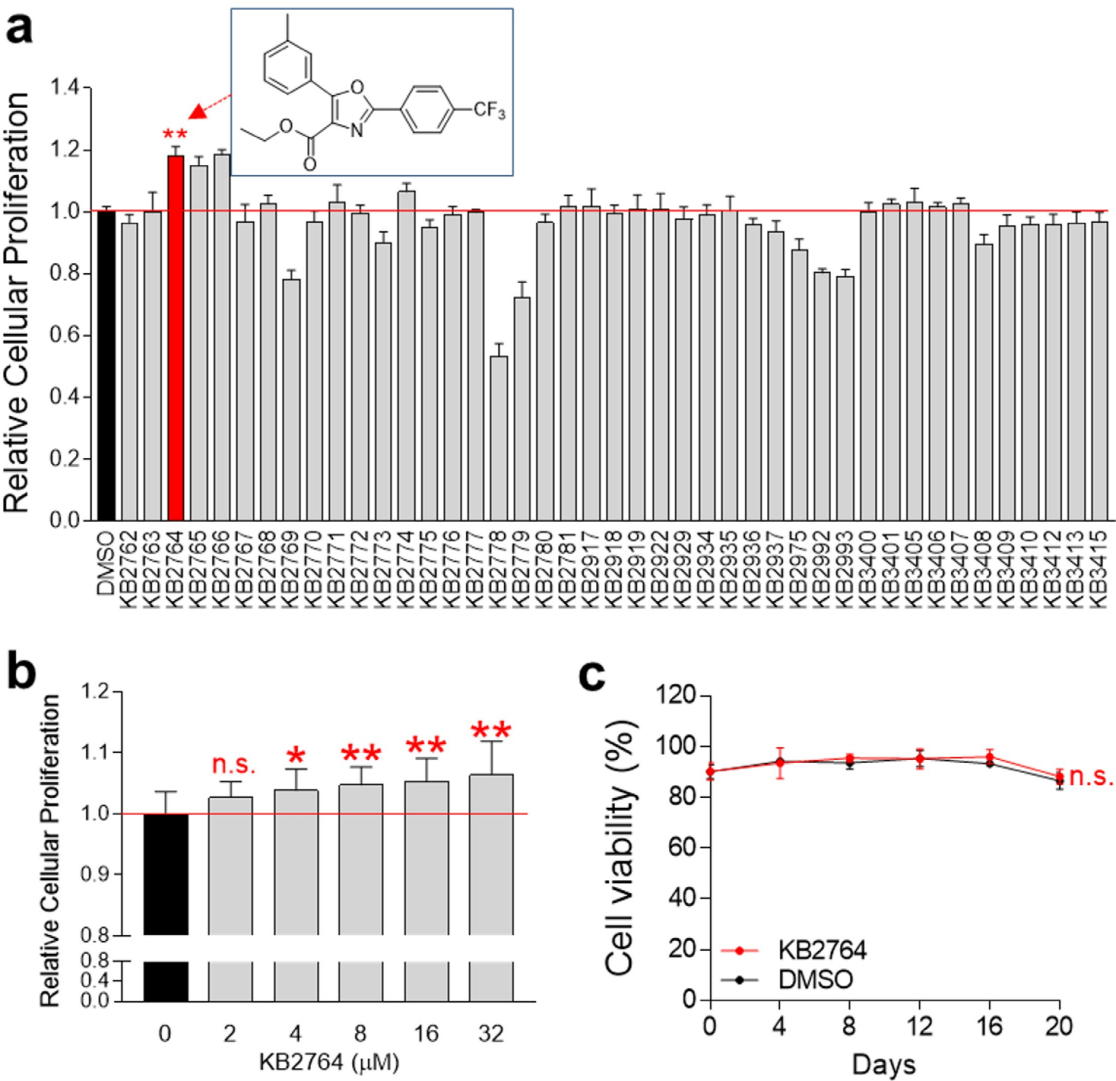
Screening of the compound KB2764 that ameliorates the senescence phenotype. (a) A quantitative assessment of cellular proliferation was conducted utilising a DNA content‐based method. **p < 0.01, Student's *t*‐test. Mean ± SD, *n* = 10. Chemical structure of an oxazole analogue (KB2764) was shown in a rectangle. (b) Cell proliferation was assessed at different concentrations of KB2764 (0–32 μM). n.s. (not significant), **p < 0.01, Student's *t*‐test. Mean ± SD, *n* = 10. (c) Cell viability was assessed to investigate the toxicity of KB2764 at concentrations of 8 μM. n.s. (not significant), two‐way ANOVA followed by Bonferroni's post‐test. Mean ± SD, *n* = 3.

In the current study, we used a screening method to measure cell proliferation to determine whether candidate compounds have the potential to reverse senescence. Senescent cells were treated with a library of oxazole and pyrazole analogues. The effect on cell number was assessed at Day 12 (Figure 1a). Among the 43 potential candidates, cells treated with KB2764 induced the highest cell number proliferation compared to cells treated with DMSO (red bar in Figure 1a). Therefore, KB2764 was selected as a potential candidate and subsequent experiments were performed. The structure of KB2764, an oxazole derivative, is shown inside the rectangle in Figure 1a. Figure S1 provides a full description of the synthetic strategy to prepare KB2764.

During screening experiments, all compounds were diluted to a final concentration of 8 μM. Therefore, KB2764, which was selected as a candidate, was also used after diluting to 8 μM. To determine the optimal KB2764 concentration to induce cell proliferation, KB2764 was diluted to several concentrations (0–32 μM). Cells were treated with KB2764 at different concentrations, and cell proliferation was measured. Starting from 4 μM, a significant effect on the induction of cell proliferation was observed (Figure 1b; *p < 0.05, **p < 0.01). The proliferation‐inducing effect was more significant at the 8 μM concentration (Figure 1b; **p < 0.01). Therefore, a concentration of 8 μM was selected and used for subsequent experiments.

We next investigated the toxicity of KB2764 at selected concentrations by assessing cell viability. Senescent cells treated with KB2764 showed a viability similar to that of senescent cells treated with DMSO (Figure 1c). These results indicate that the 8 μM concentration of KB2764 did not exhibit cytotoxicity.

### 3.2 KB2764 Interacts With ENO1 and Pyruvate Kinase M (PKM)

**FIGURE 2:**
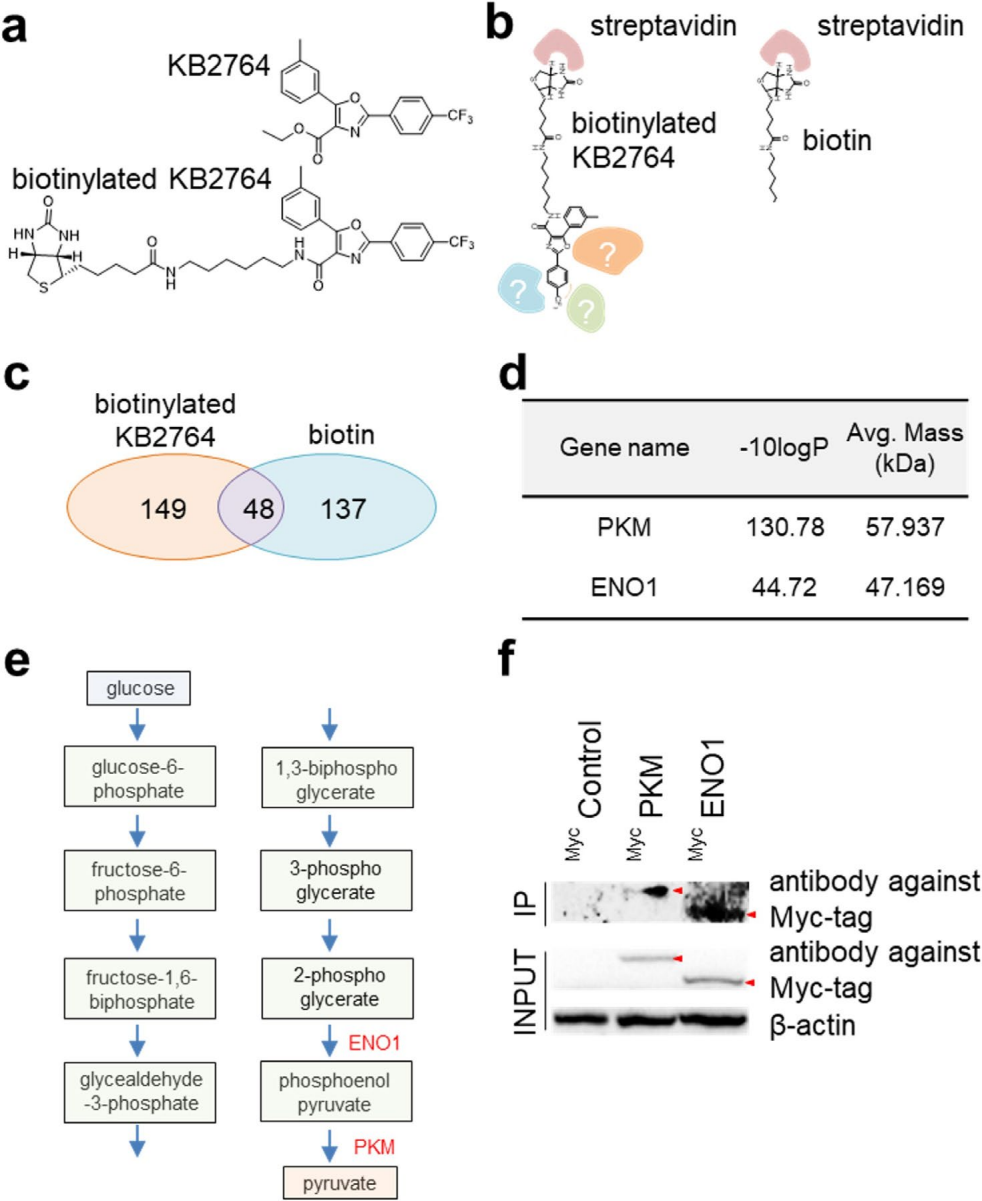
Identification of KB2764 interacting proteins. (a) Chemical structure of biotinylated KB2764. (b) Schematic diagram of immunoprecipitation workflow using biotinylated KB2764 or biotin. (c) Ion trap mass spectrometry was used to identify proteins that bind to biotinylated KB2764 or biotin. The Venn diagram shows the number of proteins to which biotinylated KB2764 binds and the number of proteins to which biotin binds. The detailed protein information is described in Tables S1–S3. (d) Selected candidates are shown. –10logP: –10log(*p* value), Avg. Mass (kDa): Average of protein molecular mass. (e) Schematic representation of the glycolysis pathway. PKM catalyses the conversion of phosphophenol pyruvate to pyruvate. ENO1 catalyses the conversion of 2‐phosphoglycerate to phosphophenol pyruvate. (f) Co‐precipitation of biotinylated KB2764 and Myc‐tagged candidate proteins (Myc‐tagged control, Myc‐tagged PKM or Myc‐tagged ENO1 proteins). Primary antibodies utilised in the immunoblotting procedure were antibodies against Myc‐tag and β‐actin.

KB2764 was found to increase cell proliferation of senescent cells, but it is unclear what role KB2764 plays in senescence recovery. Therefore, we decided to gain information about KB2764 by identifying its binding interaction partners. To use KB2764 as bait in the pull‐down assay, KB2764 was biotinylated (Figure 2a). The synthetic procedure to prepare biotinylated KB2764 is shown in Figures S2–S5. To precipitate KB2764‐binding proteins, pull‐down experiments were performed using biotinylated KB2764. The biotinylated KB2764 bound to streptavidin and co‐precipitated KB2764‐bound proteins (Figure 2b). As a control, pull‐down experiments were performed using only biotin (Figure 2b). Ion trap mass spectrometry was used to identify proteins that bind to biotinylated KB2764 or biotin. One hundred forty‐nine proteins bound only to biotinylated KB2764 and not to biotin (Figure 2c and Table S1). One hundred thirty‐seven proteins bound only to biotin and not to biotinylated KB2764 (Figure 2c and Table S2). Both biotinylated KB2764 and biotin bound to 48 proteins (Figure 2c and Table S3).

To find proteins associated with ageing recovery among the 149 proteins that bind only to biotinylated KB2764, we considered two factors. First, the *p* value, which is the probability that the measured mass value is a random match that does not match a mass value in the protein database, was considered [10]. To visualise the *p* value, the *p* value was converted to −10log (*p* value) (Figure 2d and Table S1). A lower *p* value corresponds to a larger −10log (*p* value), indicating that the match is less likely to have occurred due to random matching. Second, the association of the selected protein with senescence was considered. ENO1 and PKM were selected as the most promising candidates because they showed high −10log (*p* value) and are proteins involved in glycolysis, which plays an important role in senescence [11] (Figure 2d). Specifically, PKM catalyses the conversion of phosphophenol pyruvate to pyruvate, and ENO1 catalyses the conversion of 2‐phosphoglycerate to phosphophenol pyruvate [12, 13] (Figure 2e).

In vivo pull‐down assay was used to confirm the binding between KB2764 and candidate proteins. HEK293T cells were transfected using pCMV‐Myc‐PKM or pCMV‐Myc‐ENO1 plasmids and then grown in a medium containing biotinylated KB2764. Cell lysates were then precipitated using streptavidin, and immunoblotting was performed using an antibody against Myc‐tag. In vivo pull‐down analysis showed that biotinylated KB2764 bound to Myc‐tagged PKM or Myc‐tagged ENO1 proteins, validating the mass spectrometry results (Figure 2f).

We then used an assay called microscale thermophoresis (MST) to determine which candidate proteins bound most tightly to KB2764. Various concentrations of KB2764, along with His‐tagged PKM protein or His‐tagged ENO1 protein, were used for testing. The dissociation constant (*K*_d_) between KB2764 and PKM was 9.32 μM, indicating a weak interaction between them (Figure S6a). On the other hand, KB2764 and ENO1 had a *K*_d_ of 40.09 nM, indicating a strong interaction between them (Figure S6b). These findings indicate that KB2764 is bound to both ENO1 and PKM proteins and more strongly to ENO1 protein.

We then performed an in silico docking study to predict the potential binding mode of KB2764 with ENO1 (PDB ID: 3B97). Docking studies showed that the best‐docked pose of KB2764 interacted with Arg 253 and Ser 254 of ENO1 (Figure S7a). Specifically, the oxazole and ester moieties of KB2764 interacted with Ser 254 of ENO1. Additionally, the 3‐methylphenyl moiety of KB2764 created a cation–π interaction with Arg 253 of ENO1 (Figure S7a). These data suggest that KB2764 can bind tightly to ENO1 via Arg 253 and Ser 254. To test whether Ser 254 in ENO1 plays an important role in the binding between KB2764 and ENO1, MST was performed using KB2764 and ENO1 (S254A) proteins. Various concentrations of KB2764, along with His‐tagged ENO1 (S254A) protein, were used for testing. *K*_d_ between KB2764 and ENO1 (S254A) proteins was 0.24 mM (Figure S7b). However, this *K*_d_ value was much larger than the *K*_d_ value between KB2764 and ENO1 (WT) proteins (40.09 nM), indicating that Ser 254 of ENO1 plays an important role in the binding between KB2764 and ENO1 protein (Figure S7b).

### 3.3 KB2764 Increases the Interaction Between PKM and ENO1, Ultimately Allowing PKM to Phosphorylate ENO1

**FIGURE 3:**
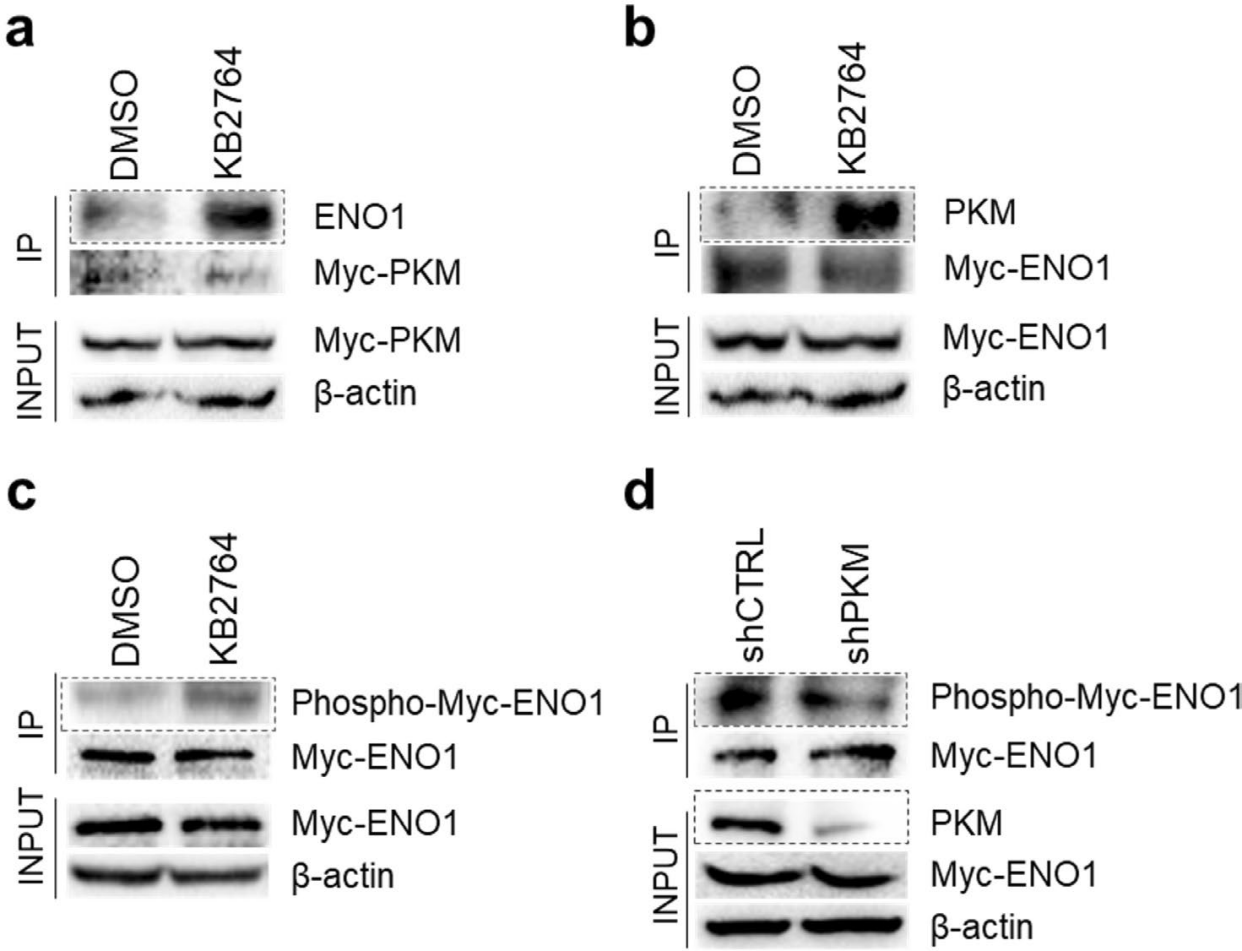
KB2764 increases the interaction between PKM and ENO1, ultimately allowing PKM to phosphorylate ENO1. (a) Effect of KB2764 on the interaction between PKM and ENO1. KB2764 increased the precipitation of endogenous ENO1 protein (red‐boxed areas). (b) Confirmation of the effect of KB2764 on the interaction between PKM and ENO1. KB2764 increased the precipitation of endogenous PKM protein (red‐boxed areas). (c) Effect of KB2764 on PKM‐mediated ENO1 phosphorylation. ENO1 phosphorylation was increased upon KB2764 treatment (red‐boxed areas). (d) Effect of PKM knockdown on ENO1 phosphorylation. shPKM‐mediated ENO1 knockdown reduced the endogenous expression level of PKM protein (blue‐boxed areas). ENO1 phosphorylation was decreased in the shPKM group (red‐boxed areas).

Protein kinases target and phosphorylate specific substrates through physical interactions [14]. Since PKM is a protein kinase, it is possible that KB2764 increases the interaction between PKM and ENO1, ultimately allowing PKM to phosphorylate ENO1. Therefore, we first investigated the effect of KB2764 on the interaction between PKM and ENO1. To examine the effect, pCMV‐Myc‐PKM was transfected into HEK293T cells. Transfected HEK293T cells were treated with DMSO or KB2764 and then immunoprecipitated with Myc‐tag antibody. Immunoprecipitated Myc‐PKM levels in cells treated with KB2764 were identical to those in cells treated with DMSO (Figure 3a). However, KB2764 increased the precipitation of endogenous ENO1 protein, indicating that KB2764 increased the interaction between PKM and ENO1 proteins (red box in Figure 3a). To confirm the effect of KB2764 on the interaction between PKM and ENO1, pCMV‐Myc‐ENO1 was transfected into HEK293T cells. Transfected cells were treated with DMSO or KB2764 and then immunoprecipitated with Myc‐tag antibody. Immunoprecipitated Myc‐ENO1 levels in cells treated with KB2764 were identical to those in the control group (Figure 3b). However, KB2764 increased the precipitation of endogenous PKM protein, indicating that KB2764 increased the interaction between PKM and ENO1 proteins (red box in Figure 3b). Next, we investigated the effect of KB2764 on PKM‐mediated ENO1 phosphorylation. A phospho‐Ser antibody was used to detect the phosphorylation of ENO1. In the KB2764 group compared to the DMSO group, immunoprecipitated ENO1 showed an increased phosphorylated form (red box in Figure 3c). These results indicate that KB2764 increases PKM‐mediated ENO1 phosphorylation.

The finding that KB2764 increases PKM‐mediated ENO1 phosphorylation prompted us to investigate the effect of PKM knockdown on ENO1 phosphorylation. After transfecting HEK293T cells with pCMV‐Myc‐ENO1, the expression level of PKM was suppressed using shRNA that directly targets PKM (shPKM). PKM protein knockdown was assessed following transduction with lentivirus expressing shPKM or control shRNA (shCTRL). shPKM‐mediated ENO1 knockdown reduced the endogenous expression level of PKM protein, whereas shCTRL did not affect the expression (blue box in Figure 3d). Then, the cells were immunoprecipitated with Myc‐tag antibody. Immunoprecipitated Myc‐ENO1 levels in cells treated with shPKM were identical to those treated with shCTRL (Figure 3d). Next, a phospho‐Ser antibody was used to detect the phosphorylation of ENO1. In the shPKM group, immunoprecipitated ENO1 showed a decreased phosphorylated form (red box in Figure 3d). These results indicate that inhibition of endogenous PKM expression reduces ENO1 phosphorylation, supporting a role for PKM in ENO1 phosphorylation.

The discovery of KB2764‐mediated phosphorylation sites in ENO1 will further shed light on the mechanism of action of ENO1. Therefore, we used the Kinase Prediction Tool (http://www.cbs.dtu.dk/services/NetPhos/) to predict potential phosphorylation motifs in ENO1. Potential phosphorylation motifs were predicted to be serines (Ser) 254, 272 and 353, which are conserved across species (Figure S8a). To test the prediction, serine to alanine point mutations (S254A, S272A and S353A) were introduced into ENO1. Cells were transfected with pCMV‐Myc‐ENO1 (WT), pCMV‐Myc‐ENO1 (S254A), pCMV‐Myc‐ENO1 (S272A) or pCMV‐Myc‐ENO1 (S353A). Immunoprecipitation was performed on cell lysates using an antibody against Myc‐tag. The phosphorylation was detected by phospho‐Ser antibody. Phosphorylation of ENO1 (S254A) and ENO1 (S272A) was not reduced compared to ENO1 (WT), indicating that Ser254 and 272 are not potential phosphorylation motifs (blue box in Figure S8b). However, phosphorylation of ENO1 (S353A) was markedly reduced compared to ENO1 (WT), indicating Ser353 as the potential phosphorylation motif (red box in Figure S8b).

### 3.4 KB2764‐Mediated Regulation of ENO1 Activity Induces Metabolic Reprogramming

**FIGURE 4:**
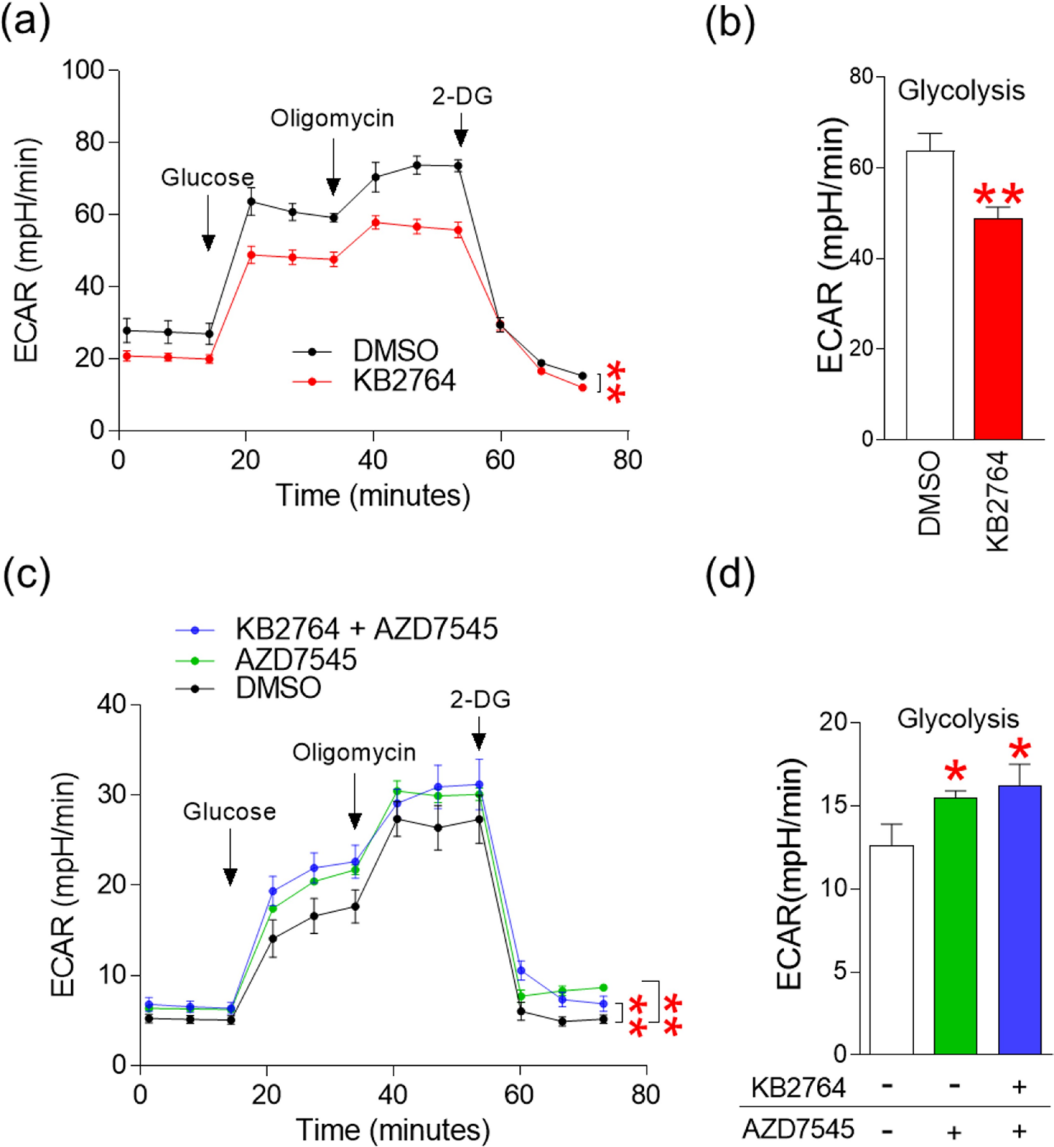
KB2764 decreases dependence on glycolysis as an energy source. (a) Measurement of ECAR (black line: DMSO‐treated senescent cells, red line: KB2764‐treated cells). **p < 0.01, two‐way ANOVA followed by Bonferroni's post‐test. Means ± SD, *n* = 3. (b) Measurement of the glycolysis level. **p < 0.01, Student's *t*‐test. Means ± SD, *n* = 3. (c) Measurement of ECAR (black line: DMSO‐treated senescent cells, green line: AZD7545‐treated senescent cells, red line: KB2764‐ and AZD7545‐cotreated cells). n.s. (not significant), **p < 0.01, two‐way ANOVA followed by Bonferroni's post‐test. Means ± SD, *n* = 3. (d) Measurement of the glycolysis level. n.s. (not significant), *p < 0.05, Student's *t*‐test. Means ± SD, *n* = 3.

Senescent cells are characterised by inefficient ATP production due to dysfunctional mitochondria [15]. Therefore, senescent cells increase ATP production via glycolysis to compensate for reduced ATP levels and become more dependent on glycolysis. Since PKM and ENO1 are proteins involved in glycolysis, we speculated that the increased interaction between PKM and ENO1 by KB2764 would decrease dependence on glycolysis. To prove our assumption, we assessed the level of glycolysis, which can be measured by extracellular acidification rate (ECAR). ECAR can be tested by sequentially administering chemicals such as glucose, oligomycin and 2‐deoxy‐d‐glucose (2‐DG). After injection of glucose and oligomycin, the ECAR values of KB2764‐treated senescent cells were significantly lower than those of DMSO‐treated senescent cells, indicating that KB2764 lowered the level of glycolysis (Figure 4a,b). These data suggest that KB2764‐treated senescent cells are less dependent on glycolysis as an energy source than DMSO‐treated senescent cells.

We investigated whether KB2764 treatment is a prerequisite for reducing dependence on glycolysis in senescent cells. AZD7545 is an inhibitor of pyruvate dehydrogenase kinase 2 and inhibits OXPHOS by blocking acetyl‐CoA production [16]. AZD7545 was used to inhibit OXPHOS in senescent cells. AZD7545‐treated senescent cells had significantly increased ECAR values compared to DMSO‐treated senescent cells, indicating that AZD7545 treatment inhibited OXPHOS and induced metabolic reprogramming toward glycolysis (Figure 4c,d; green lines and green bar). Additionally, senescent cells cotreated with KB2764 and AZD7545 also showed significantly increased ECAR values compared to senescent cells treated with DMSO (Figure 4c,d; blue lines and blue bars). This result indicates that AZD7545 prevented the decrease in glycolysis levels caused by KB2764. However, senescent cells co‐treated with KB2764/AZD7545 showed no difference in ECAR compared to senescent cells treated with AZD7545 (Figure 4c,d; green lines vs. blue lines. green bars vs. blue bars). These results confirmed that AZD7545 blocked KB2764‐induced decrease in glycolysis levels. Taken together, these data suggest that KB2764 treatment is a prerequisite for reducing dependence on glycolysis in senescent cells.

**FIGURE 5:**
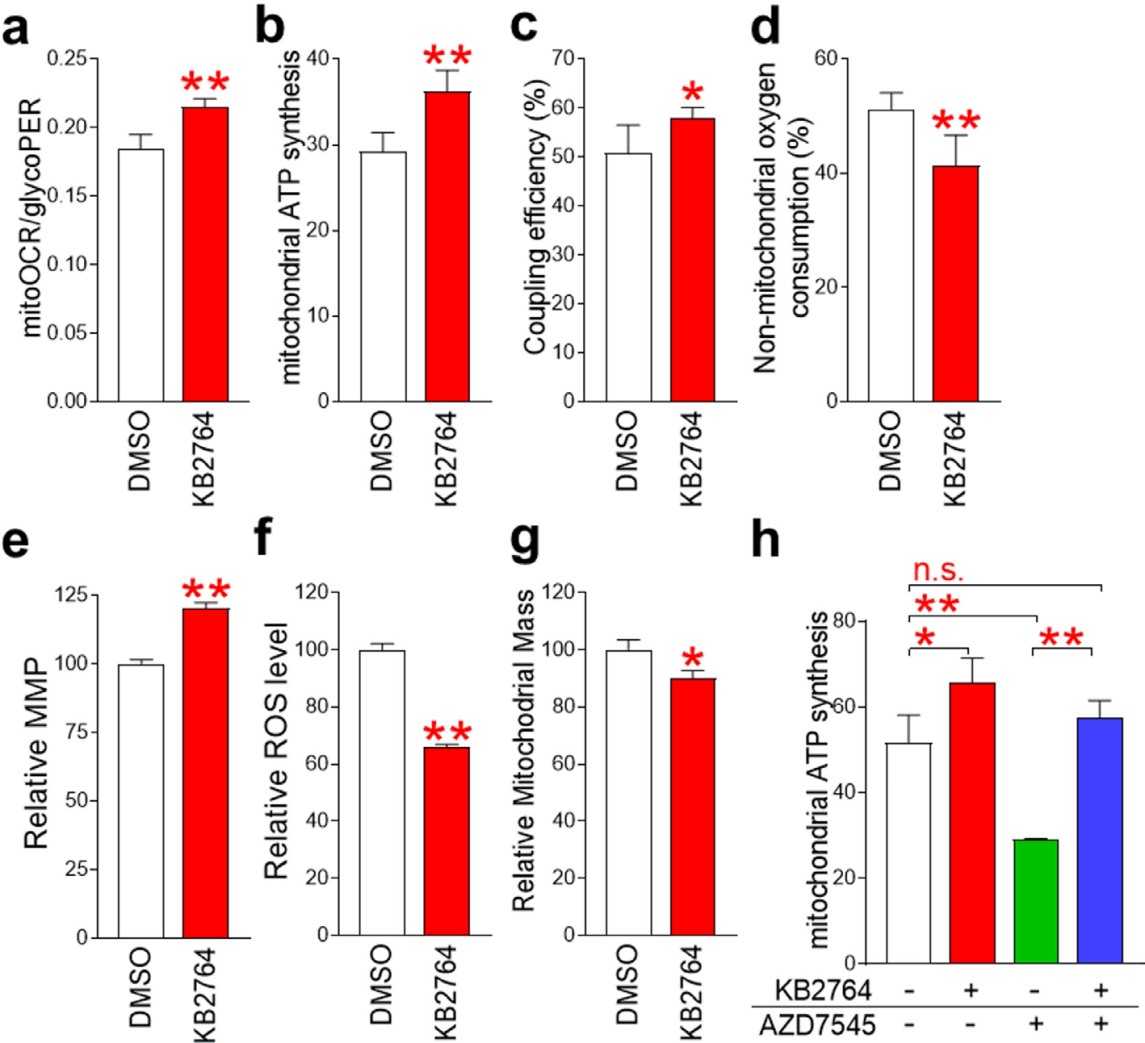
KB2764‐mediated regulation of ENO1 activity induces metabolic reprogramming. (a) Comparison of mitoOCR/glycoPER in senescent cells treated with DMSO or KB2764. **p < 0.01, Student's *t*‐test. Mean ± SD, *n* = 3. (b) Comparison of mitochondrial ATP synthesis in senescent cells treated with DMSO or KB2764. **p < 0.01, Student's *t*‐test. Mean ± SD, *n* = 3. (c) Comparison of the coupling efficiency (i.e., the ratio of oxygen consumed driving ATP synthesis compared to driving proton leak) in senescent cells treated with DMSO or KB2764. *p < 0.05, Student's *t*‐test. Mean ± SD, *n* = 3. (d) Comparison of non‐mitochondrial oxygen consumption in senescent cells treated with DMSO or KB2764. **p < 0.01, Student's *t*‐test. Mean ± SD, *n* = 3. (e) Flow cytometric analysis of mitochondrial membrane potential (MMP) using JC‐10. **p < 0.01, Student's *t*‐test. Mean ± SD, *n* = 3. (f) Flow cytometric analysis of reactive oxygen species (ROS) using DHR123. **p < 0.01, Student's *t*‐test. Mean ± SD, *n* = 3. (g) Flow cytometric analysis of mitochondrial mass using MitoTracker green. **p < 0.01, Student's *t*‐test. Mean ± SD, *n* = 3. (h) Comparison of mitochondrial ATP synthesis in senescent cells treated with ‘DMSO’, ‘KB2764’, ‘AZD7545’ or ‘KB2764 and AZD7545’. n.s. (not significant), *p < 0.05, **p < 0.01, Student's *t*‐test. Mean ± SD, *n* = 3.

The observation of decreased glycolysis dependence by KB2764 raised the question of how mitochondria respond to maintain intracellular energy homeostasis. To determine whether mitochondria contribute to energy homeostasis, mitoOCR/glycoPER values, which indicate the ratio of OXPHOS to glycolysis, were measured. mitoOCR/glycoPER values were significantly increased by KB2764 treatment, indicating increased mitochondrial contribution to energy homeostasis (Figure 5a). The increased contribution was evidenced by the finding that KB2764 increased mitochondrial ATP synthesis (Figure 5b). To confirm this result, we examined the coupling efficiency of mitochondria (the ratio of oxygen consumption driving ATP synthesis compared to driving proton leak). KB2764 significantly increased coupling efficiency, indicating that KB2764 increased the proportion of oxygen consumption to drive ATP synthesis (Figure 5c). We then examined non‐mitochondrial oxygen consumption to determine whether the increase in ATP synthesis induced by KB2764 was mitochondrial. KB2764 significantly reduced non‐mitochondrial oxygen consumption, indicating that the increase in ATP synthesis was primarily mitochondrial (Figure 5d). The mitochondrial membrane potential (MMP) is known to maintain the electrochemical potential of hydrogen ions required for ATP synthesis [17]. KB2764 significantly increased MMPs compared to the DMSO control, providing the underlying mechanism for the increased ATP synthesis by KB2764 (Figure 5e). Enhanced efficiency of mitochondrial ATP production is known to reduce mitochondrial ROS production [18]. Indeed, KB2764 significantly reduced ROS levels compared to DMSO‐treated senescent cells, indicating KB2764‐mediated restoration of mitochondrial function (Figure 5f). The increase in mitochondrial mass is known to be the result of a compensatory response to increased ROS and subsequent increased damaged mitochondria [19]. Because we observed a decrease in ROS levels by KB2764, we investigated whether there were changes in mitochondrial mass by KB2764. Senescent cells treated with KB2764 significantly reduced mitochondrial mass compared to senescent cells treated with DMSO (Figure 5g).

We then investigated whether the restoration of mitochondrial function induced by KB2764 affected mitochondrial ATP synthesis. AZD7545 was used to inhibit OXPHOS. KB2764 treatment significantly increased mitochondrial ATP synthesis, confirming the results shown in Figure 5b, whereas AZD7545 treatment decreased mitochondrial ATP synthesis (Figure 5h). Senescent cells were then co‐treated with KB2764/AZD7545. Compared with senescent cells treated with DMSO, senescent cells co‐treated with KB2764/AZD7545 did not show a significant increase in mitochondrial ATP synthesis (Figure 5h). To investigate the underlying mechanism, mitochondrial ATP synthesis in senescent cells co‐treated with KB2764/AZD7545 was compared with that in senescent cells treated with AZD7545. Senescent cells co‐treated with KB2764/AZD7545 showed a significant increase in mitochondrial ATP synthesis compared to senescent cells treated with AZD7545 alone (Figure 5h). These results suggest that KB2764, which directly affects OXPHOS by increasing mitochondrial binding efficiency and MMP, acts on mitochondrial ATP synthesis through a different mechanism than AZD7545, which acts as an indirect OXPHOS inhibitor by inhibiting acetyl‐CoA production.

**FIGURE 6:**
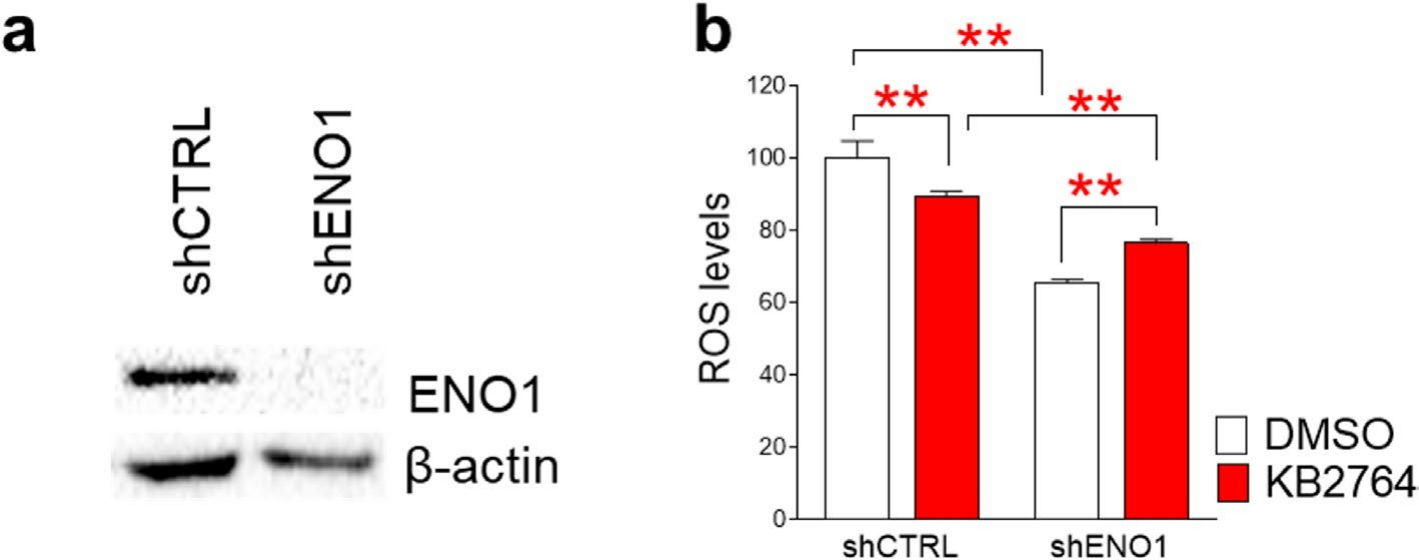
ENO1 expression is a prerequisite for KB2764‐mediated mitochondrial functional recovery. (a) Immunoblots of lysates from senescent cells transfected with lentivirus for control shRNA (shCTRL) or ENO1 shRNA (shENO1). Primary antibodies utilised in the immunoblotting procedure were against ENO1 and β‐actin. (b) Senescent cells transduced with shCTRL or shENO1 were treated with DMSO and KB2764. ROS levels, as indicative of mitochondrial functional recovery, were measured. Flow cytometric analysis of reactive oxygen species (ROS) using DHR123. **p < 0.01, Student's *t*‐test. Mean ± SD, *n* = 3.

Observing the restoration of mitochondrial function by KB2764, we investigated whether regulation of ENO1 by KB2764 is required for the restoration of mitochondrial function. To determine the role of KB2764‐mediated regulation of ENO1, endogenous ENO1 expression was suppressed using shRNA that directly targets ENO1 (shENO1). shENO1‐mediated ENO1 knockdown decreased the expression level of ENO1 protein, whereas shCTRL had no effect on the expression level of ENO1 protein (Figure 6a). Then, senescent cells transduced with lentivirus expressing shCTRL or shENO1 were treated with DMSO or KB2764. To investigate the restoration of mitochondrial function, ROS levels were measured. Senescent cells transduced with lentivirus expressing shCTRL responded to KB2764, as shown by a significant decrease in ROS levels (Figure 6b). However, senescent cells transduced with lentivirus expressing shENO1 did not reduce ROS levels after KB2764 treatment, unlike senescent cells transduced with lentivirus expressing shCTRL (Figure 6b). These results indicate that suppression of ENO1 expression via shRNA did not effectively reduce ROS levels by reducing the amount of ENO1 directly affected by KB2764. These results also suggest that KB2764‐mediated regulation of ENO1 is required for the restoration of mitochondrial function.

To determine whether knockdown of ENO1 expression affects ROS levels in senescent cells, ROS levels between senescent cells transduced with lentivirus expressing shCTRL and shENO1 were compared after treatment with DMSO or KB276. In the DMSO‐treated group, senescent cells transduced with lentivirus expressing shENO1 showed significantly lower ROS levels than senescent cells transduced with lentivirus expressing shCTRL (Figure 6b). Moreover, in the KB2764‐treated group, senescent cells transduced with lentivirus expressing shENO1 showed significantly lower ROS levels than senescent cells transduced with lentivirus expressing shCTRL (Figure 6b). These data indicate that ENO1 inhibition reduced ROS levels in senescent cells, regardless of whether treated with DMSO or KB2764.

To identify the interaction partners that bind to KB2764, we performed biotinylation on KB2764. We investigated the function of biotinylated KB2764 to rule out the possibility that biotinylation impairs the function of KB2764. Among the various effects of KB2764, we focused on its effect on intracellular ROS levels. Biotinylated KB2764 also significantly reduced ROS levels compared to DMSO‐treated senescent cells (Figure S9). These data indicate that biotinylation of KB2764 did not impair the function of KB2764, especially its ability to reduce ROS levels.

### 3.5 KB2764 Ameliorates Senescent Phenotypes

**FIGURE 7:**
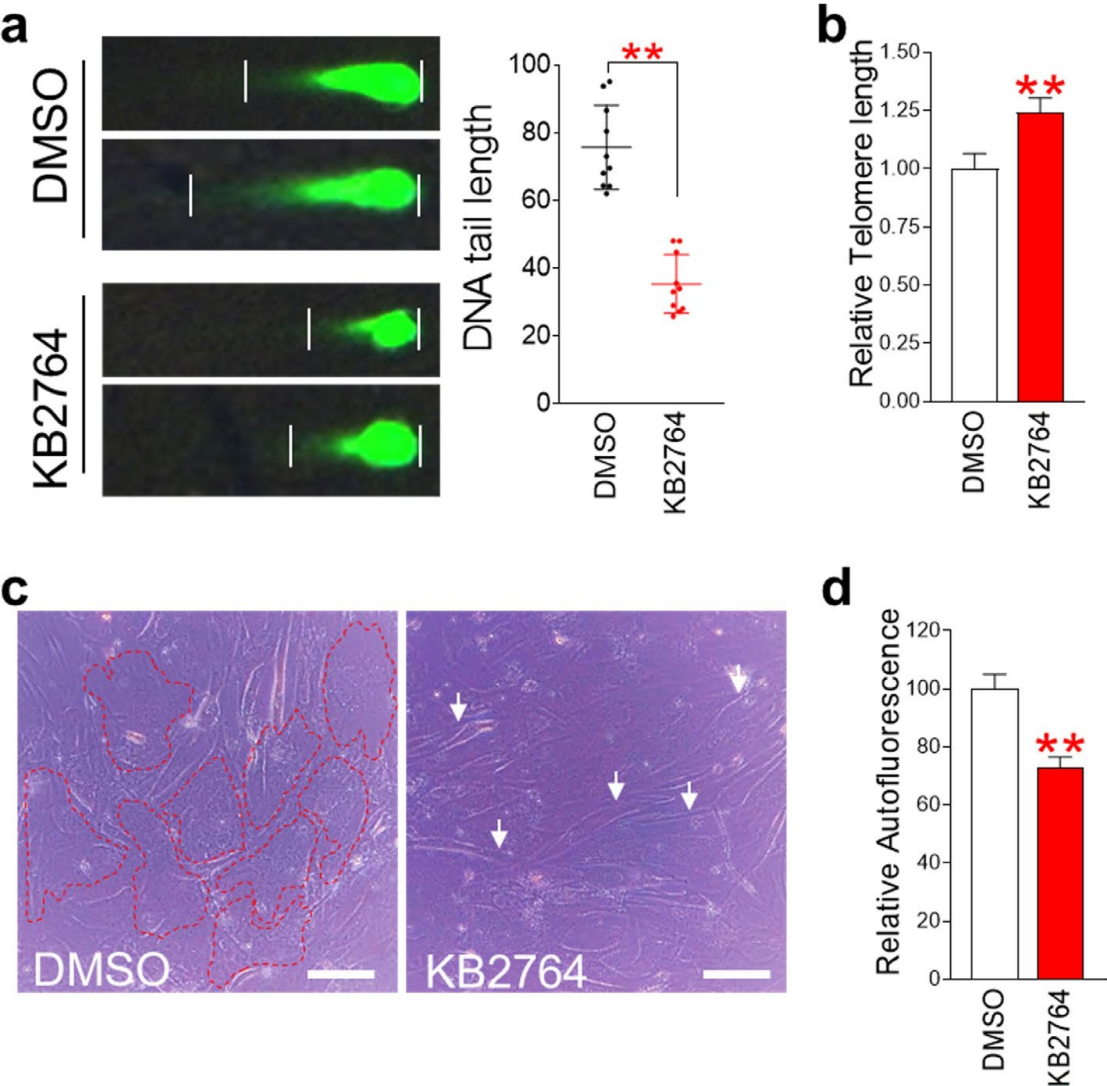
KB2764 ameliorates senescence phenotypes. (a) DNA tail length analysis of senescent cells treated with DMSO and KB2764. For DNA tail length analysis, comet assay was used. **p < 0.01, Student's *t*‐test. Mean ± SD, *n* = 10. (b) Comparison of DNA telomere length using quantitative PCR. **p < 0.01, Student's *t*‐test. Mean ± SD, *n* = 4. (c) Morphological changes in senescent cells after DMSO or KB2764 treatment. DMSO‐treated senescent cells showed broad and flat structures (dotted line), whereas treatment with KB2764 markedly recovered the morphology of senescent cells to a tiny spindle‐shaped structure (white arrows). (d) Flow cytometric analysis of autofluorescence. **p < 0.01, Student's *t*‐test. Mean ± SD, *n* = 3.

DNA double‐strand breaks (DSBs) increase with senescence and are widely used as a marker of senescence [20]. The main cause of DNA DSBs is DNA damage caused by excessive intracellular ROS [21]. Because we observed a reduction in ROS levels by KB2764, we speculated that this effect might reduce DNA DSB levels. A neutral comet assay was performed to investigate DNA DSB levels.KB2764‐treated senescent cells showed a significant decrease in DNA tail length compared to DMSO‐treated senescent cells, indicating reduced DNA DSB levels (Figure 7a).

Telomeres are repetitive DNA sequences located at the terminal regions of chromosomes and serve to protect the chromosomes from potential damage [22]. Excessive intracellular ROS damages telomeres, leading to a reduction in telomere length [22]. Therefore, the finding of the reduction of DNA DSB levels by KB2764 led us to also examine telomere length. Telomere length was measured using quantitative PCR. Senescent cells treated with KB2764 showed longer telomere lengths than those treated with DMSO (Figure 7b).

Restoration of mitochondrial function is known to be a prerequisite for improving senescence [11]. The observation of KB2764‐mediated restoration of mitochondrial function led us to investigate its impact on senescent phenotypes. As one of the notable hallmarks of senescence is an increase in cell surface area [23], we investigated the effect of KB2764 on the morphology of senescent cells. Senescent cells treated with DMSO showed a broad shape, which is one of the characteristics of senescent cells (dotted line in Figure 7c). On the other hand, senescent cells treated with KB2764 showed a small spindle shape, one of the characteristics of young cells (white arrow in Figure 7c).

Restoration of cell shape led us to investigate how KB2764‐mediated restoration of mitochondrial function affects lipofuscin levels. Lipofuscin is defined as a fluorescent lipid‐containing pigment that accumulates in the cytoplasm during senescence [24]. Lipofuscin levels were measured by assessing the intracellular amount of autofluorescence. Senescent cells treated with KB2764 showed a significant decrease in autofluorescence levels compared to senescent cells treated with DMSO (Figure 7d).

### 3.6 KB2764 Extends the Lifespan of *Caenorhabditis elegans*

**FIGURE 8:**
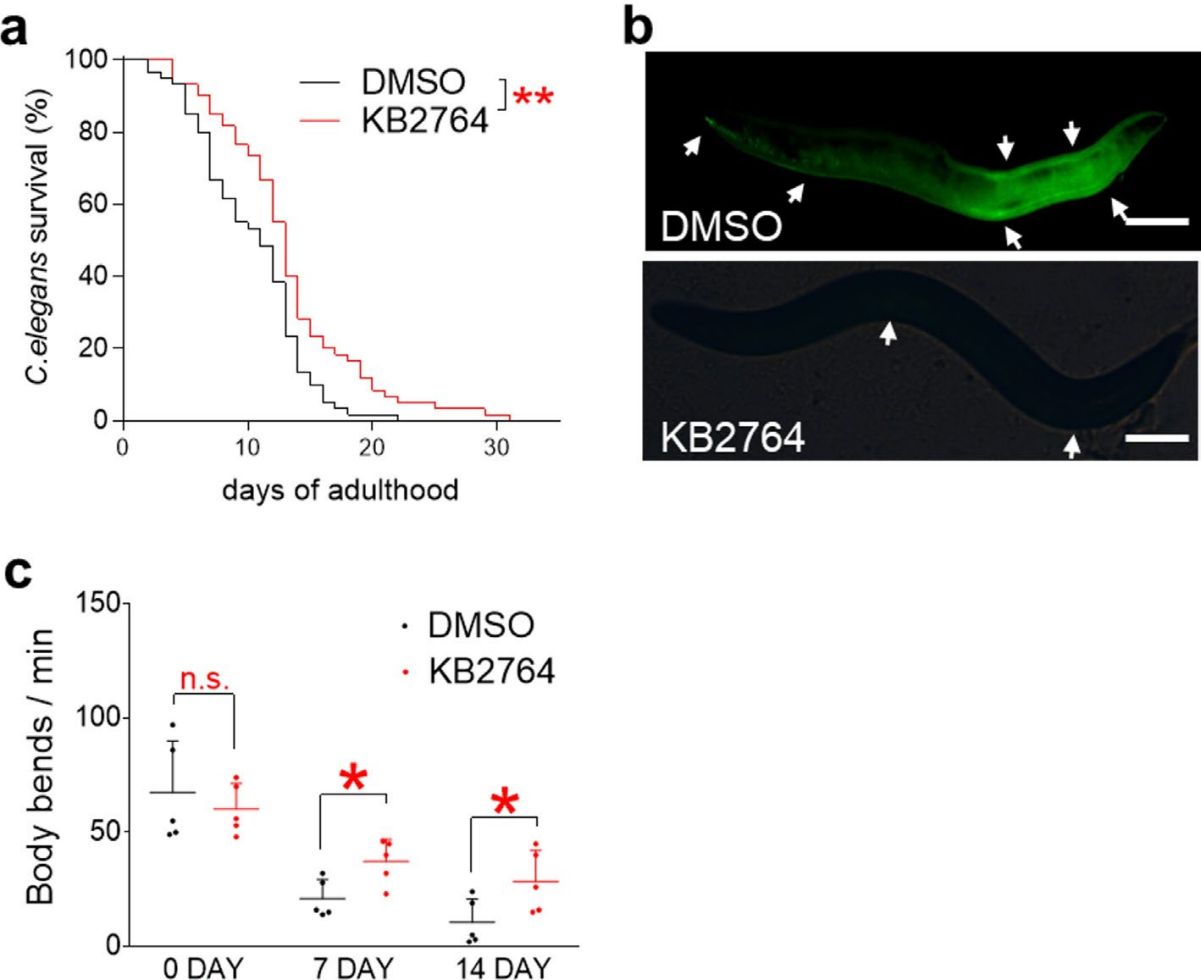
KB2764 extends the lifespan of *Caenorhabditis elegans* . (a) Survival analysis was performed while continuously treating synchronised *C. elegans* with DMSO or KB2764 (100 μM). KB2764 extended the lifespan of *C. elegans* by an average of 3 days. **p < 0.01, Kaplan–Meier analysis., *n* = 60. (b) After treating synchronised *C. elegans* with DMSO or KB2764 (100 μM) for 10 days, lipofuscin was observed under a fluorescence microscope. More lipofuscin was observed in aged *C. elegans* (white arrows). (c) The body bends were observed on Days 0, 7 and 14. The body bend was measured as one reciprocating motion and was evaluated as the number of times per minute. n.s (not significant), *p < 0.05, Student's *t*‐test. Mean ± SD, *n* = 5.

KB2764‐mediated improvement in senescence led us to investigate the effects of KB2764 in an in vivo model. *C. elegans* has been widely used in ageing studies because it is easy to observe and has a short lifespan [25]. Therefore, we investigated the effect of KB2764 on lifespan in *C. elegans* . KB2764 increased the average lifespan by 2 days, suggesting a marked increase considering that the lifespan of *C. elegans* is approximately 20 days (Figure 8a).

After observing KB2764‐mediated lifespan extension, we investigated other ageing markers. Similar to senescent cells, lipofuscin also accumulates in the cytoplasm during ageing in *C. elegans* [24]. Therefore, lipofuscin levels in *C. elegans* were measured using fluorescence microscopy. *C. elegans* treated with KB2764 had markedly lower lipofuscin levels compared to *C. elegans* treated with DMSO (Figure 8b).

Body bending in *C. elegans* decreases significantly with ageing and is widely used as an indicator to measure ageing [26]. Body bending of *C. elegans* was assessed at 0, 7 and 14 days after DMSO or KB2764 treatment. On Day 0, no significant differences were observed in body bending between the two groups (Figure 8c). However, on Days 7 and 14, a significant increase in body bending was observed in *C. elegans* treated with KB2764 compared to *C. elegans* treated with DMSO (Figure 8c).

## 4 Discussion

ENO1 is an enzyme that converts 2‐phosphoglycerate to phosphoenolpyruvate and has received much attention for its glycolytic function [27]. In addition, ENO1 interacts with various intracellular molecules and plays a crucial role in cell signalling pathways [28]. A recent study reported that the cellular localisation of ENO1 affects mitochondrial function [29]. After treatment with doxorubicin, a chemotherapy drug used to treat various cancers, the mitochondrial localisation of ENO1 was reduced, whereas the cytoplasmic localisation of ENO1 was increased [29]. The reduced mitochondrial localisation of ENO1 renders mitochondria more sensitive to Ca^2+^‐induced membrane permeabilisation, indicating severe mitochondrial dysfunction [29]. Addition of recombinant ENO1 to a Ca^2+^ overload model of isolated mitochondria prevented mitochondrial membrane permeabilisation, restoring mitochondrial function [29]. However, the underlying mechanisms of how ENO1 activity is regulated and how this regulated activity affects mitochondrial function are not well understood. In this study, we found that KB2764 strengthens the interaction between PKM and ENO1, ultimately enabling PKM to phosphorylate ENO1. This regulation by KB2764 was accompanied by improved MMP and mitochondrial binding efficiency, consequently facilitating mitochondrial ATP production through OXPHOS. Therefore, the increase in energy production by OXPHOS resulted in a reduced dependence on glycolysis, as evidenced by the decrease in ECAR values induced by KB2764. Furthermore, the role of KB2764‐mediated regulation of ENO1 on metabolic reprogramming was confirmed by the finding that KB2764 treatment functioned as a prerequisite for increased mitochondrial ATP generation. Therefore, our findings suggest a novel mechanism by which KB2764 modulates ENO1 phosphorylation by enhancing the interaction between PKM and ENO1, thereby increasing mitochondrial ATP production and inducing metabolic reprogramming.

Senescent cells are characterised by increased mitochondrial size due to the accumulation of dysfunctional mitochondria [30]. Dysfunctional mitochondria produce ROS as a byproduct of electron leak from ETC and are also targets of direct damage by ROS [31]. The vicious cycle between dysfunctional mitochondria and ROS causes ageing and age‐related diseases [31]. Thus, strategies to decrease mitochondrial ROS production have been proposed for the effective treatment of ageing and age‐related diseases. A recent study showed that increasing the efficiency of mitochondrial ATP generation during OXPHOS was effective in reducing mitochondrial ROS production [18]. Support for this finding was also evident in the observation that supplementation of coenzyme Q, which enhances mitochondrial ATP production by increasing electron transfer from Complex II to III, reduced mitochondrial ROS production [32]. In this study, we found that KB2764‐mediated regulation of ENO1 significantly reduced ROS levels through an increase in the rate of oxygen consumption leading to ATP synthesis compared to inducing proton leak. These findings were further supported by the finding that senescent cells transduced with lentivirus expressing shENO1 did not reduce ROS levels even when treated with KB2764. Extending the relevance of these observations, we found that the increase in mitochondrial mass was reduced by KB2764 treatment as a result of a compensatory response to increased ROS. Additionally, DSBs and telomere damage caused by excessive intracellular ROS were reduced by KB2764 treatment. Our study is the first to demonstrate that regulation of ENO1 by KB2764 improves mitochondrial ATP production efficiency and consequently reduces ROS levels. Reduced ROS levels were accompanied by improvements in senescent phenotypes, making KB2764 a potential therapeutic option for ageing and age‐related diseases.

Mitochondria function as energy factories that maintain a constant supply of ATP [33]. Mitochondrial dysfunction due to senescence reduces mitochondrial ATP production [15]. To compensate for reduced energy levels, senescent cells become more dependent on energy production through glycolysis [34]. These metabolic changes lead to an imbalance in energy metabolism and accelerate cellular decline [34]. Imbalance in energy metabolism is not only a phenomenon that occurs as senescence progresses, but is also a cause of senescence [11]. Therefore, research focusing on metabolic reprogramming to reverse metabolic imbalances will provide new strategies for the treatment of ageing and age‐related diseases. To the best of our knowledge, our study is the first to reprogram energy metabolism in senescent cells by activating mitochondrial function and reducing dependence on glycolysis through ENO1 regulation by KB2764. Based on our findings, we propose that regulation of ENO1 phosphorylation by KB2764 might be an important means of reversing senescence by inducing metabolic reprogramming.

Compounds effective in reversing senescence at the cellular level have also been applied to lifespan extension studies using *C. elegans* to further verify their efficacy. Resveratrol regulates the expression of senescence‐related genes and delays the senescence process [35]. Resveratrol, which has been shown to improve senescence at the cellular level, was observed to extend lifespan when applied to *C. elegans* [36]. Metformin is a commonly prescribed medication for the management of type 2 diabetes [37]. At the cellular level, metformin improves senescence by enhancing autophagy and improving nutrient sensing [38]. Metformin was also effective in extending the lifespan of *C. elegans* [39]. Therefore, studies on lifespan extension in *C. elegans* provide an important foundation for the development of prevention and treatment strategies for age‐related diseases. In this study, we observed that KB2764 treatment extended the lifespan of *C. elegans* . The lifespan‐extending effect of KB2764 was also supported by observations of reduced lipofuscin levels and increased physical activity. We propose that KB2764 therapy might be applied to treat ageing and age‐related diseases. However, we acknowledge that further research is needed to investigate whether the lifespan‐extending effects of KB2764 can also be applied to mammalian models such as mice. These results will offer an a priori foundation for KB2764‐based therapeutic approaches in ageing and age‐related diseases.

ENO1 acts primarily as an enzyme known for its glycolytic function, but is also involved in a variety of other physiological processes [13]. For example, beyond its metabolic role, ENO1 is expressed in numerous cell types and acts as a plasminogen receptor [40, 41]. ENO1–plasminogen interaction facilitates the conversion of plasminogen to plasmin, thereby aiding in extracellular matrix degradation and tissue remodelling [42]. The underlying mechanism of ENO1 in muscle regeneration and muscle repair highlights the role of ENO1 in mice [13]. This finding is evident from the observation that inhibition of ENO1–plasminogen interaction affects extracellular matrix deposition, impairing muscle repair and leading to persistent muscle degeneration in mice [43]. ENO1 has also been implicated in the onset/development of ageing and age‐related diseases. ENO1 expression is upregulated in age‐associated neurodegenerative disorders such as early‐onset Alzheimer's disease (AD) and AD [44]. In addition to ENO1 upregulation, oxidative modification of ENO1 induces mitochondrial defects and contributes to the progression of AD [44, 45]. Thus, ENO1 has been suggested as a potential therapeutic target for neurodegenerative diseases [44]. Extending the relevance of these findings, an increase in ENO1 expression was observed in rheumatoid arthritis (RA), which is an age‐related disease [44, 46]. In RA synovial tissue, glycolytic activity increases, which in turn increases ENO1 expression, creating an acidic microenvironment that worsens RA [47]. The role of ENO1 in ageing is further supported by the findings showing elevated ENO1 expression in the dystrophic muscle of Duchenne muscular dystrophy mice and in the injury‐induced regenerative muscle of wild‐type mice [48]. The altered ENO1 activity leads to muscle atrophy, which in turn leads to decreased muscle endurance and strength [48, 49]. In this study, we observed that ENO1 knockdown significantly reduced ROS levels in senescent cells. However, we did not investigate the effect of ENO1 knockdown on senescence improvement. Further studies on ENO1 knockdown will strengthen our finding that KB2764 modulates ENO1 phosphorylation by enhancing the interaction between PKM and ENO1, thereby inducing senescence amelioration.

In summary, we screened a library of oxazole/pyrazole analogues and identified KB2764 as a candidate for improving senescence. KB2764 is bound to PKM and ENO1 and has increased their interaction. The KB2764‐mediated increase in interaction enhanced the phosphorylation of ENO1 by PKM. This phosphorylation increased mitochondrial ATP production, reducing dependence on glycolysis. Metabolic reprogramming by KB2764 improved mitochondrial function and thereby improved senescent phenotypes. The restorative effects of KB2764 applied to lifespan extension in *C. elegans* , providing groundbreaking evidence for the clinical use of KB2764 against ageing and age‐related diseases. Taken together, our results reveal a novel mechanism by which senescence is regulated by metabolic reprogramming upon KB2764‐mediated fine‐tuning of ENO1 protein activity.

## Author Contributions

**Yun Haeng Lee:** writing – original draft, writing – review and editing, investigation. **Hyunwoong Lim:** writing – original draft, writing – review and editing, investigation. **Gyungmin Kim:** investigation. **Geonhee Jang:** investigation. **Myeong Uk Kuk:** investigation. **Ji Ho Park:** investigation. **Jee hee Yoon:** investigation. **Yoo Jin Lee:** investigation. **Duyeol Kim:** investigation. **Byeonghyeon So:** investigation. **Minseon Kim:** investigation. **Hyung Wook Kwon:** investigation. **Youngjoo Byun:** writing – review and editing. **Joon Tae Park:** writing – review and editing.

## Ethics Statement

All animal experiment procedures were approved by the Animal Ethics and Welfare Committee of Incheon National University (protocol number: 20230112001).

## Conflicts of Interest

The authors declare no conflicts of interest.

## Supporting information


**Data S1.** Supporting Information.
**Figure S1.** Synthesis of KB2764. Reagents and conditions: (i) t‐BuONO, CuCl_2_, acetonitrile, 80°C, 6 h; (ii) 4‐(trifluoromethyl)phenylboronic acid, Pd(PPh_3_)_4_, toluene, H_2_O, K_2_CO_3_, 90°C, 16 h; (iii) 3‐bromotoluene, Pd(OAc)_2_, P(*o*‐tol)_3_, Cs_2_CO_3_, toluene, 90°C, 16 h.
**Figure S2.** Synthesis of biotinylated KB2764. Reagents and conditions: (i) 1 M NaOH, EtOH, r.t, 6 h; (ii) *N*‐(tert‐butoxycarbonyl)‐1,6‐diaminohexane, HOBt, EDC‐HCl, DMF, r.t., 6 h; (iii) TFA, DCM, r.t, 3 h; (iv) Biotin, HOBt, EDC‐HCl, DMF, r.t, 6 h.
**Figure S3.** NMR data of KB2764 and biotinylated KB2764.
**Figure S4.** HRMS data of KB2764 and biotinylated KB2764.
**Figure S5.** HPLC data of KB2764 and biotinylated KB2764.
**Figure S6.** KB2764 binds more strongly to ENO1 protein than to PKM. (a and b) Microscale thermophoresis (MST) analysis was conducted to quantitatively evaluate the binding affinity. Data obtained was plotted with concentration on *X*‐axis and Fnorm on *Y*‐axis. Then, the dissociation constant (*K*_d_) was calculated. Fnorm value is calculated by dividing F_1_ by F_0_. F_1_ is the fluorescence value measured in the heated state, and F_0_ corresponds to the fluorescence value measured in the cold state before turning on the IR laser. The dissociation constant (*K*_d_) of PKM was 9.32 μM, indicating a weak interaction between PKM and KB2764. The *K*_d_ of ENO1 was 40.09 nM, indicating a strong interaction between ENO1 and KB2764.
**Figure S7.** Ser 254 of ENO1 plays an important role in the binding between KB2764 and ENO1 protein. (a) In silico binding mode of the KB2764 in ENO1 (PDB ID: 3B97). Black and green dotted lines indicate hydrogen‐bonding and π–cation interaction, respectively. Docking studies KB2764 with ENO1 showed that the best‐docked pose was surrounded by Arg 253 and Ser 254. (b) Microscale thermophoresis (MST) analysis was conducted to quantitatively evaluate the binding affinity between KB2764 and ENO1 (S254A) protein. Data obtained was plotted with concentration on *X*‐axis and Fnorm on *Y*‐axis. Then, the dissociation constant (*K*_d_) was calculated. Fnorm value is calculated by dividing F_1_ by F_0_. F_1_ is the fluorescence value measured in the heated state, and F_0_ corresponds to the fluorescence value measured in the cold state before turning on the IR laser. *K*_d_ between KB2764 and ENO1 (S254A) protein was 0.24 mM.
**Figure S8.** (a) The utilisation of multiple sequence alignment has revealed the presence of a conserved motif in ENO1 across various species (human, ponab [ *Pongo abelii* ], mouse and Bovin [Bovines]). The utilisation of a kinase prediction tool allowed for the anticipation of a potential phosphorylation motif in ENO1 (http://www.cbs.dtu.dk/services/NetPhos/). Potential phosphorylation motifs were predicted to be serines (Ser) 254, 272 and 353, which are conserved across species. (b) Ser353 in ENO1 is identified as the potential phosphorylation motif. Cells were transfected with pCMV‐Myc‐ENO1 (WT), pCMV‐Myc‐ENO1 (S254A), pCMV‐Myc‐ENO1 (S272A) or pCMV‐Myc‐ENO1 (S353A). Immunoprecipitation was performed on cell lysates using an antibody against Myc‐tag. ENO1 (WT), ENO1 (S254A), ENO1 (S272A) or ENO1 (S353A) phosphorylation was detected by phospho‐Ser antibody. Phosphorylation of ENO1 (S254A) and ENO1 (S272A) was not reduced compared to ENO1 (WT) (blue‐boxed areas). Phosphorylation of ENO1 (S353A) was markedly compared to ENO1 (WT) (red boxed areas).
**Figure S9.** The effect of biotinylation on the function of KB2764. Flow cytometric analysis of reactive oxygen species (ROS) using DHR123. **p < 0.01, Student’s *t*‐test. Mean ± SD, *n* = 3.
**Table S1.** List of 149 proteins that bind only to biotinylated KB2764 and not to biotin.
**Table S2.** List of 137 proteins that bind only to biotin and not to biotinylated KB2764.
**Table S3.** List of 48 proteins that bind to both biotinylated KB2764 and biotin.

## Data Availability

The data that support the findings of this study are available from the corresponding author upon reasonable request.

## References

[1] L. Hayflick , “The Limited In Vitro Lifetime of Human Diploid Cell Strains,” Experimental Cell Research 37, no. 3 (1965): 614–636. 14315085 10.1016/0014-4827(65)90211-9

[2] D. Boffoli , S. C. Scacco , R. Vergari , G. Solarino , G. Santacroce , and S. Papa , “Decline With Age of the Respiratory Chain Activity in Human Skeletal Muscle,” Biochimica et Biophysica Acta (BBA) – Molecular Basis of Disease 1226, no. 1 (1994): 73–82. 8155742 10.1016/0925-4439(94)90061-2

[3] D. B. Zorov , M. Juhaszova , and S. J. Sollott , “Mitochondrial Reactive Oxygen Species (ROS) and ROS‐Induced ROS Release,” Physiological Reviews 94, no. 3 (2014): 909–950. 24987008 10.1152/physrev.00026.2013 PMC4101632

[4] A. H. Bittles and N. Harper , “Increased Glycolysis in Ageing Cultured Human Diploid Fibroblasts,” Bioscience Reports 4, no. 9 (1984): 751–756. 6509159 10.1007/BF01128816

[5] S. Din , M. H. Konstandin , B. Johnson , et al., “Metabolic Dysfunction Consistent With Premature Aging Results From Deletion of Pim Kinases,” Circulation Research 115, no. 3 (2014): 376–387. 24916111 10.1161/CIRCRESAHA.115.304441 PMC4254755

[6] S. Kakkar and B. Narasimhan , “A Comprehensive Review on Biological Activities of Oxazole Derivatives,” BMC Chemistry 13, no. 1 (2019): 16. 31384765 10.1186/s13065-019-0531-9 PMC6661760

[7] Y. H. Lee , D. Choi , G. Jang , et al., “Targeting Regulation of ATP Synthase 5 Alpha/Beta Dimerization Alleviates Senescence,” Aging (Albany NY) 14, no. 2 (2022): 678–707. 35093936 10.18632/aging.203858 PMC8833107

[8] T. P. Kilpeläinen , H. T. Pätsi , R. Svarcbahs , et al., “Nonpeptidic Oxazole‐Based Prolyl Oligopeptidase Ligands With Disease‐Modifying Effects on α‐Synuclein Mouse Models of Parkinson's Disease,” Journal of Medicinal Chemistry 66, no. 11 (2023): 7475–7496. 37248563 10.1021/acs.jmedchem.3c00235 PMC10258805

[9] P. Samakkarnthai , D. Saul , L. Zhang , et al., “In Vitro and In Vivo Effects of Zoledronic Acid on Senescence and Senescence‐Associated Secretory Phenotype Markers,” Aging (Albany NY) 15, no. 9 (2023): 3331–3355. 37154858 10.18632/aging.204701 PMC10449299

[10] V. Spirin , A. Shpunt , J. Seebacher , et al., “Assigning Spectrum‐Specific P‐Values to Protein Identifications by Mass Spectrometry,” Bioinformatics (Oxford, England) 27, no. 8 (2011): 1128–1134. 21349864 10.1093/bioinformatics/btr089 PMC3072553

[11] Y. H. Lee , J. Y. Park , H. Lee , et al., “Targeting Mitochondrial Metabolism as a Strategy to Treat Senescence,” Cells 10, no. 11 (2021): 3003. 34831224 10.3390/cells10113003 PMC8616445

[12] B. Park , J. Y. Kim , O. F. Riffey , et al., “Pyruvate Kinase M1 Regulates Butyrate Metabolism in Cancerous Colonocytes,” Scientific Reports 12, no. 1 (2022): 8771. 35610475 10.1038/s41598-022-12827-9 PMC9130307

[13] H. Ji , J. Wang , J. Guo , et al., “Progress in the Biological Function of Alpha‐Enolase,” Animal Nutrition 2, no. 1 (2016): 12–17. 29767008 10.1016/j.aninu.2016.02.005 PMC5941012

[14] C. J. Miller and B. E. Turk , “Homing in: Mechanisms of Substrate Targeting by Protein Kinases,” Trends in Biochemical Sciences 43, no. 5 (2018): 380–394. 29544874 10.1016/j.tibs.2018.02.009 PMC5923429

[15] S. Miwa , S. Kashyap , E. Chini , and T. von Zglinicki , “Mitochondrial Dysfunction in Cell Senescence and Aging,” Journal of Clinical Investigation 132, no. 13 (2022): e158447. 35775483 10.1172/JCI158447 PMC9246372

[16] R. M. Mayers , R. J. Butlin , E. Kilgour , et al., “AZD7545, a Novel Inhibitor of Pyruvate Dehydrogenase Kinase 2 (PDHK2), Activates Pyruvate Dehydrogenase In Vivo and Improves Blood Glucose Control in Obese (Fa/Fa) Zucker Rats,” Biochemical Society Transactions 31, no. Pt 6 (2003): 1165–1167. 14641018 10.1042/bst0311165

[17] L. D. Zorova , V. A. Popkov , E. Y. Plotnikov , et al., “Mitochondrial Membrane Potential,” Analytical Biochemistry 552 (2018): 50–59. 28711444 10.1016/j.ab.2017.07.009 PMC5792320

[18] S. Ghosh , R. Lertwattanarak , N. Lefort , et al., “Reduction in Reactive Oxygen Species Production by Mitochondria From Elderly Subjects With Normal and Impaired Glucose Tolerance,” Diabetes 60, no. 8 (2011): 2051–2060. 21677280 10.2337/db11-0121 PMC3142073

[19] B. Westermann , “Bioenergetic Role of Mitochondrial Fusion and Fission,” Biochimica et Biophysica Acta (BBA) – Bioenergetics 1817, no. 10 (2012): 1833–1838. 22409868 10.1016/j.bbabio.2012.02.033

[20] M. A. Petr , T. Tulika , L. M. Carmona‐Marin , and M. Scheibye‐Knudsen , “Protecting the Aging Genome,” Trends in Cell Biology 30, no. 2 (2020): 117–132. 31917080 10.1016/j.tcb.2019.12.001

[21] D. R. Green , L. Galluzzi , and G. Kroemer , “Mitochondria and the Autophagy‐Inflammation‐Cell Death Axis in Organismal Aging,” Science 333, no. 6046 (2011): 1109–1112. 21868666 10.1126/science.1201940 PMC3405151

[22] H. Jiang , Z. Ju , and K. L. Rudolph , “Telomere Shortening and Ageing,” Zeitschrift für Gerontologie und Geriatrie 40, no. 5 (2007): 314–324. 17943234 10.1007/s00391-007-0480-0

[23] L. Zhang , L. E. Pitcher , M. J. Yousefzadeh , L. J. Niedernhofer , P. D. Robbins , and Y. Zhu , “Cellular Senescence: A Key Therapeutic Target in Aging and Diseases,” Journal of Clinical Investigation 132, no. 15 (2022): e158450. 35912854 10.1172/JCI158450 PMC9337830

[24] Z. Pincus , T. C. Mazer , and F. J. Slack , “Autofluorescence as a Measure of Senescence in *C. elegans*: Look to Red, Not Blue or Green,” Aging (Albany NY) 8, no. 5 (2016): 889–898. 27070172 10.18632/aging.100936 PMC4931842

[25] M. Uno and E. Nishida , “Lifespan‐Regulating Genes in *C. elegans* ,” npj Aging and Mechanisms of Disease 2, no. 1 (2016): 16010. 28721266 10.1038/npjamd.2016.10 PMC5514992

[26] H. Zhang and W. Chen , “Automated Recognition and Analysis of Body Bending Behavior in *C. elegans* ,” BMC Bioinformatics 24, no. 1 (2023): 175. 37118676 10.1186/s12859-023-05307-y PMC10148436

[27] G. Qiao , A. Wu , X. Chen , Y. Tian , and X. Lin , “Enolase 1, a Moonlighting Protein, as a Potential Target for Cancer Treatment,” International Journal of Biological Sciences 17, no. 14 (2021): 3981–3992. 34671213 10.7150/ijbs.63556 PMC8495383

[28] N. Dephoure , C. Zhou , J. Villén , et al., “A Quantitative Atlas of Mitotic Phosphorylation,” Proceedings of the National Academy of Sciences of the United States of America 105, no. 31 (2008): 10762–10767. 18669648 10.1073/pnas.0805139105 PMC2504835

[29] S. Gao , H. Li , Y. Cai , et al., “Mitochondrial Binding of α‐Enolase Stabilizes Mitochondrial Membrane: Its Role in Doxorubicin‐Induced Cardiomyocyte Apoptosis,” Archives of Biochemistry and Biophysics 542 (2014): 46–55. 24361255 10.1016/j.abb.2013.12.008

[30] C. Giorgi , S. Marchi , I. C. M. Simoes , et al., “Mitochondria and Reactive Oxygen Species in Aging and Age‐Related Diseases,” International Review of Cell and Molecular Biology 340 (2018): 209–344. 30072092 10.1016/bs.ircmb.2018.05.006 PMC8127332

[31] C. Guo , L. Sun , X. Chen , and D. Zhang , “Oxidative Stress, Mitochondrial Damage and Neurodegenerative Diseases,” Neural Regeneration Research 8, no. 21 (2013): 2003–2014. 25206509 10.3969/j.issn.1673-5374.2013.21.009 PMC4145906

[32] G. Tian , J. Sawashita , H. Kubo , et al., “Ubiquinol‐10 Supplementation Activates Mitochondria Functions to Decelerate Senescence in Senescence‐Accelerated Mice,” Antioxidants & Redox Signaling 20, no. 16 (2014): 2606–2620. 24124769 10.1089/ars.2013.5406 PMC4025630

[33] Y. Ohya , N. Umemoto , I. Tanida , A. Ohta , H. Iida , and Y. Anraku , “Calcium‐Sensitive Cls Mutants of *Saccharomyces cerevisiae* Showing a Pet‐Phenotype Are Ascribable to Defects of Vacuolar Membrane H(+)‐ATPase Activity,” Journal of Biological Chemistry 266, no. 21 (1991): 13971–13977. 1830311

[34] C. D. Wiley and J. Campisi , “From Ancient Pathways to Aging Cells‐Connecting Metabolism and Cellular Senescence,” Cell Metabolism 23, no. 6 (2016): 1013–1021. 27304503 10.1016/j.cmet.2016.05.010 PMC4911819

[35] L. Matos , A. M. Gouveia , and H. Almeida , “Resveratrol Attenuates Copper‐Induced Senescence by Improving Cellular Proteostasis,” Oxidative Medicine and Cellular Longevity 2017 (2017): 3793817. 28280523 10.1155/2017/3793817 PMC5322428

[36] K. S. Bhullar and B. P. Hubbard , “Lifespan and Healthspan Extension by Resveratrol,” Biochimica et Biophysica Acta (BBA) – Molecular Basis of Disease 1852, no. 6 (2015): 1209–1218. 25640851 10.1016/j.bbadis.2015.01.012

[37] S. A. Hawley , A. E. Gadalla , G. S. Olsen , and D. G. Hardie , “The Antidiabetic Drug Metformin Activates the AMP‐Activated Protein Kinase Cascade via an Adenine Nucleotide‐Independent Mechanism,” Diabetes 51, no. 8 (2002): 2420–2425. 12145153 10.2337/diabetes.51.8.2420

[38] A. S. Kulkarni , S. Gubbi , and N. Barzilai , “Benefits of Metformin in Attenuating the Hallmarks of Aging,” Cell Metabolism 32, no. 1 (2020): 15–30. 32333835 10.1016/j.cmet.2020.04.001 PMC7347426

[39] J. Chen , Y. Ou , Y. Li , S. Hu , L.‐W. Shao , and Y. Liu , “Metformin Extends *C. elegans* Lifespan Through Lysosomal Pathway,” eLife 6 (2017): e31268. 29027899 10.7554/eLife.31268 PMC5685485

[40] R. Lopez‐Alemany , P. Correc , L. Camoin , and P. Burtin , “Purification of the Plasmin Receptor From Human Carcinoma Cells and Comparison to Alpha‐Enolase,” Thrombosis Research 75, no. 4 (1994): 371–381. 7997975 10.1016/0049-3848(94)90252-6

[41] L. A. Miles , C. M. Dahlberg , J. Plescia , J. Felez , K. Kato , and E. F. Plow , “Role of Cell‐Surface Lysines in Plasminogen Binding to Cells: Identification of Alpha‐Enolase as a Candidate Plasminogen Receptor,” Biochemistry 30, no. 6 (1991): 1682–1691. 1847072 10.1021/bi00220a034

[42] M. Wygrecka , L. M. Marsh , R. E. Morty , et al., “Enolase‐1 Promotes Plasminogen‐Mediated Recruitment of Monocytes to the Acutely Inflamed Lung,” Blood 113, no. 22 (2009): 5588–5598. 19182206 10.1182/blood-2008-08-170837

[43] A. Díaz‐Ramos , A. Roig‐Borrellas , A. García‐Melero , and R. López‐Alemany , “α‐Enolase, a Multifunctional Protein: Its Role on Pathophysiological Situations,” Journal of Biomedicine & Biotechnology 2012 (2012): 156795. 23118496 10.1155/2012/156795 PMC3479624

[44] D. A. Butterfield and M. L. Lange , “Multifunctional Roles of Enolase in Alzheimer's Disease Brain: Beyond Altered Glucose Metabolism,” Journal of Neurochemistry 111, no. 4 (2009): 915–933. 19780894 10.1111/j.1471-4159.2009.06397.x PMC4454338

[45] A. Castegna , M. Aksenov , V. Thongboonkerd , et al., “Proteomic Identification of Oxidatively Modified Proteins in Alzheimer's Disease Brain. Part II: Dihydropyrimidinase‐Related Protein 2, Alpha‐Enolase and Heat Shock Cognate 71,” Journal of Neurochemistry 82, no. 6 (2002): 1524–1532. 12354300 10.1046/j.1471-4159.2002.01103.x

[46] A. Kinloch , V. Tatzer , R. Wait , et al., “Identification of Citrullinated Alpha‐Enolase as a Candidate Autoantigen in Rheumatoid Arthritis,” Arthritis Research & Therapy 7, no. 6 (2005): R1421–R1429. 16277695 10.1186/ar1845 PMC1297593

[47] X. Chang and C. Wei , “Glycolysis and Rheumatoid Arthritis,” International Journal of Rheumatic Diseases 14, no. 3 (2011): 217–222. 21816017 10.1111/j.1756-185X.2011.01598.x

[48] R. Lopez‐Alemany , M. Suelves , A. Diaz‐Ramos , B. Vidal , and P. Munoz‐Canoves , “Alpha‐Enolase Plasminogen Receptor in Myogenesis,” Frontiers in Bioscience 10 (2005): 30–36. 15574344 10.2741/1503

[49] R. Ramadasan‐Nair , N. Gayathri , S. Mishra , et al., “Mitochondrial Alterations and Oxidative Stress in an Acute Transient Mouse Model of Muscle Degeneration: Implications for Muscular Dystrophy and Related Muscle Pathologies,” Journal of Biological Chemistry 289, no. 1 (2014): 485–509. 24220031 10.1074/jbc.M113.493270 PMC3879571

