# Supplementary material for: Elucidating the Role and Mechanism of Alpha‐Enolase in Senescent Amelioration via Metabolic Reprogramming
Source: Cell Prolif. 2025 Apr 27;58(10):e70049. doi: 10.1111/cpr.70049 (PMC12508687; doi:10.1111/cpr.70049)
Supplement: Supplementary file 1 — Data S1. Supporting Information. Figure S1. Synthesis of KB2764. Reagents and conditions: (i) t‐BuONO, CuCl2, acetonitrile, 80°C, 6 h; (ii) 4‐(trifluoromethyl)phenylboronic acid, Pd(PPh3)4, toluene, H2O, K2CO3, 90°C, 16 h; (iii) 3‐bromotoluene, Pd(OAc)2, P(o‐tol)3, Cs2CO3, toluene, 90°C, 16 h. Figure S2. Synthesis of biotinylated KB2764. Reagents and conditions: (i) 1 M NaOH, EtOH, r.t, 6 h; (ii) N‐(tert‐butoxycarbonyl)‐1,6‐diaminohexane, HOBt, EDC‐HCl, DMF, r.t., 6 h; (iii) TFA, DCM, r.t, 3 h; (iv) Biotin, HOBt, EDC‐HCl, DMF, r.t, 6 h. Figure S3. NMR data of KB2764 and biotinylated KB2764. Figure S4. HRMS data of KB2764 and biotinylated KB2764. Figure S5. HPLC data of KB2764 and biotinylated KB2764. Figure S6. KB2764 binds more strongly to ENO1 protein than to PKM. (a and b) Microscale thermophoresis (MST) analysis was conducted to quantitatively evaluate the binding affinity. Data obtained was plotted with concentration on X‐axis and Fnorm on Y‐axis. Then, the dissociation constant (K d) was calculated. Fnorm value is calculated by dividing F1 by F0. F1 is the fluorescence value measured in the heated state, and F0 corresponds to the fluorescence value measured in the cold state before turning on the IR laser. The dissociation constant (K d) of PKM was 9.32 μM, indicating a weak interaction between PKM and KB2764. The K d of ENO1 was 40.09 nM, indicating a strong interaction between ENO1 and KB2764. Figure S7. Ser 254 of ENO1 plays an important role in the binding between KB2764 and ENO1 protein. (a) In silico binding mode of the KB2764 in ENO1 (PDB ID: 3B97). Black and green dotted lines indicate hydrogen‐bonding and π–cation interaction, respectively. Docking studies KB2764 with ENO1 showed that the best‐docked pose was surrounded by Arg 253 and Ser 254. (b) Microscale thermophoresis (MST) analysis was conducted to quantitatively evaluate the binding affinity between KB2764 and ENO1 (S254A) protein. Data obtained was plotted with concentration on X‐axis and Fnor [file CPR-58-e70049-s001.docx]

**Supplementary information**

**Experimental procedures**

**Synthesis of KB2764 and biotinylated KB2764**

Synthetic methods, experimental procedures, and analytical data of KB2764 and biotinylated KB2764 (Supplementary Fig. 1–supplementary Fig. 5) are described.

**Cell culture**

Human diploid fibroblasts (HDF; PCS–201–010; ATCC, Manassas, VA, USA), human embryonic kidney cells (HEK293T; CRL–11268; ATCC) and Hela cells (A1100001; Thermo Fisher Scientific, Waltham, MA, USA) were used in this study. Cells were cultured in Dulbecco’s modified Eagle’s medium containing 25 mM glucose supplemented with 10% fetal bovine serum (SH30919.03; Hyclone, Waltham, MA, USA) and 100 U/ml penicillin & 100 μg/ml streptomycin (SV30079.01; Hyclone). Cell culture was carried out in the same way as in previous studies ^1^. When the population doubling time of the senescent fibroblast was over 14 days. Cell viability was assessed using Cedex HiRes Analyzer (05650216001; Roche, Basel, Switzerland).

**Compound library screening**

Senescent cells were grown in 96–well plates at a density of 2,000 cells per well. Components from the library were diluted to a final concentration of 8 µM and added to the wells every 4 days. 12 days after drug treatment, cell proliferation was measurement. The cell proliferation measurement was conducted by the method of the previous study^1^.

**Ion trap mass spectrometry**

Senescent HDFs were treated for 12 days with 8 µM biotinylated KB2764 or 8 µM biotin. Immunoprecipitation and ion trap mass spectrometry were performed as previously described^1^.

**Plasmid construction**

pCMV–Myc–PKM was constructed by inserting cDNA encoding pyruvate kinase M (GenBank accession: NM_001206796.3) into the pCMV–Myc vector (631604; Clontech, Mountain View, CA, USA). Following primers were used to construct pCMV–Myc–PKM: 5'-AGTCCAGTGTGGTGGAATGTCGAAGCCCCATAGTGA-3' (forward), 5'- GCCACTGTGCTGGATTCACGGCACAGGAACAACA-3' (reverse). pCMV–Myc–ENO1 was constructed by inserting cDNA encoding human alpha–enolase (GenBank accession: NM_001428.5) into the pCMV–Myc vector. Following primers were used to construct pCMV–Myc–ENO1: 5'-AGTCCAGTGTGGTGGAATGTCTATTCTCAAGATCCATG-3' (forward), 5'-GCCACTGTGCTGGATTTACTTGGCCAAGGGGTTTC-3' (reverse). pCMV–Myc–ENO1 (S254A), pCMV–Myc–ENO1 (S272A) and pCMV–Myc–ENO1 (S353A) were prepared by mutagenesis from pCMV–Myc–ENO1 using a HiFi DNA Assembly Master Mix (E2621L; New England Biolabs, Ipswich, MA, USA). Following primers were used to construct pCMV–Myc–ENO1 (S254A): 5'-GAGTTCTTCAGGTCTGGGAAGTATGACCTGGACTT-3' (forward), 5'-AGACCTGAAGAACTCGGAGGCCGCTACGTCCATGC-3' (reverse). Following primers were used to construct pCMV–Myc–ENO1 (S272A): 5'-AGCAGGTACATCTCGCCTGACCAGCTGGCTGACCT-3' (forward), 5'-CGAGATGTACCTGCTGGGGTCATCGGGAGACTTGA-3' (reverse). Following primers were used to construct pCMV–Myc–ENO1 (S353A): 5'-TCCGTGACCGAGTCTCTTCAGGCGTGCAAGCTGGC-3' (forward), 5'-AGACTCGGTCACGGAGCCAATCTGGTTGACTTTGA-3' (reverse). pCMV–His tag–Myc–PKM was prepared by mutagenesis from pCMV–Myc–PKM using a HiFi DNA Assembly Master Mix. Following primers were used to construct pCMV–His tag–Myc–PKM: 5'-CATCATCACCATCACCATGGATCCTCGGATCCACTAGT-3' (forward), 5'-ATGGTGATGGTGATGATGCAGGTCCTCCTCGGAGATCA-3' (reverse). pCMV–His tag–Myc–ENO1 was prepared by mutagenesis from pCMV–Myc–ENO1 using a HiFi DNA Assembly Master Mix. Following primers were used to construct pCMV–His tag–Myc–ENO1: 5'-CATCATCACCATCACCATGGATCCTCGGATCCACTAGT-3' (forward), 5'-ATGGTGATGGTGATGATGCAGGTCCTCCTCGGAGATCA-3' (reverse). pLKO.1–shENO1, pLKO.1–shPKM was constructed by inserting target gene into the pLKO.1 plasmid. Following primers were used to construct pLKO.1–shENO1: 5'-CCGGCCACTGTTGAGGTTGATCTCTCTCGAGAGAGATCAACCTCAACAGTGGTTTTTG-3' (forward), 5'-AATTCAAAAACCACTGTTGAGGTTGATCTCTCTCGAGAGAGATCAACCTCAACAGTGG-3' (reverse). pLKO.1–shPKM: 5'- CCGGCCACTTGCAATTATTTGAGGACTCGAGTCCTCAAATAATTGCAAGTGGTTTTTG-3' (forward), 5'- AATTCAAAAACCACTTGCAATTATTTGAGGACTCGAGTCCTCAAATAATTGCAAGTGG-3' (reverse).

**Immunoprecipitation**

pCMV–Myc–PKM, pCMV–Myc–ENO1, pCMV–Myc–ENO1 (S254A), pCMV–Myc–ENO1 (S272A) and pCMV–Myc–ENO1 (S353A) plasmids were transfected into HEK293T cells. Transfected cells were selected with 1 μM puromycin (BML–GR312–0050; Enzo Lifescinece) for 14 days. During selection, the medium was changed every 2 days. Then, immunprecipitation was performed using anti–Myc tag antibody (2276s; cell signaling). The following immunoprecipitation assay was performed as described^2^. Proteins were separated on a 10% SDS–PAGE gel and analyzed by immunoblotting.

**Western blot analysis**

Western blotting was performed as previously described^3^. Proteins were detected with the SuperSignal™ West Pico chemiluminescence solution (34577; Thermo Fisher Scientific, Waltham, MA, USA) using a Chemidoc XRS+ system (1708265; BIO-RAD; Hercules, CA, USA). The primary antibodies used in this study included anti–Myc tag antibody (2276s; 1:200 dilution; cell signaling), anti–phospho-Ser antibody (2851s; 1:1,000 dilution; cell signaling), anti–enolase–1 (3810T; 1: 1,000 dilution; Cell Signaling Technology), and HRP–conjugated β–actin (sc47778; 1:1,000 dilution; Santa Cruz Biotechnology, Dallas, TX, USA). The secondary antibodies used in this study included HRP-conjugated anti-mouse IgG (sc-2302; 1:1,000 dilution; Santa Cruz Biotechnology) and HRP–conjugated anti–rabbit IgG (sc–2004; 1:2,000 dilution; Santa Cruz Biotechnology).

**Microscale thermophoresis (MST) assay**

6×His–tagged PKM protein and 6×His–tagged ENO1 protein were purified using the Capturem™ His–Tagged Purification Maxiprep Kit (635713; Takara, Mountain View, CA, USA). 200 nM of 6×His–tagged PKM protein and 6×His–tagged ENO1 protein were labeled using Monolith His–Tag Labeling Kit RED–tris–NTA 2nd Generation (MO–L018; Nano Temper Technologies, München, Germany) and 0.4 mM of KB2764 was used. MST assay was performed as described previously^1^.

***In silico* docking studies**

Ligand preparation and optimization: KB–2764 was created in 2D and 3D structures by ChemDrawUltra (version 12.0.2, CambridgeSoft) and Maestro (version 13.4.134, MMshare Version 6.0.134, Schrodinger), respectively. In the Maestro builder panel, a LigPrep module was used to add hydrogen atoms, remove salts, and ionize at pH (7 ± 2) to create a 3D structure of the ligand. Protein Preparation: An ENO1 structure (PDB ID: 3B97) was obtained from the protein data bank (https://www.rcsb.org ). Protein structure was prepared using the Protein Preparation Workflow on the Maestro panel. During the preparation of the protein, the bond order was specified, and hydrogen atoms were added. The water molecules were removed. Receptor grid boxes were generated using “Glide's Receptor Grid Generation” module. The docking study of KB–2764 with ENO1 was conducted using the Standard Precision ligand docking protocol on the Maestro panel.

**Seahorse analysis**

The Seahorse XFe96 analyzer (Aglient Technolongy, Santa Clara, CA, USA) was used according to the manufacturer’s protocol. Briefly, 1×10^4^ cells were distributed into each well of an XFe96 cell–culture plate (101085–004; Aglient Technolongy) and then cultured in a 5% CO_2_ incubator at a temperature of 37 ℃ for 16 h. Next, the medium was replaced by seahorse XF DMEM pH 7.4 (103680–100; Aglient Technolongy). The extracellular acidification rate (ECAR) was measured using a Seahorse XF Glycolysis Stress Test kit (103020–100; Aglient Technolongy). ECAR was reported in mpH/min. The sensor cartridges were used in the Seahorse XFe96 Extracellular Flux Assay Kit (102416–100; Agilent Technology). Seahorse analysis was performed as previously described ^4^. To induce metabolic changes in senescent cells, AZD7545 (S7517; Selleckchem, Houston, TX, USA) was used at a concentration of 20 μM.

**Measurement of mitochondrial membrane potential (MMP)**

For measurement of the mitochondrial membrane potential, the cells were incubated with 0.6 µg/ml JC–10 (ab112134; abcam, Waltham, MA, USA) for 30 min at 37 ℃. After staining, cells were prepared for FACS analysis as previously study^1^.

**Measurement of ROS, mitochondrial mass and lipofuscin**

For quantification of mitochondrial ROS, the cells were incubated in medium containing 5 µM MitoSOX (M36008; Life Technologies, Carlsbad, CA, USA) for 30 min at 37 ℃. For quantification of mitochondrial mass, the cells were incubated in medium containing 50 nM MitoTracker green (M7514; Life Technologies) for 30 min at 37 ℃. For measurement of the lipofuscin in HDFs, cells without staining. Cells were prepared for FACS analysis as previously study^1^.

**Lenti**–**viral production and infection**

HEK 293T cells were transfected with 8 μg shRNA plasmid (pLK.O1–shCTRL and pLK.O1–shENO1), 4 μg PAX2 plasmid, and 4 μg VSV.G plasmid using Lipofectamine 2000 (11668019; Invitrogen). Viral supernatant was harvested 24 h after transfection. Polybrene (TR–1003–G; 8 μg/ml; Millipore, Burlington, Middlesex County, MA, USA) was added into viral supernatant. Virus infection was performed senescent cells as previously described^4^.

**Neutral comet assay**

After preparing the trypsinized cells at a concentration of 1×10^5^ cells/ml, cells are washed with PBS. Mix with 50 μl of cell and 500 μl of melted LMAgrose. After spreaing 75 μl of the mixture on a comet slide, block the light and incubate at 4 ℃ for 10 min. Dip the hardened comet slide in lysis solution and incubate at 4 ℃ for 1 h. Transfer the comet slide from lysis solution to 1× TBE buffer and incubate at 4 ℃ for 30 min. The comet slide is subjected to electrophoresis under the condition of 35V, 45min. Incubate the electrophoresed comet slide in 70% ethanol at RT for 5 min. Dry the fixed comet slide at 37 ℃ for 15 min. 50 μl per well of SYBR Green staining solution (1:1000 dilution at distilled water (D.W)) was observed by FITC microscopy (484 nm/521 nm).

**Measurement of telomere length**

Telomere length was performed by genomic DNA quantitative PCR (qPCR) analysis as described previously^1^. qPCR was performed using the following primer: 5'- CGGTTTGTTTGGGTTTGGGTTTGGGTTTGGGTTTGGGTT-3' (*Telomere*-forward) 5'- GGCTTGCCTTACCCTTACCCTTACCCTTACCCTTACCCT-3' (*Telomere*-reverse), 5'- CAGCAAGTGGGAAGGTGTAATCC-3' (*36B4*-forward) 5'- CCCATTCTATCATCAACGGGTACAA -3' (*36B4*-reverse).

**Reagent and medium preparation for *C. elegans* culture**

M9 buffer contained 5.8 g Na_2_HPO_4_∙7H_2_0, 3.0 g KH_2_PO_4_, 5.0 g NaCl, 0.25 g MgSO_4_∙7H_2_0 and 1L D.W M9 buffer was filtered through a 0.22 μm filter. 20% alkaline hypochlorite solution was prepared by mixing 3.75 ml NaOH (1M), 3.0 ml Bleach and 8.25 ml D.W For nematode growth medium (NGM), 3 g NaCl, 2.5 g peptone, 17 g agar and 975 ml D.W were mixed and autoclaved. The bottle was cooled to 55 ℃, and 1 ml CaCl_2_ (1 M), 1 ml MgSO_4_ (1 M), 25 ml KPO_4_ (1 M, pH 6.0) and 1 ml cholesterol in ethanol (5 mg/ml) were mixed to form a plate. OP50 (#053114; EDVOTEK, Washington, DC, USA)–cultured NGM medium was prepared by seeding OP50 in NGM medium and then overnight in an incubator at 37 ℃.

***C. elegans* culture and synchronization**

*C. elegans* (WT) was seeded on OP50–cultured NGM medium and incubated at 22 ℃. After growing *C. elegans* for 2–3 days, the eggs and worms present on the plate were prepared with 3 ml M9 buffer. Sediment the eggs and worms for 10 min, then remove the supernatant. Gently invert for 5 min while completing 15 ml of 20% alkaline hypochlorite solution. After returning the centrifuge at 760 × *g*, 2 min, wash twice with M9 buffer. Finally, insert 7 ml of M9 buffer, shake gently and leave overnight. Next day, L1 stage *C. elegans* are grown on OP50–cultured NGM medium. OP50–cultured NGM medium was redeployed every 2 days.

***C. elegans* survival analysis**

Synchronized *C. elegans* were treated with 5–fluoro–2ʹ–deoxyuridine (FUdR, 50 μM) to prevent egg laying. Synchronized L1 stage *C. elegans* was incubated for 2–3 days at 22 ℃. Adult *C. elegans* were transferred to OP50–cultured NGM medium plates containing DMSO or KB2764 (100 μM). Survival assays were performed at 22 ℃*. elegans* in each group, and medium was changed every 2 days. The viability of *C. elegans* was observed under a microscope and checked daily. Survival analysis was performed using the Kaplan–Meier method.

**Measurement of *C. elegans* lipofuscin**

Lipofuscin detection was performed on day 10 of cultivation on OP50–cultured NGM medium plates containing either DMSO or KB2764 (100 μM). The presence of lipofuscin was observed using a X–Cite^®^ 120Q (Excelitas Technologies, Waltham, MA, USA) equipped with Zeiss AxioVert 200 (Zeiss, Oberkochen, Land Baden–Württemberg, Germany). Lipofuscin of *C. elegans* were observed at 450–490 nm.

**Measurement of *C. elegans* body bends**

Body bends measurement was performed on day 10 of cultivation on OP50–cultured NGM medium plates containing either DMSO or KB2764 (100 μM). *C. elegans* wriggling (based on round trips) was measured by microscopy based on 1 min.

**Statistical analyses**

Statistical analyses were performed using a standard statistical software package (SigmaPlot 12.5; Systat Software, San Jose, CA, USA). The student’s t–test and two–way ANOVA followed by Bonferroni’s posttest were used to determine whether differences were significant. A two–way ANOVA was performed for data analysis with column factors. The Kaplan–Maier method was tested in GraphPad Prism 7 software (GraphPad, San Diego, CA, USA). Significance between survival curves was assessed by the Kaplan–Meier method.

**Procedure for the synthesis of KB2764 and biotinylated KB2764**

All chemicals and solvents used in the reaction were purchased from Sigma–Aldrich, TCI, and Acros and were used without further purification. The reaction progress was monitored by thin–layer chromatography (TLC) on precoated silica gel plates with silica gel 60F_254_ (Merck; Darmstadt, Germany) and visualized by UV254 light and/or KMnO_4_ staining for detection purpose. Column chromatography was performed on a silica gel (silica gel 60; 230–400 mesh ASTM, Merck, Darmstadt, Germany). Nuclear magnetic resonance (NMR) spectra were recorded at room temperature on a Bruker UltraShield Plus (^1^H 600 MHz; ^13^C 150 MHz) spectrometer. All chemical shifts are reported in parts per million (ppm) from tetramethylsilane (δ = 0) and were measured relative to the solvent in which the sample was analyzed (CDCl_3_: δ 7.26 for ^1^H NMR, δ 77.0 for ^13^C NMR; MeOH– d_4_: δ 3.31 for ^1^H NMR, δ 49.0 for ^13^C NMR). The ^1^H NMR shift values are reported as chemical shift (δ), the corresponding integral, multiplicity (s = singlet, br = broad, d = doublet, t = triplet, q = quartet, m = multiplet, dd = doublet of doublets, td = triplet of doublets, qd = quartet of doublets), coupling constant (J in Hz), and assignments. High resolution mass spectra (HRMS) were recorded on an Agilent 6530 Accurate Mass Q–TOF LC/MS spectrometer.

 Supplementary Fig. 1. Synthesis of KB2764. *Reagents and Conditions*: (*i*) *t*–BuONO, CuCl_2_, acetonitrile, 80 ℃, 6 h; (*ii*) 4–(trifluoromethyl)phenylboronic acid, Pd(PPh_3_)_4_, toluene, H_2_O, K_2_CO_3_, 90 ℃, 16 h; (*iii*) 3–bromotoluene, Pd(OAc)_2_, P(*o*–tol)_3_, Cs_2_CO_3_, toluene, 90 ℃, 16 h.

*Ethyl 2–chlorooxazole–4–carboxylate* (2)

Ethyl 2–aminooxazole–4–carboxylate (5.00 g, 32.0 mmol) was added in portions to a stirred solution of *tert*–butyl nitrite (5.70 mL, 48.0 mmol, 1.5 eq), copper(Ⅱ) chloride (6.50 g, 48.0 mmol, 1.5 eq) in acetonitrile (150 mL) The reaction mixture was stirred at 80 °C for 6 h. The solution was cooled and partitioned between dichloromethane (170 mL), water (80 mL), and concentrated hydrochloric acid (8 mL). The organic layer was dried over MgSO_4_ and concentrated under reduced pressure. The residue was purified by column chromatography on a silica gel (Hexane/Et_2_O = 11:1 to 10:1, v/v) to provide compound 2 (3.09 g, 49%). R*_f_* 0.43 (Hexane/Et_2_O = 3:1 v/v). ^1^H NMR (600 MHz, CDCl_3_) *δ* 8.19 (s, 1H), 4.40 (q, *J* = 7.2 Hz, 2H), 1.39 (t, *J* = 7.2 Hz, 3H).

*Ethyl 2–(4–(trifluoromethyl)phenyl)oxazole–4–carboxylate* (3)

To a solution of 4–(trifluoromethyl)phenylboronic acid (1.26 g, 6.63 mmol, 1.5 eq), Pd(PPh_3_)_4_ (630 mg), 2 (776 mg, 4.42 mmol) in toluene (20 mL) was add K_2_CO_3_ in H_2_O (2 mL). The reaction mixture was stirred at 80 °C for 16 h. The solution was cooled and partitioned between ethyl acetate, water. The aqueous layer was further extracted with ethyl acetate (2 x 30 mL) and the combined organics washed with dried (MgSO_4_), and evaporated. The residue was purified by column chromatography on a silica gel (Hexane/Et_2_O = 9:1 to 6:1, v/v), and gave 3 (1.01 g, 80%). R*_f_* 0.55 (Hexane/Et_2_O = 1:1 v/v). ^1^H NMR (600 MHz, CDCl_3_) : *δ* 8.35 (s, 1H), 8.25 (d, *J* = 7.8 Hz, 2H), 7.75 (d, *J* = 7.8 Hz, 2H), 4.47 (q, *J* = 7.2 Hz, 2H), 1.44 (t, *J* = 7.2 Hz, 3H).

*Ethyl 5–(m–tolyl)–2–(4–(trifluoromethyl)phenyl)oxazole–4–carboxylate* (4)

To a solution of 3 (303 mg, 1.06mmol) were added 3–bromotoluene (154 μL, 1.27 mmol, 1.2 eq), cesium carbonate (518 mg, 1.6 mmol, 1.5 eq), tri(*o*–tolyl)phosphine (65 mg, 0.21 mmol, 0.2 eq), and palladium acetate (47.6 mg, 0.21 mmol, 0.2 eq) in toluene (20 mL). The reaction mixture was stirred under argon for 16 h at 90 °C. The solution was cooled and partitioned between ethyl acetate and water. The aqueous layer was further extracted with ethyl acetate (2 x 30 mL) and the combined organic layer was washed, dried (MgSO_4_) and evaporated. The residue was purified by column chromatography on a silica gel (Hexane/EtOAc = 9:1 to 6:1, v/v) to afford compound **4** (348 mg 88%), R_f_ 0.67 (Hexane/EtOAc = 2:1, v/v) , ^1^H NMR (600 MHz, CDCl_3_) δ 8.28 (d, *J* = 8.2 Hz, 2H), 7.90 (d, *J* = 7.9 Hz, 2H), 7.75 (d, *J* = 8.2 Hz, 2H), 7.40 (dd, *J* = 11.5, 4.2 Hz, 1H), 7.32 (d, *J* = 7.4 Hz, 1H), 4.46 (q, *J* = 7.1 Hz, 2H), 2.46 (s, 3H), 1.43 (t, *J* = 7.1 Hz, 3H). HRMS *m/z*: calcd for C_20_H_16_F_3_NO_3_ [M+H]^+^ 376.1116; found, 376.1158.

Supplementary Fig. 2 Synthesis of biotinylated KB2764. *Reagents and Conditions*: *(i)* 1 M NaOH, EtOH, r.t, 6 h; *(ii) N*–(*tert*–butoxycarbonyl)–1,6–diaminohexane, HOBt, EDC–HCl, DMF, r.t., 6 h; *(iii)* TFA, DCM, r.t, 3 h; *(iv)* Biotin, HOBt, EDC–HCl, DMF, r.t, 6 h.

*5–(m–Tolyl)–2–(4–(trifluoromethyl)phenyl)oxazole–4–carboxylic acid* (5)

To a solution of 4 (330 mg, 0.88 mmol) was added 1 N NaOH (2.64 mL, 2.64 mmol, 3 eq) in ethanol (20 mL). The reaction mixture was stirred for 6 h at room temperature. The solution was concentrated under reduced pressure. The remaining residue was acidified with 3 N HCl to pH = 1~2. The solid was collected by filtration and washed with hexane to give compound 5 (275 mg, 91%), R_f_ 0.4 (DCM/MeOH = 10:1, v/v) ^1^H NMR (600 MHz, CDCl_3_) δ 8.26 (d, *J* = 8.2 Hz, 2H), 8.11 (d, *J* = 7.7 Hz, 1H), 8.07 (s, 1H), 7.79 (d, *J* = 8.2 Hz, 2H), 7.43 (t, *J* = 7.7 Hz, 1H), 7.34 (d, *J* = 7.5 Hz, 1H), 2.47 (s, 3H).

*tert–Butyl(6–(5–(m–tolyl)–2–(4–(trifluoromethyl)phenyl)oxazole–4–carboxamido)hexyl)carbamate* (6)

To a solution of 5 (100 mg, 0.29mmol) were added *N*–(*tert*–butoxycarbonyl)–1,6–diaminohexane (78 μL, 0.35 mmol, 1.2 eq), HOBt (62 mg, 0.46 mmol, 1.6 eq), EDC–HCl (88 mg, 0.46 mmol, 1.6 eq) in DMF (10 mL). The reaction mixture was stirred for 6 h at room temperature. The solution was evaporated under reduced pressure. The residue was partitioned between ethyl acetate and water. The aqueous layer was further extracted with ethyl acetate. The combined organic layer was dried over MgSO_4_ and concentrated. The residue was purified by flash chromatography (0–20% EtOAc/Hexane) to give compound 6 (135 mg, 88%), R_f_ 0.04 (DCM/MeOH = 10:1, v/v) ^1^H NMR (600 MHz, CDCl_3_) δ 8.23 (d, *J* = 8.2 Hz, 2H), 8.20 – 8.16 (m, 2H), 7.77 (d, *J* = 8.2 Hz, 2H), 7.43 – 7.36 (m, 2H), 7.27 (s, 1H), 4.52 (s, 1H), 3.47 (dd, *J* = 13.5, 6.2 Hz, 2H), 3.12 (d, *J* = 6.2 Hz, 2H), 2.46 (s, 3H), 1.70 – 1.64 (m, 2H), 1.57 (s, 2H), 1.51 (dt, *J* = 14.3, 7.2 Hz, 2H), 1.44 (s, 9H), 1.41 – 1.36 (m, 2H).

*N–(6–Aminohexyl)–5–(m–tolyl)–2–(4–(trifluoromethyl)phenyl)oxazole–4–carboxamide* (7)

To a solution of 6 (122 mg, 0.23 mmol) in CH_2_Cl_2_ (9 mL) was added trifluoroacetic acid (3 mL). The reaction mixture was stirred at room temperature for 3 h. The reaction mixture was concentrated under reduced pressure to provide compound 7 (52 mg, 52%), R_f_ 0.03 DCM/MeOH = 10:1, v/v) ^1^H NMR (600 MHz, CDCl_3_) δ 8.19 (d, *J* = 8.0 Hz, 2H), 7.93 – 7.88 (m, 2H), 7.76 (d, *J* = 8.0 Hz, 2H), 7.65 (s, 1H), 7.37 (t, *J* = 7.6 Hz, 1H), 7.29 (d, *J* = 7.4 Hz, 1H), 3.47 (d, *J* = 5.6 Hz, 2H), 2.98 (s, 2H), 2.42 (s, 3H), 1.67 (s, 4H), 1.42 (d, *J* = 3.0 Hz, 4H).

*N–(6–(5–((3S,4S,6R)–2–Oxohexahydro–1H–thieno[3,4–d]imidazol–4–yl)pentanamido)hexyl)–5–(m–tolyl)–2–(4–(trifluoromethyl)phenyl)oxazole–4–carboxamide* (8)

To a solution of 7 (40 mg, 0.11mmol) were added biotin (33 mg, 0.13 mmol, 1.2 eq), HOBt (24 mg, 0.17 mmol, 1.6 eq), EDC–HCl (34 mg, 0.17 mmol, 1.6 eq) in DMF (10 mL). The reaction mixture was stirred for 6 h at room temperature. The solution was concentrated under reduced pressure. The residue was partitioned between ethyl acetate and water. The aqueous layer was further extracted with ethyl acetate. The combined organic layer was dried over MgSO_4_ and concentrated. The residue was purified by flash chromatography (0–20% EtOAc/Hexane) to give compound 8 (40 mg, 54%), R_f_ 0.4 (DCM/MeOH = 10:1, v/v) ^1^H NMR (600 MHz, MeOD) δ 8.38 – 8.32 (m, 2H), 8.11 (s, 1H), 8.06 (d, *J* = 8.0 Hz, 1H), 7.88 (d, *J* = 8.3 Hz, 2H), 7.40 (t, *J* = 7.7 Hz, 1H), 7.31 (d, *J* = 7.5 Hz, 1H), 4.58 (s, 1H), 4.45 (dd, *J* = 7.8, 4.8 Hz, 1H), 4.27 (dd, *J* = 7.8, 4.5 Hz, 1H), 3.43 (t, *J* = 7.1 Hz, 2H), 3.22 – 3.15 (m, 3H), 2.90 (dd, *J* = 12.7, 5.0 Hz, 1H), 2.68 (d, *J* = 12.7 Hz, 1H), 2.45 (s, 3H), 2.18 (t, *J* = 7.3 Hz, 2H), 1.72 – 1.58 (m, 8H), 1.47 – 1.40 (m, 6H). HRMS *m/z* : calcd for C_34_H_40_F_3_N_5_O_4_S [M+H]^+^ 672.2792; found, 672.2767

Supplementary Fig. 3. NMR data of KB2764 and biotinylated KB2764


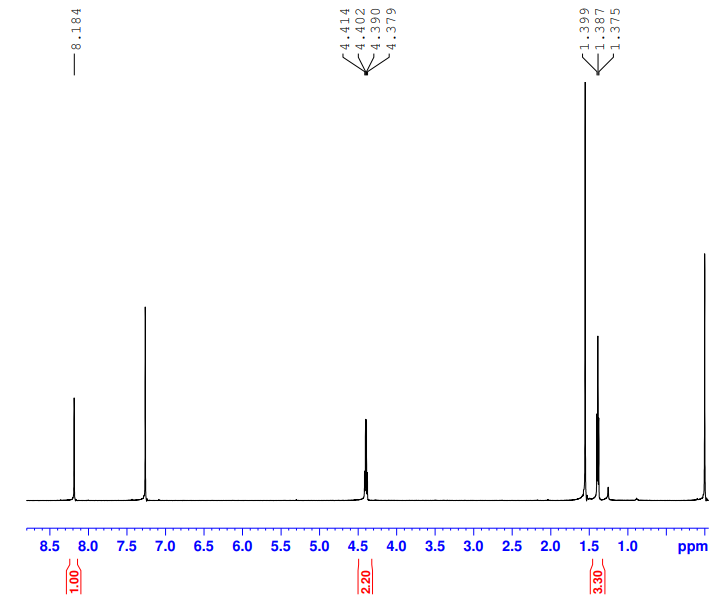


^1^H NMR spectra of compound **2** measured in CDCl_3_ at 600 MHz


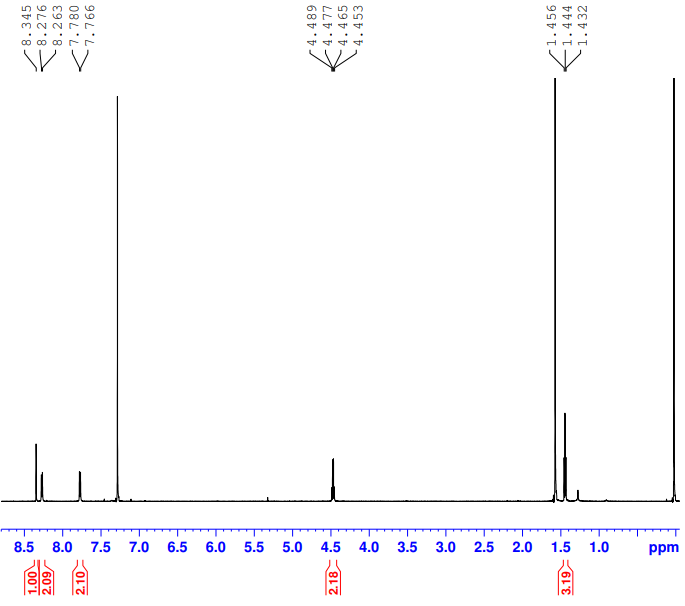


^1^H NMR spectra of compound 3 measured in CDCl_3_ at 600 MHz
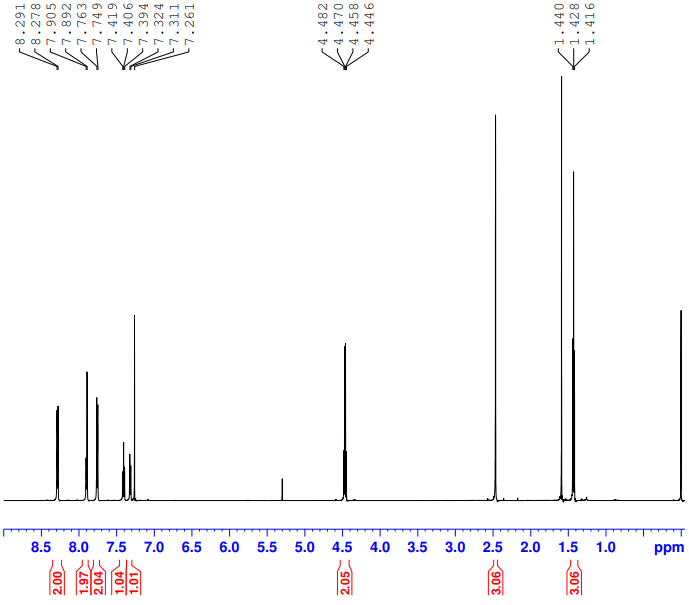


^1^H NMR spectra of compound 4 (KB2764) measured in CDCl_3_ at 600 MHz


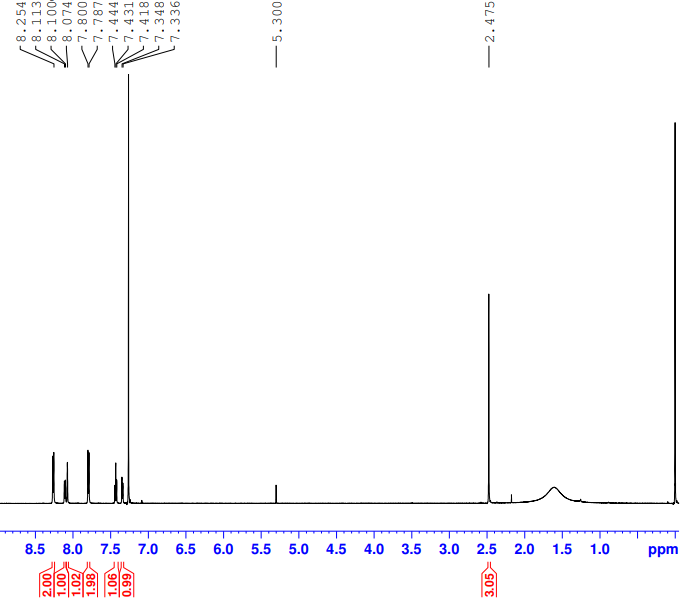


^1^H NMR spectra of compound 5 measured in CDCl_3_ at 600 MHz


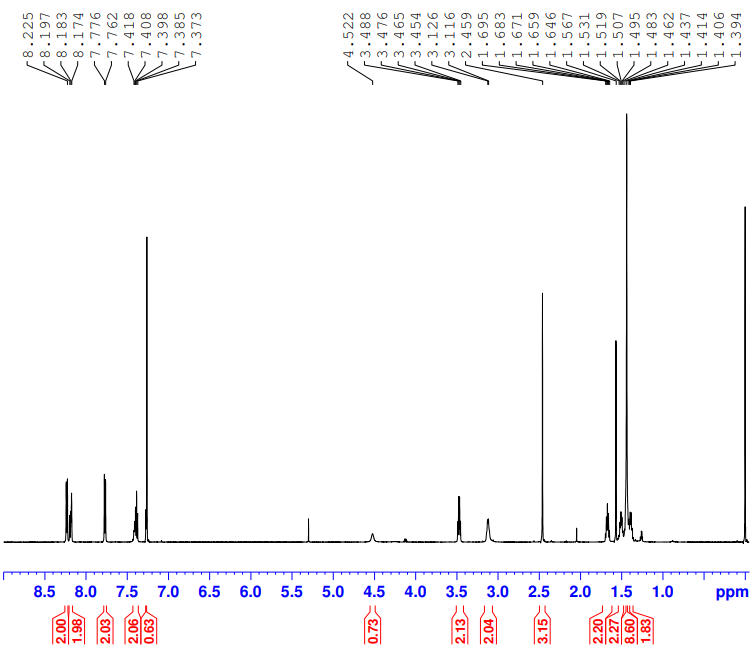


^1^H NMR spectra of compound 6 measured in CDCl_3_ at 600 MHz


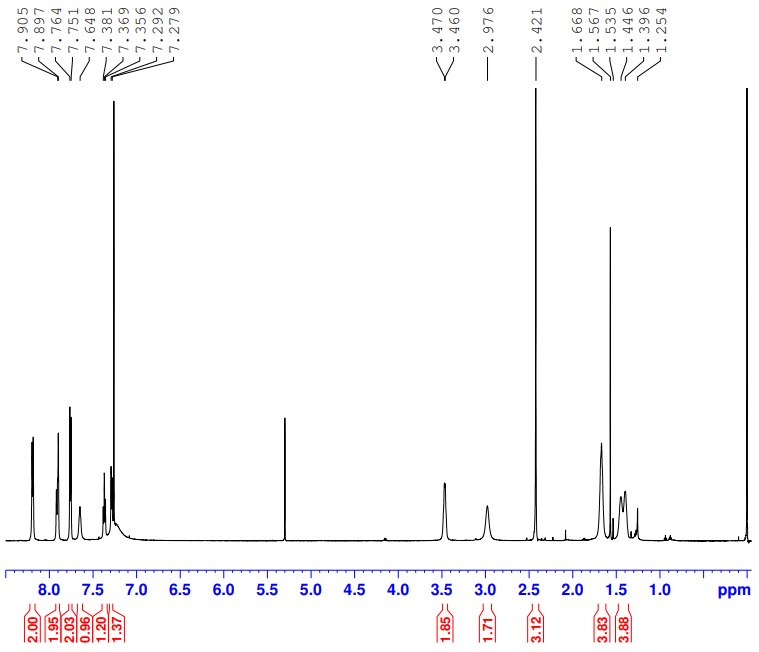


^1^H NMR spectra of compound **7** measured in CDCl_3_ at 600 MHz


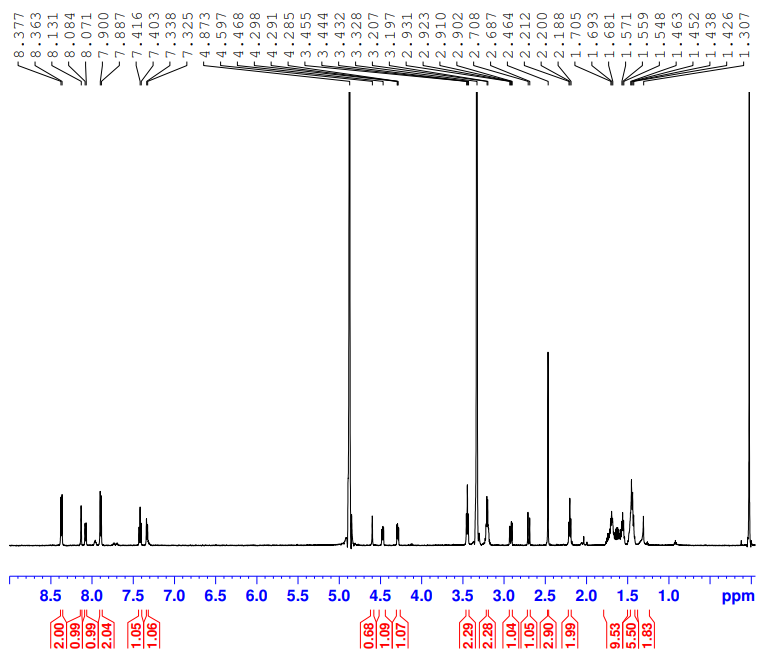


^1^H NMR spectra of compound 8 (Biotinylated KB2764) measured in MeOD at 600 MHz

Supplementary Fig. 4. HRMS data of KB2764 and biotinylated KB2764

HRMS data of compound 4 (KB2764)

HRMS date of compound 8 (Biotinylated KB2764)

Supplementary Fig. 5. HPLC data of KB2764 and biotinylated KB2764


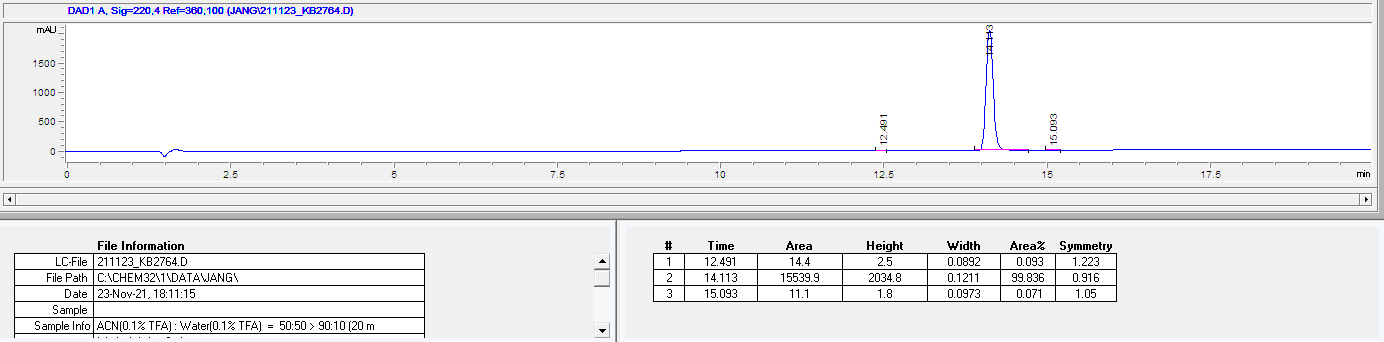


HPLC data of compound 4 (KB2764)


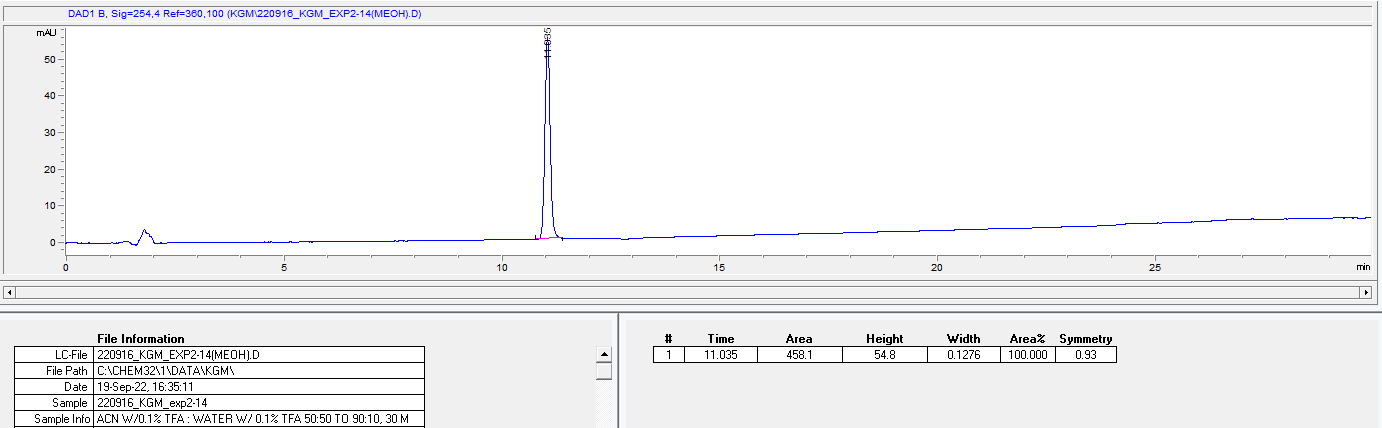


HPLC data of compound 8 (Biotinylated KB2764)


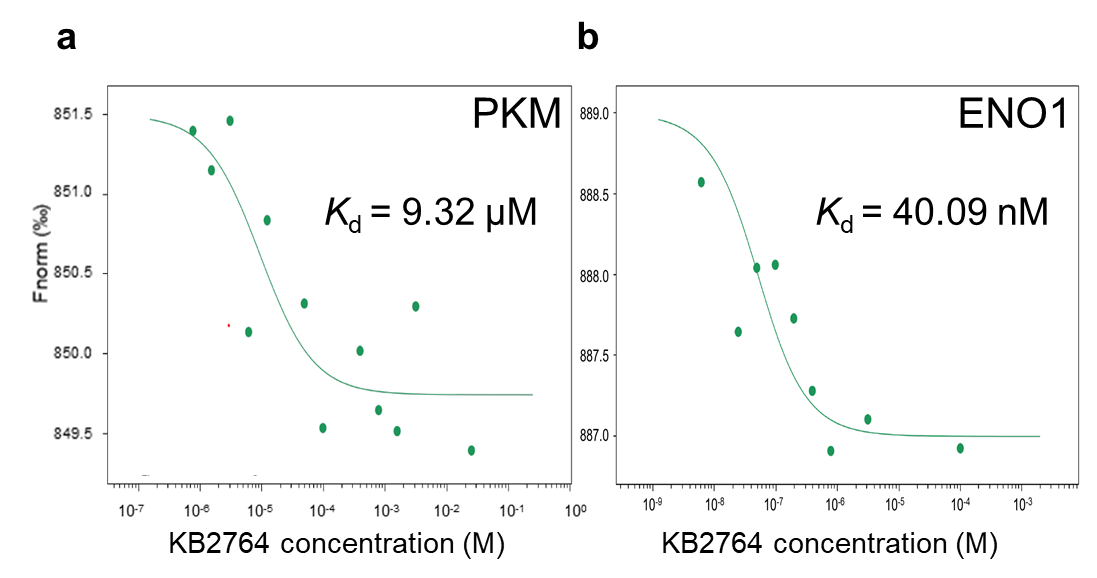


Supplementary Fig. 6. KB2764 binds more strongly to ENO1 protein than to PKM. (a and b) Microscale thermophoresis (MST) analysis was conducted to quantitatively evaluate the binding affinity. Data obtained was plotted with concentration on X-axis and Fnorm on Y-axis. Then, the dissociation constant (*K*_d_) was calculated. Fnorm value is calculated by dividing F_1_ by F_0_. F_1_ is the fluorescence value measured in the heated state, and F_0_ corresponds to the fluorescence value measured in the cold state before turning on the IR laser. The dissociation constant (*K*_d_) of PKM was 9.32 μM, indicating a weak interaction between PKM and KB2764. The *K*_d_ of ENO1 was 40.09 nM, indicating a strong interaction between ENO1 and KB2764.


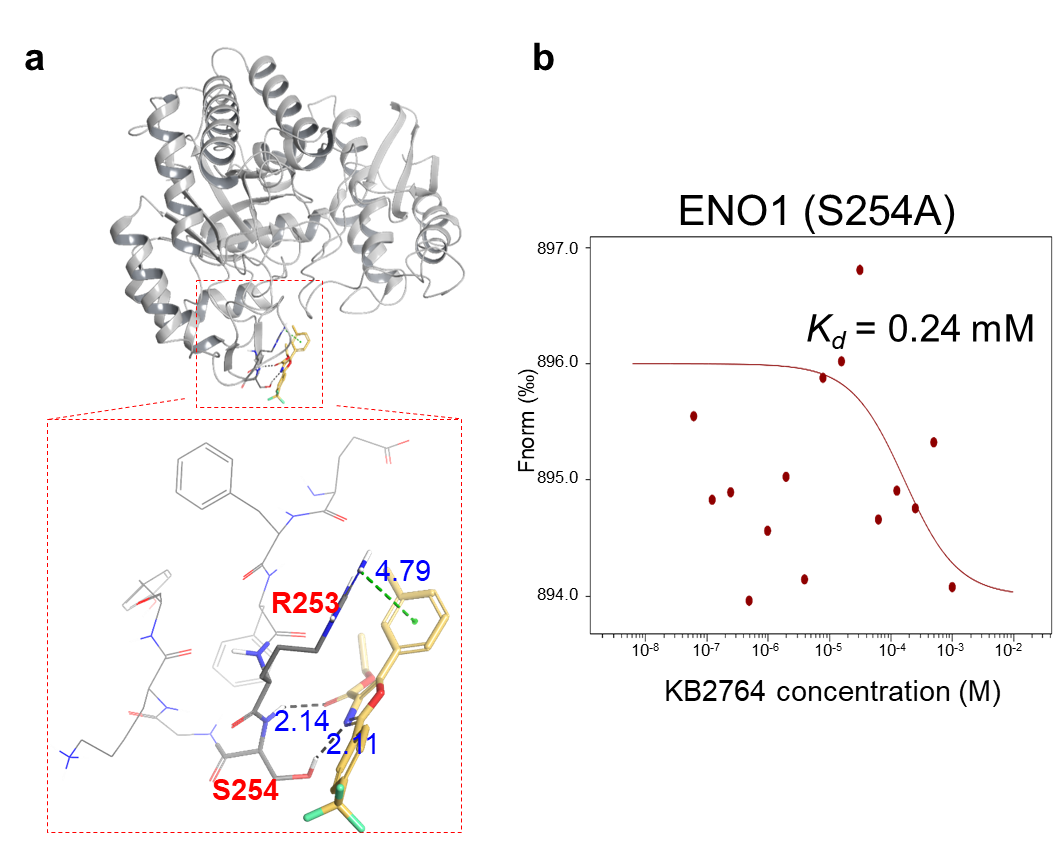


Supplementary Fig. 7. Ser 254 of ENO1 plays an important role in the binding between KB2764 and ENO1 protein. (a) *In silico* binding mode of the KB2764 in ENO1 (PDB ID: 3B97). Black and green dotted lines indicate hydrogen–bonding and π–cation interaction, respectively. Docking studies KB2764 with ENO1 showed that the best-docked pose was surrounded by Arg 253 and Ser 254. (b) Microscale thermophoresis (MST) analysis was conducted to quantitatively evaluate the binding affinity between KB2764 and ENO1 (S254A) protein. Data obtained was plotted with concentration on X-axis and Fnorm on Y-axis. Then, the dissociation constant (*K*_d_) was calculated. Fnorm value is calculated by dividing F_1_ by F_0_. F_1_ is the fluorescence value measured in the heated state, and F_0_ corresponds to the fluorescence value measured in the cold state before turning on the IR laser. *Kd* between KB2764 and ENO1 (S254A) protein was 0.24 mM.


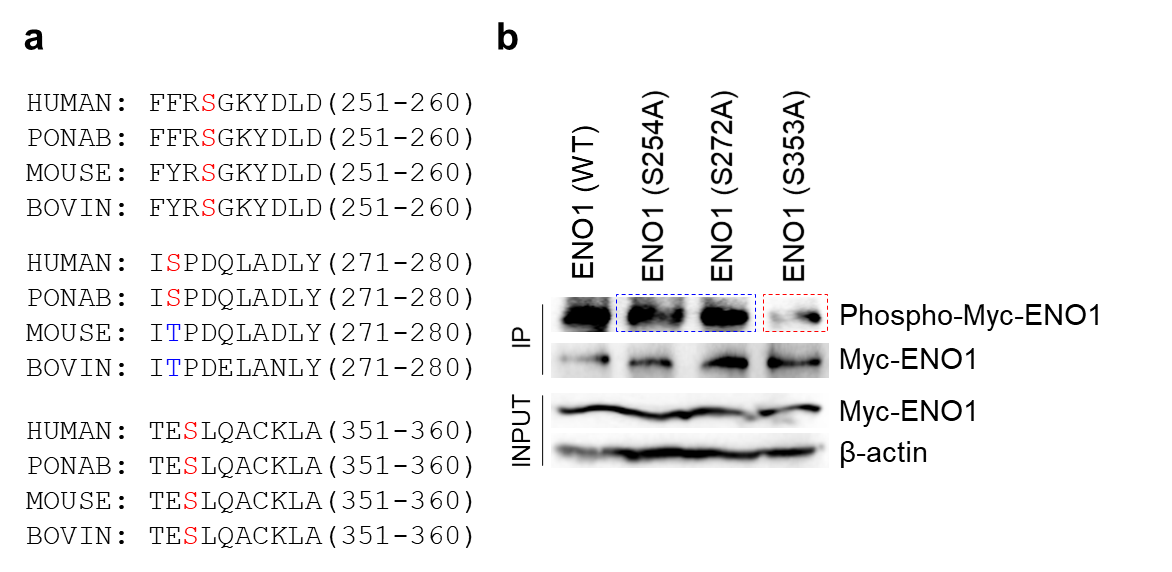


Supplementary Fig. 8. (a) The utilization of multiple sequence alignment has revealed the presence of a conserved motif in ENO1 across various species (human, ponab (*Pongo abelii*), mouse, and Bovin (Bovines)). The utilization of a kinase prediction tool allowed for the anticipation of a potential phosphorylation motif in ENO1 (<http://www.cbs.dtu.dk/services/NetPhos/>). Potential phosphorylation motifs were predicted to be serines (Ser) 254, 272 and 353, which are conserved across species. (b) Ser353 in ENO1 is identified as the potential phosphorylation motif. Cells were transfected with pCMV-Myc-ENO1 (WT), pCMV-Myc-ENO1 (S254A), pCMV-Myc-ENO1 (S272A), or pCMV-Myc-ENO1 (S353A). Immunoprecipitation was performed on cell lysates using an antibody against Myc-tag. ENO1 (WT), ENO1 (S254A), ENO1 (S272A), or ENO1 (S353A) phosphorylation was detected by phospho-Ser antibody. Phosphorylation of ENO1 (S254A) and ENO1 (S272A) was not reduced compared to ENO1 (WT) (blue boxed areas). Phosphorylation of ENO1 (S353A) markedly was compared to ENO1 (WT) (red boxed areas).

Supplementary Fig. 9. The effect of biotinylation on the function of KB2764. Flow cytometric analysis of reactive oxygen species (ROS) using DHR123. ***P* < 0.01, Student t–test. Mean ± S.D., *n* = 3.

Supplementary table 1. List of 149 proteins that bind only to biotinylated KB2764 and not to biotin.

| Number | Accession | -10lgP | Avg. Mass | Description |
| --- | --- | --- | --- | --- |
| 1 | Q16352\|AINX_HUMAN | 195.75 | 55391 | Alpha-internexin OS=Homo sapiens OX=9606 GN=INA PE=1 SV=2 |
| 2 | P07196\|NFL_HUMAN | 260.77 | 61517 | Neurofilament light polypeptide OS=Homo sapiens OX=9606 GN=NEFL PE=1 SV=3 |
| 3 | Q07065\|CKAP4_HUMAN | 235.03 | 66023 | Cytoskeleton-associated protein 4 OS=Homo sapiens OX=9606 GN=CKAP4 PE=1 SV=2 |
| 4 | P10412\|H14_HUMAN | 232.94 | 21865 | Histone H1.4 OS=Homo sapiens OX=9606 GN=H1-4 PE=1 SV=2 |
| 5 | tr\|H0YA55\|H0YA55_HUMAN | 161.52 | 51578 | Albumin (Fragment) OS=Homo sapiens OX=9606 GN=ALB PE=1 SV=1 |
| 6 | Q562R1\|ACTBL_HUMAN | 194.94 | 42003 | Beta-actin-like protein 2 OS=Homo sapiens OX=9606 GN=ACTBL2 PE=1 SV=2 |
| 7 | P16403\|H12_HUMAN | 216.37 | 21365 | Histone H1.2 OS=Homo sapiens OX=9606 GN=H1-2 PE=1 SV=2 |
| 8 | P09382\|LEG1_HUMAN | 141.32 | 14716 | Galectin-1 OS=Homo sapiens OX=9606 GN=LGALS1 PE=1 SV=2 |
| 9 | P48681\|NEST_HUMAN | 187.02 | 177438 | Nestin OS=Homo sapiens OX=9606 GN=NES PE=1 SV=2 |
| 10 | P14854\|CX6B1_HUMAN | 154.27 | 10192 | Cytochrome c oxidase subunit 6B1 OS=Homo sapiens OX=9606 GN=COX6B1 PE=1 SV=2 |
| 11 | tr\|Q60FE5\|Q60FE5_HUMAN | 202.92 | 278224 | Filamin-A OS=Homo sapiens OX=9606 GN=FLNA PE=1 SV=1 |
| 12 | tr\|H3BNX8\|H3BNX8_HUMAN | 136.07 | 17235 | Cytochrome c oxidase polypeptide Va OS=Homo sapiens OX=9606 GN=COX5A PE=1 SV=1 |
| 13 | P20674\|COX5A_HUMAN | 136.07 | 16762 | Cytochrome c oxidase subunit 5A mitochondrial OS=Homo sapiens OX=9606 GN=COX5A PE=1 SV=2 |
| 14 | tr\|H0YMU9\|H0YMU9_HUMAN | 159.93 | 25569 | Annexin OS=Homo sapiens OX=9606 GN=ANXA2 PE=1 SV=1 |
| 15 | P16401\|H15_HUMAN | 208.96 | 22580 | Histone H1.5 OS=Homo sapiens OX=9606 GN=H1-5 PE=1 SV=3 |
| 16 | P10606\|COX5B_HUMAN | 122.17 | 13696 | Cytochrome c oxidase subunit 5B mitochondrial OS=Homo sapiens OX=9606 GN=COX5B PE=1 SV=2 |
| 17 | P62807\|H2B1C_HUMAN | 145.94 | 13906 | Histone H2B type 1-C/E/F/G/I OS=Homo sapiens OX=9606 GN=H2BC4 PE=1 SV=4 |
| 18 | Q16778\|H2B2E_HUMAN | 145.94 | 13920 | Histone H2B type 2-E OS=Homo sapiens OX=9606 GN=H2BC21 PE=1 SV=3 |
| 19 | P06899\|H2B1J_HUMAN | 141.57 | 13904 | Histone H2B type 1-J OS=Homo sapiens OX=9606 GN=H2BC11 PE=1 SV=3 |
| 20 | O60814\|H2B1K_HUMAN | 141.57 | 13890 | Histone H2B type 1-K OS=Homo sapiens OX=9606 GN=H2BC12 PE=1 SV=3 |
| 21 | P57053\|H2BFS_HUMAN | 141.57 | 13944 | Histone H2B type F-S OS=Homo sapiens OX=9606 GN=H2BS1 PE=1 SV=2 |
| 22 | P0DP23\|CALM1_HUMAN | 97.36 | 16838 | Calmodulin-1 OS=Homo sapiens OX=9606 GN=CALM1 PE=1 SV=1 |
| 23 | P0DP24\|CALM2_HUMAN | 97.36 | 16838 | Calmodulin-2 OS=Homo sapiens OX=9606 GN=CALM2 PE=1 SV=1 |
| 24 | P0DP25\|CALM3_HUMAN | 97.36 | 16838 | Calmodulin-3 OS=Homo sapiens OX=9606 GN=CALM3 PE=1 SV=1 |
| 25 | tr\|E5RI98\|E5RI98_HUMAN | 139.57 | 25294 | Nucleophosmin OS=Homo sapiens OX=9606 GN=NPM1 PE=1 SV=2 |
| 26 | Q15149\|PLEC_HUMAN | 157.67 | 531796 | Plectin OS=Homo sapiens OX=9606 GN=PLEC PE=1 SV=3 |
| 27 | P52926\|HMGA2_HUMAN | 106.05 | 11832 | High mobility group protein HMGI-C OS=Homo sapiens OX=9606 GN=HMGA2 PE=1 SV=1 |
| 28 | Q9UHB6\|LIMA1_HUMAN | 183.61 | 85226 | LIM domain and actin-binding protein 1 OS=Homo sapiens OX=9606 GN=LIMA1 PE=1 SV=1 |
| 29 | tr\|A0A7P0TB45\|A0A7P0TB45_HUMAN | 111.44 | 23813 | Peptidyl-prolyl cis-trans isomerase OS=Homo sapiens OX=9606 GN=PPIB PE=1 SV=1 |
| 30 | P62861\|RS30_HUMAN | 110.56 | 6648 | 40S ribosomal protein S30 OS=Homo sapiens OX=9606 GN=FAU PE=1 SV=1 |
| 31 | P38606\|VATA_HUMAN | 108.5 | 68304 | V-type proton ATPase catalytic subunit A OS=Homo sapiens OX=9606 GN=ATP6V1A PE=1 SV=2 |
| 32 | P28799\|GRN_HUMAN | 100.2 | 63545 | Progranulin OS=Homo sapiens OX=9606 GN=GRN PE=1 SV=2 |
| 33 | P14618\|KPYM_HUMAN | 130.78 | 57937 | Pyruvate kinase PKM OS=Homo sapiens OX=9606 GN=PKM PE=1 SV=4 |
| 34 | P27105\|STOM_HUMAN | 97.79 | 31731 | Stomatin OS=Homo sapiens OX=9606 GN=STOM PE=1 SV=3 |
| 35 | Q05639\|EF1A2_HUMAN | 107.97 | 50470 | Elongation factor 1-alpha 2 OS=Homo sapiens OX=9606 GN=EEF1A2 PE=1 SV=1 |
| 36 | tr\|A0A1W2PPS1\|A0A1W2PPS1_HUMAN | 94.16 | 88318 | Heterogeneous nuclear ribonucleoprotein U OS=Homo sapiens OX=9606 GN=HNRNPU PE=1 SV=1 |
| 37 | tr\|A0A024RA28\|A0A024RA28_HUMAN | 103.87 | 31163 | Heterogeneous nuclear ribonucleoprotein A2/B1 isoform CRA_d OS=Homo sapiens OX=9606 GN=HNRNPA2B1 PE=1 SV=1 |
| 38 | tr\|A0A7I2V323\|A0A7I2V323_HUMAN | 103.87 | 35222 | Heterogeneous nuclear ribonucleoproteins A2/B1 OS=Homo sapiens OX=9606 GN=HNRNPA2B1 PE=1 SV=1 |
| 39 | Q9H299\|SH3L3_HUMAN | 69.24 | 10438 | SH3 domain-binding glutamic acid-rich-like protein 3 OS=Homo sapiens OX=9606 GN=SH3BGRL3 PE=1 SV=1 |
| 40 | tr\|H0Y6E7\|H0Y6E7_HUMAN | 68.63 | 31865 | RNA-binding motif protein X chromosome (Fragment) OS=Homo sapiens OX=9606 GN=RBMX PE=1 SV=2 |
| 41 | tr\|H3BT71\|H3BT71_HUMAN | 68.63 | 32197 | RNA-binding motif protein X chromosome OS=Homo sapiens OX=9606 GN=RBMX PE=1 SV=1 |
| 42 | P38159\|RBMX_HUMAN | 68.63 | 42332 | RNA-binding motif protein X chromosome OS=Homo sapiens OX=9606 GN=RBMX PE=1 SV=3 |
| 43 | tr\|A0A7I2V2X6\|A0A7I2V2X6_HUMAN | 112.46 | 57759 | 60 kDa chaperonin OS=Homo sapiens OX=9606 GN=HSPD1 PE=1 SV=1 |
| 44 | P62280\|RS11_HUMAN | 93.29 | 18431 | 40S ribosomal protein S11 OS=Homo sapiens OX=9606 GN=RPS11 PE=1 SV=3 |
| 45 | P07305\|H10_HUMAN | 91.2 | 20863 | Histone H1.0 OS=Homo sapiens OX=9606 GN=H1-0 PE=1 SV=3 |
| 46 | Q92743\|HTRA1_HUMAN | 93.44 | 51287 | Serine protease HTRA1 OS=Homo sapiens OX=9606 GN=HTRA1 PE=1 SV=1 |
| 47 | tr\|A0A6Q8PGK1\|A0A6Q8PGK1_HUMAN | 72.4 | 23705 | Heat shock 27 kDa protein OS=Homo sapiens OX=9606 GN=HSPB1 PE=1 SV=1 |
| 48 | tr\|A0A6Q8PFK8\|A0A6Q8PFK8_HUMAN | 72.4 | 21882 | Heat shock protein beta-1 OS=Homo sapiens OX=9606 GN=HSPB1 PE=1 SV=1 |
| 49 | P63104\|1433Z_HUMAN | 80.65 | 27745 | 14-3-3 protein zeta/delta OS=Homo sapiens OX=9606 GN=YWHAZ PE=1 SV=1 |
| 50 | tr\|A0A7P0TAI0\|A0A7P0TAI0_HUMAN | 108.5 | 68092 | 78 kDa glucose-regulated protein OS=Homo sapiens OX=9606 GN=HSPA5 PE=1 SV=1 |
| 51 | P11021\|BIP_HUMAN | 108.5 | 72333 | Endoplasmic reticulum chaperone BiP OS=Homo sapiens OX=9606 GN=HSPA5 PE=1 SV=2 |
| 52 | tr\|B2R5W2\|B2R5W2_HUMAN | 94.57 | 31948 | Heterogeneous nuclear ribonucleoproteins C1/C2 OS=Homo sapiens OX=9606 GN=HNRNPC PE=1 SV=1 |
| 53 | tr\|A0A7I2V3W0\|A0A7I2V3W0_HUMAN | 104.47 | 34964 | Helix-destabilizing protein OS=Homo sapiens OX=9606 GN=HNRNPA1 PE=1 SV=1 |
| 54 | P09651\|ROA1_HUMAN | 104.47 | 38747 | Heterogeneous nuclear ribonucleoprotein A1 OS=Homo sapiens OX=9606 GN=HNRNPA1 PE=1 SV=5 |
| 55 | tr\|A0A7I2V4Y4\|A0A7I2V4Y4_HUMAN | 83.5 | 103606 | Alpha-actinin-1 OS=Homo sapiens OX=9606 GN=ACTN1 PE=1 SV=1 |
| 56 | tr\|B3KWK7\|B3KWK7_HUMAN | 72.18 | 21844 | Insulin-like growth factor binding protein 3 isoform CRA_b OS=Homo sapiens OX=9606 GN=IGFBP3 PE=1 SV=1 |
| 57 | tr\|A6XND0\|A6XND0_HUMAN | 72.18 | 29747 | Insulin-like growth factor-binding protein 3 OS=Homo sapiens OX=9606 GN=IGFBP3 PE=1 SV=1 |
| 58 | tr\|Q86WV2\|Q86WV2_HUMAN | 79.65 | 9291 | COX4I1 protein OS=Homo sapiens OX=9606 GN=COX4I1 PE=1 SV=1 |
| 59 | P13073\|COX41_HUMAN | 79.65 | 19577 | Cytochrome c oxidase subunit 4 isoform 1 mitochondrial OS=Homo sapiens OX=9606 GN=COX4I1 PE=1 SV=1 |
| 60 | tr\|H3BN72\|H3BN72_HUMAN | 79.65 | 14369 | Cytochrome c oxidase subunit 4 OS=Homo sapiens OX=9606 GN=COX4I1 PE=1 SV=1 |
| 61 | tr\|A0A7I2V4K9\|A0A7I2V4K9_HUMAN | 68.54 | 47746 | Non-POU domain-containing octamer-binding protein OS=Homo sapiens OX=9606 GN=NONO PE=1 SV=1 |
| 62 | P62318\|SMD3_HUMAN | 101.25 | 13916 | Small nuclear ribonucleoprotein Sm D3 OS=Homo sapiens OX=9606 GN=SNRPD3 PE=1 SV=1 |
| 63 | tr\|F8VZQ9\|F8VZQ9_HUMAN | 87.59 | 24105 | SAP domain-containing ribonucleoprotein OS=Homo sapiens OX=9606 GN=SARNP PE=1 SV=1 |
| 64 | P82979\|SARNP_HUMAN | 87.59 | 23671 | SAP domain-containing ribonucleoprotein OS=Homo sapiens OX=9606 GN=SARNP PE=1 SV=3 |
| 65 | tr\|A0A590UJJ6\|A0A590UJJ6_HUMAN | 67.59 | 13655 | Histone H3 OS=Homo sapiens OX=9606 GN=H3-3A PE=1 SV=1 |
| 66 | tr\|K7EMV3\|K7EMV3_HUMAN | 67.59 | 10334 | Histone H3 OS=Homo sapiens OX=9606 GN=H3-3B PE=1 SV=1 |
| 67 | P68431\|H31_HUMAN | 67.59 | 15404 | Histone H3.1 OS=Homo sapiens OX=9606 GN=H3C1 PE=1 SV=2 |
| 68 | Q16695\|H31T_HUMAN | 67.59 | 15508 | Histone H3.1t OS=Homo sapiens OX=9606 GN=H3-4 PE=1 SV=3 |
| 69 | Q71DI3\|H32_HUMAN | 67.59 | 15388 | Histone H3.2 OS=Homo sapiens OX=9606 GN=H3C15 PE=1 SV=3 |
| 70 | P84243\|H33_HUMAN | 67.59 | 15328 | Histone H3.3 OS=Homo sapiens OX=9606 GN=H3-3A PE=1 SV=2 |
| 71 | tr\|K7EP01\|K7EP01_HUMAN | 67.59 | 12808 | Histone H3.3 OS=Homo sapiens OX=9606 GN=H3-3B PE=1 SV=1 |
| 72 | Q6NXT2\|H3C_HUMAN | 67.59 | 15214 | Histone H3.3C OS=Homo sapiens OX=9606 GN=H3-5 PE=1 SV=3 |
| 73 | Q5TEC6\|H3PS2_HUMAN | 67.59 | 15430 | Histone HIST2H3PS2 OS=Homo sapiens OX=9606 GN=H3-2 PE=1 SV=1 |
| 74 | Q71UI9\|H2AV_HUMAN | 73.52 | 13509 | Histone H2A.V OS=Homo sapiens OX=9606 GN=H2AZ2 PE=1 SV=3 |
| 75 | P0C0S5\|H2AZ_HUMAN | 73.52 | 13553 | Histone H2A.Z OS=Homo sapiens OX=9606 GN=H2AZ1 PE=1 SV=2 |
| 76 | tr\|E9PP50\|E9PP50_HUMAN | 82.71 | 17778 | Cofilin non-muscle isoform (Fragment) OS=Homo sapiens OX=9606 GN=CFL1 PE=1 SV=8 |
| 77 | P62805\|H4_HUMAN | 66.25 | 11367 | Histone H4 OS=Homo sapiens OX=9606 GN=H4C1 PE=1 SV=2 |
| 78 | tr\|A0A0A0MSK5\|A0A0A0MSK5_HUMAN | 69.83 | 52406 | Torsin-1A-interacting protein 1 OS=Homo sapiens OX=9606 GN=TOR1AIP1 PE=1 SV=1 |
| 79 | P47985\|UCRI_HUMAN | 65.16 | 29668 | Cytochrome b-c1 complex subunit Rieske mitochondrial OS=Homo sapiens OX=9606 GN=UQCRFS1 PE=1 SV=2 |
| 80 | P0C7P4\|UCRIL_HUMAN | 65.16 | 30815 | Putative cytochrome b-c1 complex subunit Rieske-like protein 1 OS=Homo sapiens OX=9606 GN=UQCRFS1P1 PE=5 SV=1 |
| 81 | P62937\|PPIA_HUMAN | 68.12 | 18012 | Peptidyl-prolyl cis-trans isomerase A OS=Homo sapiens OX=9606 GN=PPIA PE=1 SV=2 |
| 82 | P62328\|TYB4_HUMAN | 51.78 | 5053 | Thymosin beta-4 OS=Homo sapiens OX=9606 GN=TMSB4X PE=1 SV=2 |
| 83 | Q99536\|VAT1_HUMAN | 64.58 | 41920 | Synaptic vesicle membrane protein VAT-1 homolog OS=Homo sapiens OX=9606 GN=VAT1 PE=1 SV=2 |
| 84 | tr\|D6RER5\|D6RER5_HUMAN | 83.84 | 49809 | Septin OS=Homo sapiens OX=9606 GN=SEPTIN11 PE=1 SV=1 |
| 85 | P31930\|QCR1_HUMAN | 71.41 | 52646 | Cytochrome b-c1 complex subunit 1 mitochondrial OS=Homo sapiens OX=9606 GN=UQCRC1 PE=1 SV=3 |
| 86 | tr\|D6RBT3\|D6RBT3_HUMAN | 66.37 | 18752 | NADH dehydrogenase [ubiquinone] iron-sulfur protein 6 mitochondrial OS=Homo sapiens OX=9606 GN=NDUFS6 PE=1 SV=1 |
| 87 | tr\|E7ENY0\|E7ENY0_HUMAN | 61.24 | 73404 | Alpha-adducin OS=Homo sapiens OX=9606 GN=ADD1 PE=1 SV=1 |
| 88 | Q14974\|IMB1_HUMAN | 57.8 | 97170 | Importin subunit beta-1 OS=Homo sapiens OX=9606 GN=KPNB1 PE=1 SV=2 |
| 89 | Q15293\|RCN1_HUMAN | 39.83 | 38890 | Reticulocalbin-1 OS=Homo sapiens OX=9606 GN=RCN1 PE=1 SV=1 |
| 90 | tr\|E9PB61\|E9PB61_HUMAN | 70.83 | 27558 | THO complex subunit 4 OS=Homo sapiens OX=9606 GN=ALYREF PE=1 SV=1 |
| 91 | tr\|A0A6Q8PFY6\|A0A6Q8PFY6_HUMAN | 49.02 | 79916 | Atriopeptidase OS=Homo sapiens OX=9606 GN=MME PE=1 SV=1 |
| 92 | P08473\|NEP_HUMAN | 49.02 | 85514 | Neprilysin OS=Homo sapiens OX=9606 GN=MME PE=1 SV=2 |
| 93 | Q5SSJ5\|HP1B3_HUMAN | 80.49 | 61207 | Heterochromatin protein 1-binding protein 3 OS=Homo sapiens OX=9606 GN=HP1BP3 PE=1 SV=1 |
| 94 | P23246\|SFPQ_HUMAN | 78.02 | 76150 | Splicing factor proline- and glutamine-rich OS=Homo sapiens OX=9606 GN=SFPQ PE=1 SV=2 |
| 95 | Q13200\|PSMD2_HUMAN | 78.41 | 100200 | 26S proteasome non-ATPase regulatory subunit 2 OS=Homo sapiens OX=9606 GN=PSMD2 PE=1 SV=3 |
| 96 | Q9Y3I0\|RTCB_HUMAN | 59.54 | 55210 | RNA-splicing ligase RtcB homolog OS=Homo sapiens OX=9606 GN=RTCB PE=1 SV=1 |
| 97 | tr\|A0A087X1B7\|A0A087X1B7_HUMAN | 55.72 | 8520 | Chromatin target of PRMT1 protein OS=Homo sapiens OX=9606 GN=CHTOP PE=1 SV=1 |
| 98 | Q9NX14\|NDUBB_HUMAN | 54.69 | 17317 | NADH dehydrogenase [ubiquinone] 1 beta subcomplex subunit 11 mitochondrial OS=Homo sapiens OX=9606 GN=NDUFB11 PE=1 SV=1 |
| 99 | P15144\|AMPN_HUMAN | 70.93 | 109539 | Aminopeptidase N OS=Homo sapiens OX=9606 GN=ANPEP PE=1 SV=4 |
| 100 | O00148\|DX39A_HUMAN | 52.64 | 49130 | ATP-dependent RNA helicase DDX39A OS=Homo sapiens OX=9606 GN=DDX39A PE=1 SV=2 |
| 101 | tr\|Q5STU3\|Q5STU3_HUMAN | 52.64 | 48826 | RNA helicase OS=Homo sapiens OX=9606 GN=DDX39B PE=1 SV=2 |
| 102 | Q13838\|DX39B_HUMAN | 52.64 | 48991 | Spliceosome RNA helicase DDX39B OS=Homo sapiens OX=9606 GN=DDX39B PE=1 SV=1 |
| 103 | tr\|G3V1N2\|G3V1N2_HUMAN | 58.8 | 11948 | HCG1745306 isoform CRA_a OS=Homo sapiens OX=9606 GN=HBA2 PE=1 SV=1 |
| 104 | P69905\|HBA_HUMAN | 58.8 | 15258 | Hemoglobin subunit alpha OS=Homo sapiens OX=9606 GN=HBA1 PE=1 SV=2 |
| 105 | tr\|J3QL14\|J3QL14_HUMAN | 45.11 | 26824 | V-type proton ATPase subunit d 1 (Fragment) OS=Homo sapiens OX=9606 GN=ATP6V0D1 PE=1 SV=1 |
| 106 | tr\|F5GYQ1\|F5GYQ1_HUMAN | 45.11 | 44660 | V-type proton ATPase subunit OS=Homo sapiens OX=9606 GN=ATP6V0D1 PE=1 SV=1 |
| 107 | P01023\|A2MG_HUMAN | 46.71 | 163290 | Alpha-2-macroglobulin OS=Homo sapiens OX=9606 GN=A2M PE=1 SV=3 |
| 108 | tr\|E9PIE3\|E9PIE3_HUMAN | 43.04 | 31088 | Caveolae-associated protein 3 OS=Homo sapiens OX=9606 GN=CAVIN3 PE=1 SV=1 |
| 109 | O75955\|FLOT1_HUMAN | 40.08 | 47355 | Flotillin-1 OS=Homo sapiens OX=9606 GN=FLOT1 PE=1 SV=3 |
| 110 | P21589\|5NTD_HUMAN | 40.74 | 63368 | 5'-nucleotidase OS=Homo sapiens OX=9606 GN=NT5E PE=1 SV=1 |
| 111 | tr\|Q96B60\|Q96B60_HUMAN | 40.74 | 28841 | Ecto-5'-nucleotidase OS=Homo sapiens OX=9606 GN=NT5E PE=1 SV=1 |
| 112 | tr\|A0A087WYW3\|A0A087WYW3_HUMAN | 33.57 | 131823 | NK-tumor recognition protein OS=Homo sapiens OX=9606 GN=NKTR PE=1 SV=1 |
| 113 | Q9Y4C1\|KDM3A_HUMAN | 28.81 | 147341 | Lysine-specific demethylase 3A OS=Homo sapiens OX=9606 GN=KDM3A PE=1 SV=4 |
| 114 | tr\|A0A087X0M7\|A0A087X0M7_HUMAN | 30.12 | 68018 | Protein FAM13C OS=Homo sapiens OX=9606 GN=FAM13C PE=1 SV=1 |
| 115 | tr\|A0A7I2U3A3\|A0A7I2U3A3_HUMAN | 33.69 | 29641 | Phosphatidylinositol 3-kinase regulatory subunit beta OS=Homo sapiens OX=9606 GN=PIK3R2 PE=1 SV=1 |
| 116 | Q9NX63\|MIC19_HUMAN | 37.92 | 26152 | MICOS complex subunit MIC19 OS=Homo sapiens OX=9606 GN=CHCHD3 PE=1 SV=1 |
| 117 | Q9Y6C9\|MTCH2_HUMAN | 68.58 | 33331 | Mitochondrial carrier homolog 2 OS=Homo sapiens OX=9606 GN=MTCH2 PE=1 SV=1 |
| 118 | tr\|D6RCD7\|D6RCD7_HUMAN | 53.62 | 72251 | 75 kDa glucose-regulated protein OS=Homo sapiens OX=9606 GN=HSPA9 PE=1 SV=2 |
| 119 | tr\|A0A7I2V3F7\|A0A7I2V3F7_HUMAN | 53.62 | 65856 | Stress-70 protein mitochondrial OS=Homo sapiens OX=9606 GN=HSPA9 PE=1 SV=1 |
| 120 | tr\|V9GYG0\|V9GYG0_HUMAN | 36.55 | 22885 | ADP/ATP translocase OS=Homo sapiens OX=9606 GN=SLC25A4 PE=1 SV=1 |
| 121 | P13987\|CD59_HUMAN | 29.39 | 14177 | CD59 glycoprotein OS=Homo sapiens OX=9606 GN=CD59 PE=1 SV=1 |
| 122 | Q02978\|M2OM_HUMAN | 65.6 | 34062 | Mitochondrial 2-oxoglutarate/malate carrier protein OS=Homo sapiens OX=9606 GN=SLC25A11 PE=1 SV=3 |
| 123 | P38919\|IF4A3_HUMAN | 62.97 | 46871 | Eukaryotic initiation factor 4A-III OS=Homo sapiens OX=9606 GN=EIF4A3 PE=1 SV=4 |
| 124 | tr\|I3L3H2\|I3L3H2_HUMAN | 62.97 | 44511 | RNA helicase OS=Homo sapiens OX=9606 GN=EIF4A3 PE=1 SV=2 |
| 125 | tr\|E5RHH0\|E5RHH0_HUMAN | 58.81 | 11663 | V-type proton ATPase subunit H (Fragment) OS=Homo sapiens OX=9606 GN=ATP6V1H PE=1 SV=1 |
| 126 | P36543\|VATE1_HUMAN | 56.95 | 26145 | V-type proton ATPase subunit E 1 OS=Homo sapiens OX=9606 GN=ATP6V1E1 PE=1 SV=1 |
| 127 | P52272\|HNRPM_HUMAN | 54.45 | 77516 | Heterogeneous nuclear ribonucleoprotein M OS=Homo sapiens OX=9606 GN=HNRNPM PE=1 SV=3 |
| 128 | Q07666\|KHDR1_HUMAN | 47.37 | 48227 | KH domain-containing RNA-binding signal transduction-associated protein 1 OS=Homo sapiens OX=9606 GN=KHDRBS1 PE=1 SV=1 |
| 129 | Q96C19\|EFHD2_HUMAN | 48.04 | 26697 | EF-hand domain-containing protein D2 OS=Homo sapiens OX=9606 GN=EFHD2 PE=1 SV=1 |
| 130 | tr\|A0A2R8YEM5\|A0A2R8YEM5_HUMAN | 44.72 | 18381 | 2-phospho-D-glycerate hydro-lyase (Fragment) OS=Homo sapiens OX=9606 GN=ENO1 PE=1 SV=1 |
| 131 | P06733\|ENOA_HUMAN | 44.72 | 47169 | Alpha-enolase OS=Homo sapiens OX=9606 GN=ENO1 PE=1 SV=2 |
| 132 | tr\|H0YKH2\|H0YKH2_HUMAN | 45.41 | 10815 | SAFB-like transcription modulator (Fragment) OS=Homo sapiens OX=9606 GN=SLTM PE=1 SV=1 |
| 133 | Q9NWH9\|SLTM_HUMAN | 45.41 | 117149 | SAFB-like transcription modulator OS=Homo sapiens OX=9606 GN=SLTM PE=1 SV=2 |
| 134 | Q9NYU2\|UGGG1_HUMAN | 46.01 | 177189 | UDP-glucose:glycoprotein glucosyltransferase 1 OS=Homo sapiens OX=9606 GN=UGGT1 PE=1 SV=3 |
| 135 | Q14568\|HS902_HUMAN | 40.17 | 39365 | Heat shock protein HSP 90-alpha A2 OS=Homo sapiens OX=9606 GN=HSP90AA2P PE=1 SV=2 |
| 136 | P07900\|HS90A_HUMAN | 40.17 | 84660 | Heat shock protein HSP 90-alpha OS=Homo sapiens OX=9606 GN=HSP90AA1 PE=1 SV=5 |
| 137 | P08238\|HS90B_HUMAN | 40.17 | 83264 | Heat shock protein HSP 90-beta OS=Homo sapiens OX=9606 GN=HSP90AB1 PE=1 SV=4 |
| 138 | Q58FF8\|H90B2_HUMAN | 40.17 | 44349 | Putative heat shock protein HSP 90-beta 2 OS=Homo sapiens OX=9606 GN=HSP90AB2P PE=1 SV=2 |
| 139 | P13639\|EF2_HUMAN | 44.58 | 95338 | Elongation factor 2 OS=Homo sapiens OX=9606 GN=EEF2 PE=1 SV=4 |
| 140 | tr\|C9J6J8\|C9J6J8_HUMAN | 37.92 | 7827 | Sequestosome-1 OS=Homo sapiens OX=9606 GN=SQSTM1 PE=1 SV=2 |
| 141 | tr\|F8VZY9\|F8VZY9_HUMAN | 39.18 | 43774 | Keratin type I cytoskeletal 18 OS=Homo sapiens OX=9606 GN=KRT18 PE=1 SV=1 |
| 142 | tr\|G3V1Q4\|G3V1Q4_HUMAN | 39.58 | 46566 | Septin OS=Homo sapiens OX=9606 GN=SEPTIN7 PE=1 SV=2 |
| 143 | Q16181\|SEPT7_HUMAN | 39.58 | 50680 | Septin-7 OS=Homo sapiens OX=9606 GN=SEPTIN7 PE=1 SV=2 |
| 144 | tr\|A0A0D9SFE5\|A0A0D9SFE5_HUMAN | 37.07 | 37581 | Lamin B1 isoform CRA_a OS=Homo sapiens OX=9606 GN=LMNB1 PE=1 SV=1 |
| 145 | tr\|E9PBF6\|E9PBF6_HUMAN | 37.07 | 44643 | Lamin-B1 OS=Homo sapiens OX=9606 GN=LMNB1 PE=1 SV=1 |
| 146 | tr\|R4GN98\|R4GN98_HUMAN | 36.98 | 9681 | Protein S100 (Fragment) OS=Homo sapiens OX=9606 GN=S100A6 PE=1 SV=1 |
| 147 | P06703\|S10A6_HUMAN | 36.98 | 10180 | Protein S100-A6 OS=Homo sapiens OX=9606 GN=S100A6 PE=1 SV=1 |
| 148 | tr\|F5H6E2\|F5H6E2_HUMAN | 36.76 | 118994 | Unconventional myosin-Ic OS=Homo sapiens OX=9606 GN=MYO1C PE=1 SV=1 |
| 149 | P09669\|COX6C_HUMAN | 29.14 | 8781 | Cytochrome c oxidase subunit 6C OS=Homo sapiens OX=9606 GN=COX6C PE=1 SV=2 |

Supplementary table 2. List of 137 proteins that bind only to biotin and not to biotinylated KB2764.

| Number | Accession | -10lgP | Avg. Mass | Description |
| --- | --- | --- | --- | --- |
| 1 | P13645 | 310.94 | 58827 | Keratin type I cytoskeletal 10 OS=Homo sapiens OX=9606 GN=KRT10 PE=1 SV=6 |
| 2 | P68032 | 268.29 | 42019 | Actin alpha cardiac muscle 1 OS=Homo sapiens OX=9606 GN=ACTC1 PE=1 SV=1 |
| 3 | P62736 | 268.29 | 42009 | Actin aortic smooth muscle OS=Homo sapiens OX=9606 GN=ACTA2 PE=1 SV=1 |
| 4 | P35908 | 310.2 | 65433 | Keratin type II cytoskeletal 2 epidermal OS=Homo sapiens OX=9606 GN=KRT2 PE=1 SV=2 |
| 5 | P02533 | 223.49 | 51562 | Keratin type I cytoskeletal 14 OS=Homo sapiens OX=9606 GN=KRT14 PE=1 SV=4 |
| 6 | P08779 | 205.18 | 51268 | Keratin type I cytoskeletal 16 OS=Homo sapiens OX=9606 GN=KRT16 PE=1 SV=4 |
| 7 | P04259 | 198.54 | 60067 | Keratin type II cytoskeletal 6B OS=Homo sapiens OX=9606 GN=KRT6B PE=1 SV=5 |
| 8 | P35579 | 265.15 | 226530 | Myosin-9 OS=Homo sapiens OX=9606 GN=MYH9 PE=1 SV=4 |
| 9 | P13647 | 219.79 | 62378 | Keratin type II cytoskeletal 5 OS=Homo sapiens OX=9606 GN=KRT5 PE=1 SV=3 |
| 10 | P02538 | 215.06 | 60045 | Keratin type II cytoskeletal 6A OS=Homo sapiens OX=9606 GN=KRT6A PE=1 SV=3 |
| 11 | P29692 | 169.83 | 31122 | Elongation factor 1-delta OS=Homo sapiens OX=9606 GN=EEF1D PE=1 SV=5 |
| 12 | Q04695 | 183.6 | 48106 | Keratin type I cytoskeletal 17 OS=Homo sapiens OX=9606 GN=KRT17 PE=1 SV=2 |
| 13 | P24534 | 158.39 | 24764 | Elongation factor 1-beta OS=Homo sapiens OX=9606 GN=EEF1B2 PE=1 SV=3 |
| 14 | Q09666 | 236.52 | 629114 | Neuroblast differentiation-associated protein AHNAK OS=Homo sapiens OX=9606 GN=AHNAK PE=1 SV=2 |
| 15 | Q01546 | 137.47 | 65841 | Keratin type II cytoskeletal 2 oral OS=Homo sapiens OX=9606 GN=KRT76 PE=1 SV=2 |
| 16 | P07951 | 221.14 | 32851 | Tropomyosin beta chain OS=Homo sapiens OX=9606 GN=TPM2 PE=1 SV=1 |
| 17 | P05787 | 126.07 | 53704 | Keratin type II cytoskeletal 8 OS=Homo sapiens OX=9606 GN=KRT8 PE=1 SV=7 |
| 18 | P27487 | 155.85 | 88279 | Dipeptidyl peptidase 4 OS=Homo sapiens OX=9606 GN=DPP4 PE=1 SV=2 |
| 19 | Q9UII2 | 77.45 | 12249 | ATPase inhibitor mitochondrial OS=Homo sapiens OX=9606 GN=ATP5IF1 PE=1 SV=1 |
| 20 | P23396 | 185.49 | 26688 | 40S ribosomal protein S3 OS=Homo sapiens OX=9606 GN=RPS3 PE=1 SV=2 |
| 21 | P12035 | 130.95 | 64417 | Keratin type II cytoskeletal 3 OS=Homo sapiens OX=9606 GN=KRT3 PE=1 SV=3 |
| 22 | P13646 | 129.34 | 49588 | Keratin type I cytoskeletal 13 OS=Homo sapiens OX=9606 GN=KRT13 PE=1 SV=4 |
| 23 | Q15155 | 183.16 | 134324 | BOS complex subunit NOMO1 OS=Homo sapiens OX=9606 GN=NOMO1 PE=1 SV=5 |
| 24 | P02751 | 180.16 | 272318 | Fibronectin OS=Homo sapiens OX=9606 GN=FN1 PE=1 SV=5 |
| 25 | P06748 | 114.09 | 32575 | Nucleophosmin OS=Homo sapiens OX=9606 GN=NPM1 PE=1 SV=2 |
| 26 | P09012 | 96.24 | 31280 | U1 small nuclear ribonucleoprotein A OS=Homo sapiens OX=9606 GN=SNRPA PE=1 SV=3 |
| 27 | P60228 | 139.88 | 52221 | Eukaryotic translation initiation factor 3 subunit E OS=Homo sapiens OX=9606 GN=EIF3E PE=1 SV=1 |
| 28 | P35268 | 124.96 | 14787 | 60S ribosomal protein L22 OS=Homo sapiens OX=9606 GN=RPL22 PE=1 SV=2 |
| 29 | Q99470 | 116.38 | 23026 | Stromal cell-derived factor 2 OS=Homo sapiens OX=9606 GN=SDF2 PE=1 SV=2 |
| 30 | P38646 | 158.07 | 73681 | Stress-70 protein mitochondrial OS=Homo sapiens OX=9606 GN=HSPA9 PE=1 SV=2 |
| 31 | P47756 | 128.75 | 30629 | F-actin-capping protein subunit beta OS=Homo sapiens OX=9606 GN=CAPZB PE=1 SV=5 |
| 32 | Q9H2D6 | 124.5 | 261373 | TRIO and F-actin-binding protein OS=Homo sapiens OX=9606 GN=TRIOBP PE=1 SV=3 |
| 33 | Q86UP2 | 120.24 | 156275 | Kinectin OS=Homo sapiens OX=9606 GN=KTN1 PE=1 SV=1 |
| 34 | Q9Y224 | 122.33 | 28068 | RNA transcription translation and transport factor protein OS=Homo sapiens OX=9606 GN=RTRAF PE=1 SV=1 |
| 35 | O15372 | 147.87 | 39930 | Eukaryotic translation initiation factor 3 subunit H OS=Homo sapiens OX=9606 GN=EIF3H PE=1 SV=1 |
| 36 | Q16531 | 129.9 | 126968 | DNA damage-binding protein 1 OS=Homo sapiens OX=9606 GN=DDB1 PE=1 SV=1 |
| 37 | P11021 | 90.08 | 72333 | Endoplasmic reticulum chaperone BiP OS=Homo sapiens OX=9606 GN=HSPA5 PE=1 SV=2 |
| 38 | Q86YZ3 | 89.68 | 282389 | Hornerin OS=Homo sapiens OX=9606 GN=HRNR PE=1 SV=2 |
| 39 | P14868 | 95.79 | 57136 | Aspartate--tRNA ligase cytoplasmic OS=Homo sapiens OX=9606 GN=DARS1 PE=1 SV=2 |
| 40 | P07093 | 137.54 | 44002 | Glia-derived nexin OS=Homo sapiens OX=9606 GN=SERPINE2 PE=1 SV=1 |
| 41 | P52907 | 158.42 | 32923 | F-actin-capping protein subunit alpha-1 OS=Homo sapiens OX=9606 GN=CAPZA1 PE=1 SV=3 |
| 42 | P08579 | 84.82 | 25486 | U2 small nuclear ribonucleoprotein B'' OS=Homo sapiens OX=9606 GN=SNRPB2 PE=1 SV=1 |
| 43 | P62913 | 145.56 | 20252 | 60S ribosomal protein L11 OS=Homo sapiens OX=9606 GN=RPL11 PE=1 SV=2 |
| 44 | P67809 | 136.34 | 35924 | Y-box-binding protein 1 OS=Homo sapiens OX=9606 GN=YBX1 PE=1 SV=3 |
| 45 | P07996 | 88.68 | 129383 | Thrombospondin-1 OS=Homo sapiens OX=9606 GN=THBS1 PE=1 SV=2 |
| 46 | P01040 | 73.1 | 11006 | Cystatin-A OS=Homo sapiens OX=9606 GN=CSTA PE=1 SV=1 |
| 47 | P84090 | 67.7 | 12259 | Enhancer of rudimentary homolog OS=Homo sapiens OX=9606 GN=ERH PE=1 SV=1 |
| 48 | P81605 | 63.09 | 11284 | Dermcidin OS=Homo sapiens OX=9606 GN=DCD PE=1 SV=2 |
| 49 | O75339 | 34 | 132565 | Cartilage intermediate layer protein 1 OS=Homo sapiens OX=9606 GN=CILP PE=1 SV=4 |
| 50 | Q969R2 | 26.94 | 101266 | Oxysterol-binding protein 2 OS=Homo sapiens OX=9606 GN=OSBP2 PE=1 SV=2 |
| 51 | P15924 | 56 | 331774 | Desmoplakin OS=Homo sapiens OX=9606 GN=DSP PE=1 SV=3 |
| 52 | P21980 | 70.7 | 77329 | Protein-glutamine gamma-glutamyltransferase 2 OS=Homo sapiens OX=9606 GN=TGM2 PE=1 SV=2 |
| 53 | P05362 | 100.31 | 57825 | Intercellular adhesion molecule 1 OS=Homo sapiens OX=9606 GN=ICAM1 PE=1 SV=2 |
| 54 | P0C0S8 | 57.94 | 14091 | Histone H2A type 1 OS=Homo sapiens OX=9606 GN=H2AC11 PE=1 SV=2 |
| 55 | P04908 | 57.94 | 14135 | Histone H2A type 1-B/E OS=Homo sapiens OX=9606 GN=H2AC4 PE=1 SV=2 |
| 56 | Q7L7L0 | 57.94 | 14121 | Histone H2A type 3 OS=Homo sapiens OX=9606 GN=H2AC25 PE=1 SV=3 |
| 57 | Q9BTM1 | 57.94 | 14019 | Histone H2A.J OS=Homo sapiens OX=9606 GN=H2AJ PE=1 SV=1 |
| 58 | P16104 | 57.94 | 15145 | Histone H2AX OS=Homo sapiens OX=9606 GN=H2AX PE=1 SV=2 |
| 59 | Q8TD57 | 27.52 | 470774 | Dynein axonemal heavy chain 3 OS=Homo sapiens OX=9606 GN=DNAH3 PE=2 SV=1 |
| 60 | Q9P0W8 | 26.76 | 67719 | Spermatogenesis-associated protein 7 OS=Homo sapiens OX=9606 GN=SPATA7 PE=1 SV=3 |
| 61 | P41091 | 90.4 | 51110 | Eukaryotic translation initiation factor 2 subunit 3 OS=Homo sapiens OX=9606 GN=EIF2S3 PE=1 SV=3 |
| 62 | Q2VIR3 | 90.4 | 51229 | Eukaryotic translation initiation factor 2 subunit 3B OS=Homo sapiens OX=9606 GN=EIF2S3B PE=2 SV=2 |
| 63 | P52926 | 78.6 | 11832 | High mobility group protein HMGI-C OS=Homo sapiens OX=9606 GN=HMGA2 PE=1 SV=1 |
| 64 | O43324 | 77.43 | 19811 | Eukaryotic translation elongation factor 1 epsilon-1 OS=Homo sapiens OX=9606 GN=EEF1E1 PE=1 SV=1 |
| 65 | Q99623 | 76.99 | 33296 | Prohibitin-2 OS=Homo sapiens OX=9606 GN=PHB2 PE=1 SV=2 |
| 66 | P46821 | 86.69 | 270632 | Microtubule-associated protein 1B OS=Homo sapiens OX=9606 GN=MAP1B PE=1 SV=2 |
| 67 | P0CG47 | 89.51 | 25762 | Polyubiquitin-B OS=Homo sapiens OX=9606 GN=UBB PE=1 SV=1 |
| 68 | P0CG48 | 89.51 | 77039 | Polyubiquitin-C OS=Homo sapiens OX=9606 GN=UBC PE=1 SV=3 |
| 69 | P62979 | 89.51 | 17965 | Ubiquitin-40S ribosomal protein S27a OS=Homo sapiens OX=9606 GN=RPS27A PE=1 SV=2 |
| 70 | P62987 | 89.51 | 14728 | Ubiquitin-60S ribosomal protein L40 OS=Homo sapiens OX=9606 GN=UBA52 PE=1 SV=2 |
| 71 | P28331 | 57.03 | 79468 | NADH-ubiquinone oxidoreductase 75 kDa subunit mitochondrial OS=Homo sapiens OX=9606 GN=NDUFS1 PE=1 SV=3 |
| 72 | Q16777 | 55.9 | 13988 | Histone H2A type 2-C OS=Homo sapiens OX=9606 GN=H2AC20 PE=1 SV=4 |
| 73 | Q92572 | 60.06 | 21732 | AP-3 complex subunit sigma-1 OS=Homo sapiens OX=9606 GN=AP3S1 PE=1 SV=1 |
| 74 | P62851 | 63.56 | 13742 | 40S ribosomal protein S25 OS=Homo sapiens OX=9606 GN=RPS25 PE=1 SV=1 |
| 75 | Q9UHB6 | 54.99 | 85226 | LIM domain and actin-binding protein 1 OS=Homo sapiens OX=9606 GN=LIMA1 PE=1 SV=1 |
| 76 | P62829 | 52.27 | 14865 | 60S ribosomal protein L23 OS=Homo sapiens OX=9606 GN=RPL23 PE=1 SV=1 |
| 77 | P62826 | 42.54 | 24423 | GTP-binding nuclear protein Ran OS=Homo sapiens OX=9606 GN=RAN PE=1 SV=3 |
| 78 | P61009 | 44.54 | 20313 | Signal peptidase complex subunit 3 OS=Homo sapiens OX=9606 GN=SPCS3 PE=1 SV=1 |
| 79 | Q9Y262 | 47.27 | 66727 | Eukaryotic translation initiation factor 3 subunit L OS=Homo sapiens OX=9606 GN=EIF3L PE=1 SV=1 |
| 80 | P05121 | 38.27 | 45060 | Plasminogen activator inhibitor 1 OS=Homo sapiens OX=9606 GN=SERPINE1 PE=1 SV=1 |
| 81 | P07858 | 46.45 | 37822 | Cathepsin B OS=Homo sapiens OX=9606 GN=CTSB PE=1 SV=3 |
| 82 | P49458 | 35.25 | 10112 | Signal recognition particle 9 kDa protein OS=Homo sapiens OX=9606 GN=SRP9 PE=1 SV=2 |
| 83 | P09038 | 34.92 | 30770 | Fibroblast growth factor 2 OS=Homo sapiens OX=9606 GN=FGF2 PE=1 SV=3 |
| 84 | Q99460 | 23.86 | 105836 | 26S proteasome non-ATPase regulatory subunit 1 OS=Homo sapiens OX=9606 GN=PSMD1 PE=1 SV=2 |
| 85 | O43290 | 23.78 | 90255 | U4/U6.U5 tri-snRNP-associated protein 1 OS=Homo sapiens OX=9606 GN=SART1 PE=1 SV=1 |
| 86 | Q93070 | 23.65 | 35878 | Ecto-ADP-ribosyltransferase 4 OS=Homo sapiens OX=9606 GN=ART4 PE=2 SV=2 |
| 87 | Q68D51 | 23.48 | 106865 | DENN domain-containing protein 2C OS=Homo sapiens OX=9606 GN=DENND2C PE=1 SV=2 |
| 88 | Q9NZR1 | 52.62 | 39595 | Tropomodulin-2 OS=Homo sapiens OX=9606 GN=TMOD2 PE=1 SV=1 |
| 89 | Q9NYL9 | 52.62 | 39595 | Tropomodulin-3 OS=Homo sapiens OX=9606 GN=TMOD3 PE=1 SV=1 |
| 90 | Q8NDY3 | 25.07 | 40105 | Inactive ADP-ribosyltransferase ARH2 OS=Homo sapiens OX=9606 GN=ADPRHL1 PE=2 SV=1 |
| 91 | P31944 | 57.31 | 27680 | Caspase-14 OS=Homo sapiens OX=9606 GN=CASP14 PE=1 SV=2 |
| 92 | P62081 | 23.57 | 22127 | 40S ribosomal protein S7 OS=Homo sapiens OX=9606 GN=RPS7 PE=1 SV=1 |
| 93 | P62873 | 41.74 | 37377 | Guanine nucleotide-binding protein G(I)/G(S)/G(T) subunit beta-1 OS=Homo sapiens OX=9606 GN=GNB1 PE=1 SV=3 |
| 94 | P62879 | 41.74 | 37331 | Guanine nucleotide-binding protein G(I)/G(S)/G(T) subunit beta-2 OS=Homo sapiens OX=9606 GN=GNB2 PE=1 SV=3 |
| 95 | P16520 | 41.74 | 37221 | Guanine nucleotide-binding protein G(I)/G(S)/G(T) subunit beta-3 OS=Homo sapiens OX=9606 GN=GNB3 PE=1 SV=1 |
| 96 | Q9HAV0 | 41.74 | 37567 | Guanine nucleotide-binding protein subunit beta-4 OS=Homo sapiens OX=9606 GN=GNB4 PE=1 SV=3 |
| 97 | Q4J6C6 | 25.36 | 83927 | Prolyl endopeptidase-like OS=Homo sapiens OX=9606 GN=PREPL PE=1 SV=1 |
| 98 | P00338 | 75.53 | 36689 | L-lactate dehydrogenase A chain OS=Homo sapiens OX=9606 GN=LDHA PE=1 SV=2 |
| 99 | Q9BYZ2 | 75.53 | 41943 | L-lactate dehydrogenase A-like 6B OS=Homo sapiens OX=9606 GN=LDHAL6B PE=1 SV=3 |
| 100 | Q9BZL4 | 95.12 | 84881 | Protein phosphatase 1 regulatory subunit 12C OS=Homo sapiens OX=9606 GN=PPP1R12C PE=1 SV=1 |
| 101 | P80723 | 68.89 | 22693 | Brain acid soluble protein 1 OS=Homo sapiens OX=9606 GN=BASP1 PE=1 SV=2 |
| 102 | P61604 | 66.55 | 10932 | 10 kDa heat shock protein mitochondrial OS=Homo sapiens OX=9606 GN=HSPE1 PE=1 SV=2 |
| 103 | Q5D862 | 79.15 | 248072 | Filaggrin-2 OS=Homo sapiens OX=9606 GN=FLG2 PE=1 SV=1 |
| 104 | P60903 | 59.92 | 11203 | Protein S100-A10 OS=Homo sapiens OX=9606 GN=S100A10 PE=1 SV=2 |
| 105 | Q92973 | 63.79 | 102355 | Transportin-1 OS=Homo sapiens OX=9606 GN=TNPO1 PE=1 SV=2 |
| 106 | Q01658 | 51.64 | 19444 | Protein Dr1 OS=Homo sapiens OX=9606 GN=DR1 PE=1 SV=1 |
| 107 | P62269 | 56.87 | 17719 | 40S ribosomal protein S18 OS=Homo sapiens OX=9606 GN=RPS18 PE=1 SV=3 |
| 108 | P26373 | 43.01 | 24261 | 60S ribosomal protein L13 OS=Homo sapiens OX=9606 GN=RPL13 PE=1 SV=4 |
| 109 | P41227 | 45.15 | 26458 | N-alpha-acetyltransferase 10 OS=Homo sapiens OX=9606 GN=NAA10 PE=1 SV=1 |
| 110 | Q9BSU3 | 45.15 | 25979 | N-alpha-acetyltransferase 11 OS=Homo sapiens OX=9606 GN=NAA11 PE=1 SV=3 |
| 111 | Q16718 | 42.69 | 13459 | NADH dehydrogenase [ubiquinone] 1 alpha subcomplex subunit 5 OS=Homo sapiens OX=9606 GN=NDUFA5 PE=1 SV=3 |
| 112 | Q96JB5 | 47.1 | 56921 | CDK5 regulatory subunit-associated protein 3 OS=Homo sapiens OX=9606 GN=CDK5RAP3 PE=1 SV=2 |
| 113 | P20810 | 51.58 | 76573 | Calpastatin OS=Homo sapiens OX=9606 GN=CAST PE=1 SV=4 |
| 114 | Q86UE4 | 43.45 | 63837 | Protein LYRIC OS=Homo sapiens OX=9606 GN=MTDH PE=1 SV=2 |
| 115 | Q01469 | 40.34 | 15164 | Fatty acid-binding protein 5 OS=Homo sapiens OX=9606 GN=FABP5 PE=1 SV=3 |
| 116 | Q9HCN8 | 42.45 | 23598 | Stromal cell-derived factor 2-like protein 1 OS=Homo sapiens OX=9606 GN=SDF2L1 PE=1 SV=2 |
| 117 | Q9NVD7 | 48.62 | 42244 | Alpha-parvin OS=Homo sapiens OX=9606 GN=PARVA PE=1 SV=1 |
| 118 | P63241 | 39.98 | 16832 | Eukaryotic translation initiation factor 5A-1 OS=Homo sapiens OX=9606 GN=EIF5A PE=1 SV=2 |
| 119 | Q9GZV4 | 39.98 | 16793 | Eukaryotic translation initiation factor 5A-2 OS=Homo sapiens OX=9606 GN=EIF5A2 PE=1 SV=3 |
| 120 | P31946 | 33.01 | 28082 | 14-3-3 protein beta/alpha OS=Homo sapiens OX=9606 GN=YWHAB PE=1 SV=3 |
| 121 | Q04917 | 33.01 | 28219 | 14-3-3 protein eta OS=Homo sapiens OX=9606 GN=YWHAH PE=1 SV=4 |
| 122 | P61981 | 33.01 | 28303 | 14-3-3 protein gamma OS=Homo sapiens OX=9606 GN=YWHAG PE=1 SV=2 |
| 123 | P31947 | 33.01 | 27774 | 14-3-3 protein sigma OS=Homo sapiens OX=9606 GN=SFN PE=1 SV=1 |
| 124 | Q96MM6 | 30.68 | 75688 | Heat shock 70 kDa protein 12B OS=Homo sapiens OX=9606 GN=HSPA12B PE=1 SV=2 |
| 125 | O43583 | 30.99 | 22092 | Density-regulated protein OS=Homo sapiens OX=9606 GN=DENR PE=1 SV=2 |
| 126 | O60911 | 30.95 | 37329 | Cathepsin L2 OS=Homo sapiens OX=9606 GN=CTSV PE=1 SV=2 |
| 127 | P07711 | 30.95 | 37564 | Procathepsin L OS=Homo sapiens OX=9606 GN=CTSL PE=1 SV=2 |
| 128 | Q5NE16 | 30.95 | 25059 | Putative inactive cathepsin L-like protein CTSL3P OS=Homo sapiens OX=9606 GN=CTSL3P PE=5 SV=1 |
| 129 | P09496 | 29.89 | 27077 | Clathrin light chain A OS=Homo sapiens OX=9606 GN=CLTA PE=1 SV=1 |
| 130 | P08123 | 29.93 | 129314 | Collagen alpha-2(I) chain OS=Homo sapiens OX=9606 GN=COL1A2 PE=1 SV=7 |
| 131 | Q08378 | 33.9 | 167354 | Golgin subfamily A member 3 OS=Homo sapiens OX=9606 GN=GOLGA3 PE=1 SV=2 |
| 132 | O75821 | 27.28 | 35611 | Eukaryotic translation initiation factor 3 subunit G OS=Homo sapiens OX=9606 GN=EIF3G PE=1 SV=2 |
| 133 | P62899 | 25.61 | 14463 | 60S ribosomal protein L31 OS=Homo sapiens OX=9606 GN=RPL31 PE=1 SV=1 |
| 134 | O15511 | 25.17 | 16320 | Actin-related protein 2/3 complex subunit 5 OS=Homo sapiens OX=9606 GN=ARPC5 PE=1 SV=3 |
| 135 | P07477 | 24.99 | 26558 | Serine protease 1 OS=Homo sapiens OX=9606 GN=PRSS1 PE=1 SV=1 |
| 136 | Q9BYE2 | 24.99 | 63167 | Transmembrane protease serine 13 OS=Homo sapiens OX=9606 GN=TMPRSS13 PE=2 SV=5 |
| 137 | Q86SQ0 | 23.28 | 142158 | Pleckstrin homology-like domain family B member 2 OS=Homo sapiens OX=9606 GN=PHLDB2 PE=1 SV=2 |

Supplementary table 3. List of 48 proteins that bind to both biotinylated KB2764 and biotin.

| Number | Accession | -10lgP | Avg. Mass | Description |
| --- | --- | --- | --- | --- |
| 1 | P04264 | 357.11 | 66039 | Keratin type II cytoskeletal 1 OS=Homo sapiens OX=9606 GN=KRT1 PE=1 SV=6 |
| 2 | P60709 | 296.4 | 41737 | Actin cytoplasmic 1 OS=Homo sapiens OX=9606 GN=ACTB PE=1 SV=1 |
| 3 | P63261 | 296.4 | 41793 | Actin cytoplasmic 2 OS=Homo sapiens OX=9606 GN=ACTG1 PE=1 SV=1 |
| 4 | P08670 | 284.81 | 53652 | Vimentin OS=Homo sapiens OX=9606 GN=VIM PE=1 SV=4 |
| 5 | P35527 | 307.16 | 62064 | Keratin type I cytoskeletal 9 OS=Homo sapiens OX=9606 GN=KRT9 PE=1 SV=3 |
| 6 | P02768 | 202.14 | 69367 | Albumin OS=Homo sapiens OX=9606 GN=ALB PE=1 SV=2 |
| 7 | P07355 | 249.04 | 38604 | Annexin A2 OS=Homo sapiens OX=9606 GN=ANXA2 PE=1 SV=2 |
| 8 | Q05682 | 228.52 | 93231 | Caldesmon OS=Homo sapiens OX=9606 GN=CALD1 PE=1 SV=3 |
| 9 | Q9P2E9 | 279.99 | 152456 | Ribosome-binding protein 1 OS=Homo sapiens OX=9606 GN=RRBP1 PE=1 SV=5 |
| 10 | P09493 | 227.64 | 32709 | Tropomyosin alpha-1 chain OS=Homo sapiens OX=9606 GN=TPM1 PE=1 SV=2 |
| 11 | P67936 | 195.98 | 28522 | Tropomyosin alpha-4 chain OS=Homo sapiens OX=9606 GN=TPM4 PE=1 SV=3 |
| 12 | P06753 | 182.44 | 32950 | Tropomyosin alpha-3 chain OS=Homo sapiens OX=9606 GN=TPM3 PE=1 SV=2 |
| 13 | P05198 | 162.65 | 36112 | Eukaryotic translation initiation factor 2 subunit 1 OS=Homo sapiens OX=9606 GN=EIF2S1 PE=1 SV=3 |
| 14 | P23284 | 169.24 | 23743 | Peptidyl-prolyl cis-trans isomerase B OS=Homo sapiens OX=9606 GN=PPIB PE=1 SV=2 |
| 15 | Q9UHD8 | 184.11 | 65402 | Septin-9 OS=Homo sapiens OX=9606 GN=SEPTIN9 PE=1 SV=2 |
| 16 | Q13347 | 159.16 | 36502 | Eukaryotic translation initiation factor 3 subunit I OS=Homo sapiens OX=9606 GN=EIF3I PE=1 SV=1 |
| 17 | P68104 | 158.21 | 50141 | Elongation factor 1-alpha 1 OS=Homo sapiens OX=9606 GN=EEF1A1 PE=1 SV=1 |
| 18 | Q5VTE0 | 158.21 | 50185 | Putative elongation factor 1-alpha-like 3 OS=Homo sapiens OX=9606 GN=EEF1A1P5 PE=5 SV=1 |
| 19 | Q14956 | 65.75 | 63923 | Transmembrane glycoprotein NMB OS=Homo sapiens OX=9606 GN=GPNMB PE=1 SV=2 |
| 20 | Q15019 | 136.36 | 41487 | Septin-2 OS=Homo sapiens OX=9606 GN=SEPTIN2 PE=1 SV=1 |
| 21 | Q9NVA2 | 186.59 | 49398 | Septin-11 OS=Homo sapiens OX=9606 GN=SEPTIN11 PE=1 SV=3 |
| 22 | P60660 | 111.11 | 16930 | Myosin light polypeptide 6 OS=Homo sapiens OX=9606 GN=MYL6 PE=1 SV=2 |
| 23 | Q06830 | 128.76 | 22110 | Peroxiredoxin-1 OS=Homo sapiens OX=9606 GN=PRDX1 PE=1 SV=1 |
| 24 | P07237 | 113.59 | 57116 | Protein disulfide-isomerase OS=Homo sapiens OX=9606 GN=P4HB PE=1 SV=3 |
| 25 | Q13813 | 144.23 | 284538 | Spectrin alpha chain non-erythrocytic 1 OS=Homo sapiens OX=9606 GN=SPTAN1 PE=1 SV=3 |
| 26 | P27816 | 159.94 | 121005 | Microtubule-associated protein 4 OS=Homo sapiens OX=9606 GN=MAP4 PE=1 SV=3 |
| 27 | P39019 | 105.4 | 16060 | 40S ribosomal protein S19 OS=Homo sapiens OX=9606 GN=RPS19 PE=1 SV=2 |
| 28 | P06576 | 99 | 56560 | ATP synthase subunit beta mitochondrial OS=Homo sapiens OX=9606 GN=ATP5F1B PE=1 SV=3 |
| 29 | P32119 | 98.35 | 21892 | Peroxiredoxin-2 OS=Homo sapiens OX=9606 GN=PRDX2 PE=1 SV=5 |
| 30 | P11142 | 114.34 | 70898 | Heat shock cognate 71 kDa protein OS=Homo sapiens OX=9606 GN=HSPA8 PE=1 SV=1 |
| 31 | Q15393 | 97.6 | 135577 | Splicing factor 3B subunit 3 OS=Homo sapiens OX=9606 GN=SF3B3 PE=1 SV=4 |
| 32 | P19105 | 96.01 | 19794 | Myosin regulatory light chain 12A OS=Homo sapiens OX=9606 GN=MYL12A PE=1 SV=2 |
| 33 | O14950 | 96.01 | 19779 | Myosin regulatory light chain 12B OS=Homo sapiens OX=9606 GN=MYL12B PE=1 SV=2 |
| 34 | P62263 | 103.83 | 16273 | 40S ribosomal protein S14 OS=Homo sapiens OX=9606 GN=RPS14 PE=1 SV=3 |
| 35 | Q0ZGT2 | 77.46 | 80658 | Nexilin OS=Homo sapiens OX=9606 GN=NEXN PE=1 SV=1 |
| 36 | Q14247 | 81.07 | 61586 | Src substrate cortactin OS=Homo sapiens OX=9606 GN=CTTN PE=1 SV=2 |
| 37 | P23528 | 51.35 | 18502 | Cofilin-1 OS=Homo sapiens OX=9606 GN=CFL1 PE=1 SV=3 |
| 38 | P04406 | 111.55 | 36053 | Glyceraldehyde-3-phosphate dehydrogenase OS=Homo sapiens OX=9606 GN=GAPDH PE=1 SV=3 |
| 39 | P62857 | 55.02 | 7841 | 40S ribosomal protein S28 OS=Homo sapiens OX=9606 GN=RPS28 PE=1 SV=1 |
| 40 | Q6NZI2 | 32.29 | 43476 | Caveolae-associated protein 1 OS=Homo sapiens OX=9606 GN=CAVIN1 PE=1 SV=1 |
| 41 | P10809 | 84.92 | 61055 | 60 kDa heat shock protein mitochondrial OS=Homo sapiens OX=9606 GN=HSPD1 PE=1 SV=2 |
| 42 | P02545 | 47.11 | 74140 | Prelamin-A/C OS=Homo sapiens OX=9606 GN=LMNA PE=1 SV=1 |
| 43 | P12235 | 38.35 | 33065 | ADP/ATP translocase 1 OS=Homo sapiens OX=9606 GN=SLC25A4 PE=1 SV=4 |
| 44 | P05141 | 38.35 | 32852 | ADP/ATP translocase 2 OS=Homo sapiens OX=9606 GN=SLC25A5 PE=1 SV=7 |
| 45 | P12236 | 38.35 | 32866 | ADP/ATP translocase 3 OS=Homo sapiens OX=9606 GN=SLC25A6 PE=1 SV=4 |
| 46 | Q9H0C2 | 38.35 | 35022 | ADP/ATP translocase 4 OS=Homo sapiens OX=9606 GN=SLC25A31 PE=1 SV=1 |
| 47 | P27348 | 33.01 | 27764 | 14-3-3 protein theta OS=Homo sapiens OX=9606 GN=YWHAQ PE=1 SV=1 |
| 48 | P43235 | 30.95 | 36966 | Cathepsin K OS=Homo sapiens OX=9606 GN=CTSK PE=1 SV=1 |

**Reference**

1. Lee YH, Choi D, Jang G, et al. Targeting regulation of ATP synthase 5 alpha/beta dimerization alleviates senescence. *Aging (Albany NY).* 2022;14(2):678-707.

2. Wei J-D, Kim J-Y, Kim A-K, Jang SK, Kim J-H. RanBPM Protein Acts as a Negative Regulator of BLT2 Receptor to Attenuate BLT2-mediated Cell Motility. *The Journal of Biological Chemistry.* 2013;288(37):26753-26763.

3. Hwang SY, Kuk MU, Kim JW, et al. ATM mediated-p53 signaling pathway forms a novel axis for senescence control. *Mitochondrion.* 2020;55:54-63.

4. Kang HT, Park JT, Choi K, et al. Chemical screening identifies ATM as a target for alleviating senescence. *Nat Chem Biol.* 2017;13(6):616-623.
